# Targeted delivery to macrophages and dendritic cells by chemically modified mannose ligand-conjugated siRNA

**DOI:** 10.1093/nar/gkac308

**Published:** 2022-05-07

**Authors:** Keiji Uehara, Toshimasa Harumoto, Asana Makino, Yasuo Koda, Junko Iwano, Yasuhiro Suzuki, Mari Tanigawa, Hiroto Iwai, Kana Asano, Kana Kurihara, Akinori Hamaguchi, Hiroshi Kodaira, Toshiyuki Atsumi, Yoji Yamada, Kazuma Tomizuka

**Affiliations:** Research Unit, R&D Division, Kyowa Kirin Co., Ltd., 3-6-6, Otemachi Financial City Grand Cube, 1-9-2 Otemachi, Chiyoda-ku, Tokyo 100-0004, Japan; Research Unit, R&D Division, Kyowa Kirin Co., Ltd., 3-6-6, Otemachi Financial City Grand Cube, 1-9-2 Otemachi, Chiyoda-ku, Tokyo 100-0004, Japan; Research Unit, R&D Division, Kyowa Kirin Co., Ltd., 3-6-6, Otemachi Financial City Grand Cube, 1-9-2 Otemachi, Chiyoda-ku, Tokyo 100-0004, Japan; Research Unit, R&D Division, Kyowa Kirin Co., Ltd., 3-6-6, Otemachi Financial City Grand Cube, 1-9-2 Otemachi, Chiyoda-ku, Tokyo 100-0004, Japan; Translational Research Unit, R&D Division, Kyowa Kirin Co., Ltd., 1188 Shimotogari, Nagaizumi-cho, Sunto-gun, Shizuoka 411-8731, Japan; Research Unit, R&D Division, Kyowa Kirin Co., Ltd., 3-6-6, Otemachi Financial City Grand Cube, 1-9-2 Otemachi, Chiyoda-ku, Tokyo 100-0004, Japan; Research Unit, R&D Division, Kyowa Kirin Co., Ltd., 3-6-6, Otemachi Financial City Grand Cube, 1-9-2 Otemachi, Chiyoda-ku, Tokyo 100-0004, Japan; Research Unit, R&D Division, Kyowa Kirin Co., Ltd., 3-6-6, Otemachi Financial City Grand Cube, 1-9-2 Otemachi, Chiyoda-ku, Tokyo 100-0004, Japan; Research Unit, R&D Division, Kyowa Kirin Co., Ltd., 3-6-6, Otemachi Financial City Grand Cube, 1-9-2 Otemachi, Chiyoda-ku, Tokyo 100-0004, Japan; Research Unit, R&D Division, Kyowa Kirin Co., Ltd., 3-6-6, Otemachi Financial City Grand Cube, 1-9-2 Otemachi, Chiyoda-ku, Tokyo 100-0004, Japan; Research Unit, R&D Division, Kyowa Kirin Co., Ltd., 3-6-6, Otemachi Financial City Grand Cube, 1-9-2 Otemachi, Chiyoda-ku, Tokyo 100-0004, Japan; Translational Research Unit, R&D Division, Kyowa Kirin Co., Ltd., 1188 Shimotogari, Nagaizumi-cho, Sunto-gun, Shizuoka 411-8731, Japan; Research Unit, R&D Division, Kyowa Kirin Co., Ltd., 3-6-6, Otemachi Financial City Grand Cube, 1-9-2 Otemachi, Chiyoda-ku, Tokyo 100-0004, Japan; Research Unit, R&D Division, Kyowa Kirin Co., Ltd., 3-6-6, Otemachi Financial City Grand Cube, 1-9-2 Otemachi, Chiyoda-ku, Tokyo 100-0004, Japan; Research Unit, R&D Division, Kyowa Kirin Co., Ltd., 3-6-6, Otemachi Financial City Grand Cube, 1-9-2 Otemachi, Chiyoda-ku, Tokyo 100-0004, Japan

## Abstract

Extrahepatic delivery of small interfering RNAs (siRNAs) may have applications in the development of novel therapeutic approaches. However, reports on such approaches are limited, and the scarcity of reports concerning the systemically targeted delivery of siRNAs with effective gene silencing activity presents a challenge. We herein report for the first time the targeted delivery of CD206-targetable chemically modified mannose–siRNA (CMM–siRNA) conjugates to macrophages and dendritic cells (DCs). CMM–siRNA exhibited a strong binding ability to CD206 and selectively delivered contents to CD206-expressing macrophages and DCs. Furthermore, the conjugates demonstrated strong gene silencing ability with long-lasting effects and protein downregulation in CD206-expressing cells *in vivo*. These findings could broaden the use of siRNA technology, provide additional therapeutic opportunities, and establish a basis for further innovative approaches for the targeted delivery of siRNAs to not only macrophages and DCs but also other cell types.

## INTRODUCTION

Nucleic acid-based medicines using small interfering RNAs (siRNAs) have potential applications in the treatment of human diseases, such as cancers, viral infection, and genetic disorders (1–3). siRNAs have great potential to affect targets traditionally considered undruggable when using classical small-molecule approaches or biologics (4–6).

Because siRNAs exhibit poor cell penetration and are unstable under serum and intracellular conditions, various siRNA delivery platforms, such as polymer- and lipid-based nanoparticles and ligand-conjugated siRNAs, have been developed to overcome these issues (7–10). Recently developed platforms include lipid nanoparticle-formulated siRNAs (LNPs) and *N*-acetylgalactosamine (GalNAc) ligand-conjugated siRNAs (GalNAc–siRNAs). The representative LNP Patisiran and the GalNAc–siRNA Givosiran have been approved by the Food and Drug Administration for the treatment of hereditary transthyretin amyloidosis and acute hepatic porphyria, respectively (11,12). In particular, GalNAc–siRNA, which consists of a GalNAc ligand that targets the asialoglycoprotein receptor (ASGPR) on hepatocytes and metabolically stabilized siRNA, can silence the target gene in hepatocytes as a simple molecular construct (13,14); it is now being evaluated in several late-stage trials (1,12). Thus, receptor-mediated targeted delivery using ligand-conjugated siRNAs, such as GalNAc–siRNA, is expected to be a promising strategy for delivering drugs based on their ability to selectively target desired cells, thereby resulting in dramatic enhancement of the desired effect with minimal side effects (15).

However, receptor-mediated targeted delivery of siRNA outside the liver using ligand-conjugated siRNAs remains challenging (16). Several groups have attempted to selectively deliver siRNAs to tumor cells using antibodies, their fragments, nanobodies, or scaffold protein-conjugated siRNAs (17–20). For muscle targeting, Sugo *et al.* reported the systemically targeted delivery of siRNA to muscle cells using anti-transferrin antibodies and fragment-conjugated siRNAs (21). Although the findings from these studies are encouraging, systemic *in vivo* targeted delivery with effective gene silencing and protein downregulation remains difficult. In addition, few reports have described applications using ligand-conjugated siRNAs targeting cells other than cancer and muscle cells.

Macrophages and dendritic cells (DCs) are important immune cells that link innate and acquired immunity. Macrophages, which have a strong phagocytic capacity, are distributed throughout the body and are involved in responses to the invasion of foreign antigens. DCs are potent antigen-presenting cells that not only induce T cell-dependent antigen-specific acquired immune responses but also contribute to innate immune responses (22–24). Therefore, DCs are deeply involved in the pathogenesis of various autoimmune diseases and cancer. For example, in autoimmune diseases such as systemic lupus erythematosus, rheumatoid arthritis, and ulcerative colitis, DCs lose their immunosuppressive function and exhibit a pro-inflammatory phenotype (25,26). Macrophages are thought to contribute to the maintenance of immune homeostasis by flexibly changing their properties to pro-inflammatory or pro-reparative. However, this balance is disrupted in the context of autoimmune disease, and macrophages at the lesion site produce cytokines that promote disease progression (27,28). In addition, both macrophages and DCs that accumulate in the vicinity of tumors may contribute to immune escape by cancer cells; therefore, these cells are attractive targets for new antitumor immunity-inducing drugs (29,30). Delivering siRNAs to macrophages and DCs to regulate gene expression is considered an attractive approach for the treatment of these diseases (31–36).

Given the success of GalNAc–siRNA and previous findings concerning extrahepatic targeting by ligand-conjugated siRNAs, one possible way to achieve strong gene silencing in target cells is via strong enhancement of the cellular uptake of the siRNA utilized by a suitable receptor/ligand set. From this viewpoint, targeting CD206 (a macrophage mannose receptor) using mannose as a natural ligand is considered a reasonable strategy for the following reasons. First, CD206 is an endocytic receptor that is expressed in most types of macrophages and DC subsets (37–39). Secondly, CD206 constitutively and rapidly recycles between the cell surface and endosomes (40); therefore, marked accumulation of ligand-conjugated siRNAs internalized by receptor-mediated endocytosis inside the cell is expected. However, few studies have reported attempts at *in vivo* targeted delivery of mannose ligand-conjugated siRNAs to cells that express CD206 (41). Because the natural CD206 ligand, i.e. mannose, has a low affinity to its binding proteins (42–44), the rational design of lower-valency constructs with high affinity has generally been met with limited success (45,46).

**Figure 1.**
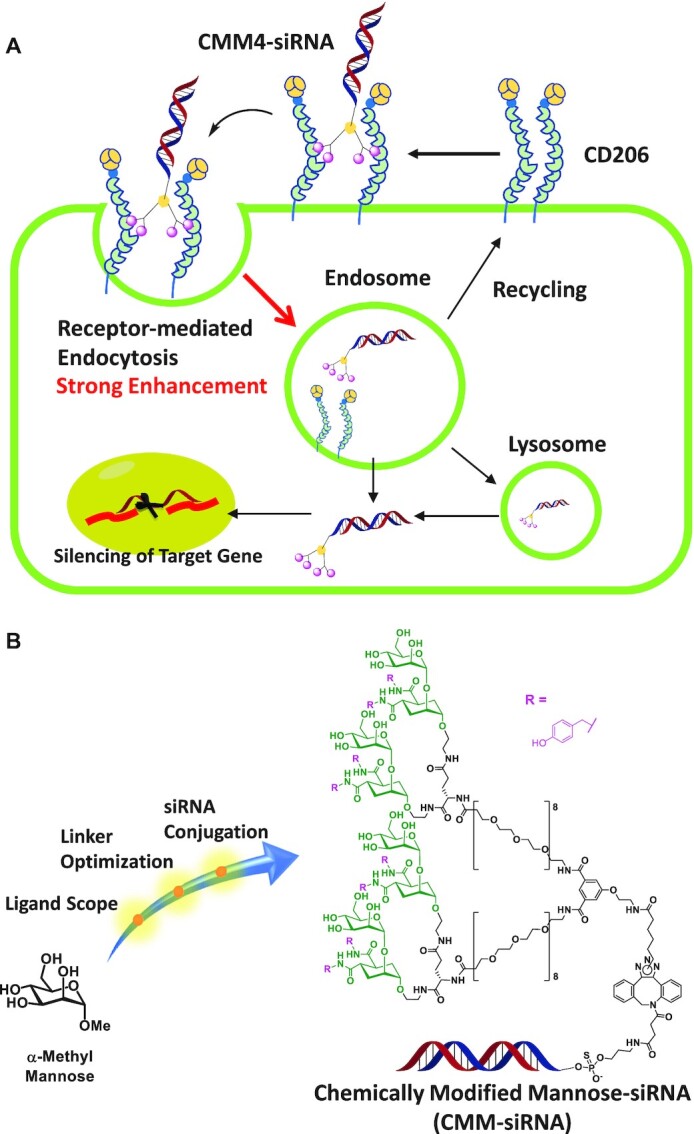
Schematic illustration of the delivery of siRNA and the synthetic strategy for CMM–siRNA. (**A**) The proposed schematic illustration of receptor-mediated targeted delivery of CMM–siRNA. (**B**) Strategy for obtaining high-affinity chemically modified mannose–siRNA conjugate (CMM–siRNA). The two approaches used were development of high-affinity mannose ligands and optimization of multivalent branched linkers.

Accordingly, in this study, we report a novel strategy for targeted delivery to macrophages and DCs using high-affinity CD206 ligand-conjugated siRNAs. To overcome the low binding affinity between the natural mannose ligand and CD206, we synthesized a chemically modified mannose ligand (CMM) to a branched linker, which was directly conjugated to an siRNA (Figure 1). This ligand-linker-conjugated siRNA (CMM–siRNA) exhibited strong and selective binding to CD206, resulting in selective silencing of CD206-expressing macrophages and DCs. Furthermore, we demonstrated the selectively targeted delivery and long-lasting gene silencing of CD206-expressing macrophages *in vivo*. This is the first study on systemic targeted delivery to macrophages and DCs using ligand-conjugated siRNA. These findings are expected to facilitate the development of novel targeting siRNA-based therapeutics for non-hepatic diseases.

## MATERIALS AND METHODS

### Materials

A series of oligonucleotides (terminal functional group-conjugated passenger, passenger, fluorophore-conjugated guide, and guide strands) were purchased from Gene Design or synthesized on an NS-8 Synthesizer (Gene Design) using commercially available phosphoramidite monomers via standard solid-phase oligonucleotide synthesis and deprotection protocols. The strand sequences mainly used are shown in Table 1.

**Table 1.** Oligonucleotide sequences

Oligonucleotide	Strand	Sequence (5′-3′)
siHPRT1	Passenger	fU^mC^fCmUfAmUfGmAfCmUfGmUfAmGfAmUfUmUfU^mA^fU
	Guide	pmA^fU^mAfAmAfAmUfCmUfAmCfAmGfUmCfAmUfAmGfGmA^fA^mU
siB2M(h)	Passenger	fA^mG^fGmAfCmUfGmGfUmCfUfUmUfCmUfAmUfCmU^fC^mU
	Guide	fA^mG^fAmGfAmUfAmGfAmAmAfGmAfCmCfAmGfUmCfCmU^fU^mG
siHPRT1-Alexa647	Passenger	fU^mC^fCmUfAmUfGmAfCmUfGmUfAmGfAmUfUmUfU^mA^fU
	Guide	pmA^fU^mAfAmAfAmUfCmUfAmCfAmGfUmCfAmUfAmGfGmA^fA^mU(**A647**)
C3N-siHPRT1	Passenger	fU^mC^fCmUfAmUfGmAfCmUfGmUfAmGfAmUfUmUfU^mA^fU(**C3N**)
	Guide	pmA^fU^mAfAmAfAmUfCmUfAmCfAmGfUmCfAmUfAmGfGmA^fA^mU
C3N-siB2M(h)	Passenger	fA^mG^fGmAfCmUfGmGfUmCfUfUmUfCmUfAmUfCmU^fC^mU(**C3N**)
	Guide	fA^mG^fAmGfAmUfAmGfAmAmAfGmAfCmCfAmGfUmCfCmU^fU^mG
C3N-siB2M(m)	Passenger	fA^mG^fGmAfCmUfGmGfUmCfUfUmUfCmUfAmUfAmU^fC^mU(**C3N**)
	Guide	fA^mG^fAmUfAmUfAmGfAmAmAfGmAfCmCfAmGfUmCfCmU^fU^mG
C3N-siHPRT1-Alexa647	Passenger	fU^mC^fCmUfAmUfGmAfCmUfGmUfAmGfAmUfUmUfU^mA^fU(**C3N**)
	Guide	pmA^fU^mAfAmAfAmUfCmUfAmCfAmGfUmCfAmUfAmGfGmA^fA^mU(**A647**)
CMM4–siHPRT1-a	Passenger	fU^mC^fCmUfAmUfGmAfCmUfGmUfAmGfAmUfUmUfU^mA^fU(**X**)
	Guide	pmA^fU^mAfAmAfAmUfCmUfAmCfAmGfUmCfAmUfAmGfGmA^fA^mU
CMM4–siHPRT1-b	Passenger	mU^mC^mCmUmAmUfGmAfCfUfGmUmAmGmAmUmUmUmU^mA^mU(**X**)
	Guide	pmA^fU^mAfAmAfAmUfCfUmAmCmAmGdTmCfAmUmAmGmGmA^mA^mU
CMM4–siHPRT1-c	Passenger	mU^mC^mCmUmAmUfGmAfCmUfGmUmAmGmAmUmUmUmU^mA^mU(**X**)
	Guide	pmA^fU^mAmAmAfAmUfCfUmAmCfAmGdTmCfAmUmAmGmGmA^mA^mU
CMM4-siB2M(h)	Passenger	fA^mG^fGmAfCmUfGmGfUmCfUfUmUfCmUfAmUfCmU^fC^mU(**X**)
	Guide	fA^mG^fAmGfAmUfAmGfAmAmAfGmAfCmCfAmGfUmCfCmU^fU^mG
CMM4-siB2M(m)	Passenger	fA^mG^fGmAfCmUfGmGfUmCfUfUmUfCmUfAmUfAmU^fC^mU(**X**)
	Guide	fA^mG^fAmUfAmUfAmGfAmAmAfGmAfCmCfAmGfUmCfCmU^fU^mG
CMM4-siCD45	Passenger	fU^mU^fCmUfGmGfCmUfGmAfAmUfUmUfCmAfGmAfG^mC^fA(**X**)
	Guide	pmU^fG^mCfUmCfUmGfAmAfAmUfUmCfAmGfCmCfAmGfAmA^fA^mA
CMM4–siHPRT1-a-Alexa488	Passenger	fU^mC^fCmUfAmUfGmAfCmUfGmUfAmGfAmUfUmUfU^mA^fU(**X**)
	Guide	pmA^fU^mAfAmAfAmUfCmUfAmCfAmGfUmCfAmUfAmGfGmA^fA^mU(**A488**)
CMM4–siHPRT1-a-Alexa647	Passenger	fU^mC^fCmUfAmUfGmAfCmUfGmUfAmGfAmUfUmUfU^mA^fU(**X**)
	Guide	pmA^fU^mAfAmAfAmUfCmUfAmCfAmGfUmCfAmUfAmGfGmA^fA^mU(**A647**)
CMM1-siHPRT1-a	Passenger	fU^mC^fCmUfAmUfGmAfCmUfGmUfAmGfAmUfUmUfU^mA^fU(**Y**)
	Guide	pmA^fU^mAfAmAfAmUfCmUfAmCfAmGfUmCfAmUfAmGfGmA^fA^mU
CMM1-siB2M(h)	Passenger	fA^mG^fGmAfCmUfGmGfUmCfUfUmUfCmUfAmUfCmU^fC^mU(**Y**)
	Guide	fA^mG^fAmGfAmUfAmGfAmAmAfGmAfCmCfAmGfUmCfCmU^fU^mG
CMM1-siHPRT1-a-Alexa488	Passenger	fU^mC^fCmUfAmUfGmAfCmUfGmUfAmGfAmUfUmUfU^mA^fU(**Y**)
	Guide	pmA^fU^mAfAmAfAmUfCmUfAmCfAmGfUmCfAmUfAmGfGmA^fA^mU(**A488**)
GalNAc4–siHPRT1-a	Passenger	fU^mC^fCmUfAmUfGmAfCmUfGmUfAmGfAmUfUmUfU^mA^fU(**Z**)
	Guide	pmA^fU^mAfAmAfAmUfCmUfAmCfAmGfUmCfAmUfAmGfGmA^fA^mU

mN and fN indicate 2′-*O*-methyl (2′-OMe) and 2′-deoxy-2′-fluoro (2′-F) sugar modifications, respectively, to adenosine (A), cytidine (C), guanosine (G) and uridine (U). dT indicates 2′-deoxythymidine. (X) and (Y) indicate chemically modified mannose ligand linker (CMM) and monovalent chemically modified mannose ligand linker (mono-CMM), respectively; (Z) indicates GalNAc ligand linker; (C3N) indicates C3-amine linker; (A488) indicates Alexa Fluor 488; (A647) indicates Alexa Fluor 647; ^ and p indicate phosphorothioate linkage and 5′ phosphate, respectively.

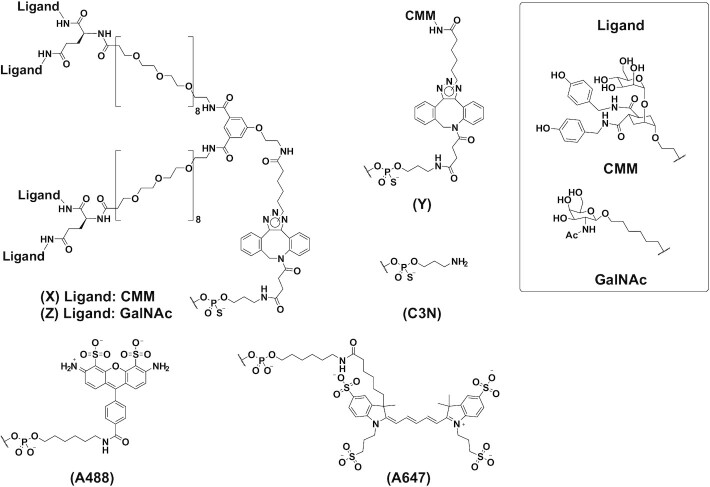

Chemically modified mannose ligand with conjugatable linkers (CMML1 and CMML4) were synthesized as described in the Supplementary Information. The two ligands (**4b**, **4c**) were synthesized as described previously (47). The GalNAc ligand with a conjugatable linker was synthesized using a modified version of a method described in the report (patent, WO/2017/131236).

### Conjugation

Reaction of C3-amine modified-sense strand (ss-Amine-C6-RNA) with dibenzocyclooctyne-*N*-hydroxysuccinimidyl ester (DBCO-NHS; Click Chemistry Tools [Scottsdale, AZ, USA]) in phosphate-buffered saline (PBS)/dimethyl sulfoxide (DMSO) at 25°C for 1.5 h provided DBCO-modified siRNA (ss-DBCO-RNA) and DBCO-NHS. The ss-DBCO-RNAs were separated and buffer-exchanged using a NAP-5 column (GE Healthcare UK Limited, Little Chalfont, UK) with 20 mM sodium acetate-150 mM NaCl buffer (pH 5.0). After concentration with an Amicon Ultra ultrafiltration device (MWCO: 3 kDa; GE Healthcare), the concentrations of ss-DBCO-C6-RNA were quantified by Dropsense (Trinean, Gentbrugge, Belgium). ss-DBCO-C_6_-RNA was then added to the CMML in sodium acetate buffer/DMSO at room temperature and left overnight. After checking the reaction by liquid chromatography (LC) with tandem mass spectrometry (MS) for completion, the reaction mixture was purified by preparative ion-pair reversed-phase high-performance LC with X-Bridge Prep (C18 5 μm, 10 × 100 mm; Waters [Milford, MA, USA]) with 0.1 M TEAA buffer/acetonitrile (MeCN). After concentration with an Amicon Ultra ultrafiltration device (MWCO: 3 kDa), the concentrations of CMM-conjugated sense strand were quantified by Dropsense. CMM–siRNAs were prepared from annealing of the CMM-conjugated sense strand with the corresponding antisense strand at 70°C for 10 min. After cooling to room temperature, the purity of the desired conjugate (CMM–siRNA) was determined by size exclusion chromatography with X-Bridge BEH200Å (7.8 × 300 mm, Waters) and PBS/MeCN (v/v = 3/7).

### Stability under serum conditions

For analysis of stability under serum conditions, 100 μM oligonucleotides in PBS was mixed with mouse serum at a 1:9 ratio, and each mixture was incubated at 37°C for a predetermined time. The siRNA or conjugate was analyzed by native-polyacrylamide gel electrophoresis (PAGE; FUJIFILM Wako Pure Chemical Corporation, Osaka, Japan) using SYBR Gold Nucleic Acid Gel Stain (Thermo Fisher Scientific, Waltham, MA, USA).

### Receptor binding

All experiments were performed using a Biacore T200 (GE Healthcare).

#### Method A

Man2-Biotin was chemically synthesized as shown in the Supporting Information and captured on the second flow cell of a Series S Sensor Chip SA (GE Healthcare). Injections were performed at 10 μl/min using HBS-EP+ (GE Healthcare) as running buffer. Next, 10 μg/ml His-tagged CD206 (R&D Systems, MN, USA) and each compound were co-added to the flow cells using HBS-P+ (GE Healthcare) supplemented with 5 mM CaCl_2_ as running buffer. The data were analyzed using Biacore Evaluation software program (GE Healthcare). The inhibition rate at each concentration was calculated by dividing each stability level by the signal without an inhibitor.

#### Method B

Penta-His antibody (Qiagen, Hilden, Germany) was diluted at a concentration of 50 μg/ml in 10 mM sodium acetate (pH 5) and immobilized on the surface of two flow cells of a Series S Sensor chip CM5 using an Amine Coupling Kit (GE Healthcare). Injections were performed at 10 μl/min using HBS-EP + as running buffer. The chip was equilibrated with HBS-P+ supplemented with 5 mM CaCl_2_, and the buffer was used as running buffer for subsequent experiments. His-tagged CD206 (R&D Systems) was captured on flow cell two at a concentration of 50 μg/ml at 10 μl/min for 210 s. Using a flow cell without CD206 as a control, the binding affinity of CMM4–siRNA to CD206 was measured. The flow rates of association and dissociation were both 30 μl/min. The chip surface was regenerated by adding 10 mM glycine–HCl (pH 1.5) for 30 s, followed by the addition of 3 M MgCl_2_ for 30 s. The concentration of the CMM4–siRNA increased from 0.39 to 200 nM. The equilibrium dissociation constant (*K*_D_) was obtained to evaluate binding affinity using Biacore Evaluation software (GE Healthcare).

### Preparation of human monocyte-derived macrophages and DCs

Two types of human monocyte-derived macrophages (h-Mo-Mφs) were generated as described in Supplementary Figure S1A. CD14^+^ monocytes (AllCells, Alameda, CA, USA) were cultured (37°C, 5% CO_2_) in RPMI1640 (Nacalai Tesque, Kyoto, Japan) supplemented with 10% fetal bovine serum (FBS) and 100 ng/ml human granulocyte macrophage colony-stimulating factor (GM-CSF; Miltenyi Biotec, Bergisch Gladbach, Germany) or 100 ng/ml human macrophage colony-stimulating factor (M-CSF; R&D Systems) for 7 days. The M-CSF-treated cells were further cultured in RPMI1640 supplemented with 10% FBS, 100 ng/ml human M-CSF, and 20 ng/ml human interleukin (IL)-4 (R&D Systems) for 2 days. Human monocyte-derived DCs (h-Mo-DCs) were obtained as shown in Supplementary Figure S2B. For h-Mo-DC generation, CD14^+^ monocytes were cultured in X-VIVO 15 medium (Lonza, Basel, Switzerland) supplemented with 100 ng/ml human GM-CSF and 100 ng/ml human IL-4. Cells were incubated at 37°C in an atmosphere containing 5% CO_2_ for 6 days, with medium changes on days 2 and 3. Generated cells were harvested on day 6 and reseeded in fresh medium supplemented with 100 ng/ml GM-CSF and 100 ng/ml IL-4. To generate mature DCs, cells were further supplemented with 10 μg/ml CD40 agonist antibody (patent, WO/2002/088186). The characteristics of monocyte-derived cells were verified by flow cytometry (FCM).

### FCM

For *in vitro* analysis, human cells were suspended in PBS supplemented with 0.02% ethylenediaminetetraacetic acid, 0.05% NaN_3_ and 1% bovine serum albumin. FcR blocking was performed using FcR Blocking Reagent, human (Miltenyi Biotec) for 30 min at 4°C. For staining of h-Mo-Mφ surfaces, cells were stained for 30 min at 4°C with phycoerythrin (PE)-conjugated anti-human CD206 (BD Biosciences, San Jose, CA, USA), Brilliant Violet 421 anti-mouse/human CD11b (Biolegend, San Diego, CA, USA), BV421 mouse anti-human CD163 (BD Biosciences), and fluorescein isothiocyanate anti-human β2-microglobulin antibodies (Biolegend). For staining of human monocyte-derived DCs, cells were stained for 30 min at 4°C with allophycocyanin (APC)-conjugated anti-human CD205 (DEC-205) antibodies (Biolegend), PE-conjugated anti-human CD206 antibodies (BD Biosciences), CD209 (DC-SIGN)-APC, human (Miltenyi Biotec), PE-conjugated anti-human CD11c antibodies (Biolegend), PE-conjugated human CD80 antibodies (BD Biosciences), BV421 mouse anti-human CD83 antibodies (BD Biosciences), and PE/Cy7-conjugated anti-human CD86 antibodies. FCM was performed using a BD FACSCanto II (BD Biosciences), BD LSRFortessa X-20 (BD Biosciences), and FlowJo software (BD Life Sciences–FlowJo, Ashland, OR, USA).

For *in vivo* analysis, splenic F4/80-positive cells were collected using Anti-F4/80 MicroBeads UltraPure Mouse and MS Columns (Miltenyi Biotec). Kidney cells were dissociated by treatment with collagenase (Sigma-Aldrich, St. Louis, MO, USA) and recombinant DNase I (Takara Bio, Shiga, Japan) for 60 min at 37°C. Subsequently, F4/80-positive cells were collected as described for splenic cells. Hepatic F4/80-positive cells were dissociated using a liver dissociation kit (mouse and gentleMACS Octo dissociator; Miltenyi Biotec). After Histodenz density gradient centrifugation, FcR blocking was performed using mouse FcR Blocking Reagent (Miltenyi Biotec) for 30 min at 4°C. For staining of cell surfaces, cells were stained for 30 min at 4°C with anti-CD206 (MMR) monoclonal antibodies (MR6F3) conjugated to PE-Cy7 (Thermo Fisher Scientific), APC/Cy7-conjugated anti-mouse F4/80 antibodies (Biolegend), and purified mouse anti-mouse β2 microglobulin (BD Biosciences) with True-Stain Monocyte Blocker (Biolegend). The antibody for β2 microglobulin was labeled with a Dylight 488 microscale antibody labeling kit (Thermo Fisher Scientific).

### RNA quantification

For *in vitro* analyses, cell lysates were prepared using TaqMan Gene Expression Cells-to-CT Kits (Thermo Fisher Scientific). The extracted RNA and standard Hprt-1 siRNA were converted to cDNA using a TaqMan MicroRNA RT Kit (Thermo Fisher Scientific) with the following primer: 5′-GTCGTATCCAGTGCAGGGTCCGAGGTATTCGCACTGGATACGACATTCCTATGAC-3′. Quantitative polymerase chain reaction (qPCR) was performed with TaqMan Gene Expression Master Mix and a QuantStudio 12K Flex Real-Time PCR System (Thermo Fisher Scientific) using the following primers: forward: 5′-CGCGCGCGATAAAATCTACAG-3′ and reverse: 5′-GTGCAGGGTCCGAGGT-3′ and TaqMan probe (5′-CTGGATACGACATTCC-3′). The primers and TaqMan probe were synthesized by Sigma-Aldrich and Thermo Fisher Scientific, respectively. For *in vivo* analysis, mouse serum was collected, and the amount of RNA was measured as described above.

### Cellular uptake study

All types of CD206-expressing cells prepared as described above were seeded into 96-well culture plates. AF488-labeled naked siRNA, GalNAc4-siRNA, CMM1–siRNA, or CMM4–siRNA was then added to each well at a concentration of 30 nM siRNA. After a 6 h incubation, the cells were isolated and washed with PBS three times. Trypan blue was added to the solution to quench the cell surface fluorescence. The uptake of conjugates in the cells was confirmed by FCM or RNA quantification.

### Fluorescence microscopy

M2a macrophages were seeded into 96-well plates and cultured in RPMI1640 medium supplemented with 10% FBS, 100 ng M-CSF, and 20 ng IL-4. AF488-labeled CMM4–siRNA or siRNA was added to each well at a concentration of 30 nM. As a negative control without compounds, Opti-MEM (Thermo Fisher Scientific) was added to each well. To observe the nuclei, Hoechst33342 was added to each well at a final concentration of 1 μg/ml. After a 1-h incubation, wells were observed using an In Cell Analyzer 6000 (GE Healthcare).

### Gene silencing

For the *in vitro* mRNA silencing assay, CD206-expressing cells were seeded in 96-well plates. Cells were then treated with appropriate compounds for 4 days, and cDNA was prepared with a SuperPrep Cell Lysis & RT Kit for qPCR (Toyobo, Osaka, Japan) according to the manufacturer's protocol. The transcript levels of *HPRT1* and *ACTB* were measured using TaqMan Gene Expression Master Mix (Thermo Fisher Scientific) and QuantStudio 12K flex (Thermo Fisher Scientific). TaqMan primer sets for human *HPRT1* (code no. Hs99999909_m1) and human *ACTB* (code no. Hs01060665_g1) were purchased from Thermo Fisher Scientific. The relative mRNA expression was quantified using the comparative Ct method. For the *B2M* gene, TaqMan primer sets for human *B2M* (code no. Hs00984230_m1) and human *GAPDH* (code no. Hs02786624_g1) were used. For *in vivo* evaluations, splenic and hepatic F4/80-positive cells were lysed using TRIzol Reagent (Thermo Fisher Scientific). The total RNA was then collected with a RNeasy Mini Kit (Qiagen) and converted to cDNA using a Transcriptor First Strand cDNA Synthesis Kit (Roche Life Science). qPCR was conducted as described above using TaqMan primer sets for mouse *Hprt1* (code no. Mm01545399_m1), mouse *B2M* (code no. Mm00437762_m1), and mouse *Actb* (code no. Mm00607939_s1).

### Animals

All animal studies were performed in accordance with Standards for Proper Conduct of Animal Experiments at Kyowa Kirin Co., Ltd., under the approval of the company's Institutional Animal Care and Use Committee. Kyowa Kirin Co., Ltd. is fully accredited by the Association for the Assessment and Accreditation of Laboratory Animal Care, International. Male C57BL/6JJcl mice (4 weeks old) were purchased from CREA Japan (Tokyo, Japan). Compounds were subcutaneously administered at 10 mg/kg. After 4 days, hepatic and splenic F4/80 cells were collected using anti-F4/80 beads (Anti-F4/80 MicroBeads UltraPure Mouse; Miltenyi Biotec) and MS columns (Miltenyi Biotec). The mRNA quantification in *Hprt1* was determined using the method described for the evaluation of gene silencing. For protein downregulation, the spleen and kidney were isolated, and B2M protein downregulation was determined by FCM and normalized to the geometrical mean of untreated mice.

### Pharmacokinetics and biodistribution

Compounds were subcutaneously administered at 1 mg/kg. Blood samples (50 μl) were collected from the tail vein at different times (0.5, 1, 4 and 24 h) using a BD Microtainer blood collection tube serum separator. Serum was obtained by centrifugation at 8000 rpm for 8 min. The RNA concentration was measured by stem–loop PCR as described above. For the biodistribution analysis, each compound was subcutaneously administered at 1 mg/kg. After 24 h, mice were euthanized, and the liver, lungs, kidneys, spleen, and heart were removed. The amount of sample retained in the tissue was normalized to the weight of the corresponding tissue. After lysate preparation, the amount of siRNA was quantified by stem–loop PCR. To assess *in vivo* selectivity at 24 h after subcutaneous injection, cells in the liver, spleen, and kidneys were suspended and analyzed by FCM.

### *In-situ* hybridization

Mouse spleens, livers, and kidneys were fixed with Gfix (Genostaff Co., Ltd., Tokyo, Japan), embedded in paraffin on CT-Pro20 (Genostaff Co., Ltd.) using G-Nox (Genostaff Co., Ltd.) as a less toxic organic solvent than xylene, and sectioned at a thickness of 6 μm. Tissue sections were deparaffinized with xylene and rehydrated through a series of ethanol washes and PBS. The sections were fixed with 4% paraformaldehyde in PBS for 15 min and then washed with PBS. Next, the sections were treated with 2 μg/ml ProteinaseK in PBS for 30 min at 37°C, washed with PBS, refixed with 4% paraformaldehyde in PBS, washed again with PBS, and incubated in 0.2 N HCl for 10 min. After more washing with PBS, the sections were acetylated by incubation in 0.1 M tri-ethanolamine-HCl (pH 8.0) and 0.25% acetic anhydride for 10 min. After more washing with PBS, hybridization was performed with digoxigenin-labeled LNA probe T(L)tcC(L)taT(L)gaC(L)tgT(L)agA(L)ttT(L)ta (GeneDesign, Osaka, Japan), which complementarily bound to the antisense strand of siRNA at concentrations of 15 nM in the G-Hybo L (Genostaff Co., Ltd.; RPD-02) at 50°C for 16 h. After hybridization, the sections were washed in 2 × G-Wash (SHW-01; Genostaff Co., Ltd.), equivalent to 2 × SSC, for 15 min and then in 50% formamide and 2 × G-Wash at 60°C for 20 min (three times). The sections were then washed twice with TBST (0.1% Tween20 in TBS). After treatment with G-block (GB-01; Genostaff Co., Ltd.) for 30 min, the sections were incubated with anti-DIG AP conjugate (Roche Diagnostics, Mannheim, Germany) and diluted 1:1000 with TBST for 2 h at room temperature. The sections were washed twice with TBST and then incubated in 100 mM NaCl, 50 mM MgCl_2_, 0.1% Tween20, and 100 mM Tris-HCl (pH 9.5).

Coloring reactions were performed with NBT/BCIP solution (Sigma-Aldrich), and sections were then washed with PBS, counterstained with Kernechtrot stain solution (Muto Pure Chemicals, Tokyo, Japan), dehydrated, and mounted with G-Mount (Genostaff Co., Ltd.).

### Statistical analyses

Statistical analyses were performed using the SAS software program, version 9.4 (SAS Institute Inc., Cary, NC, USA). Results are presented as means ± standard deviations, and statistical tests are indicated in the figures. To assess differences among all treated groups, one-way analysis of variance and Tukey's tests were used. Results with *P* values less than 0.05 were considered statistically significant.

## RESULTS

### Synthesis and binding properties of CMM

**Figure 2.**
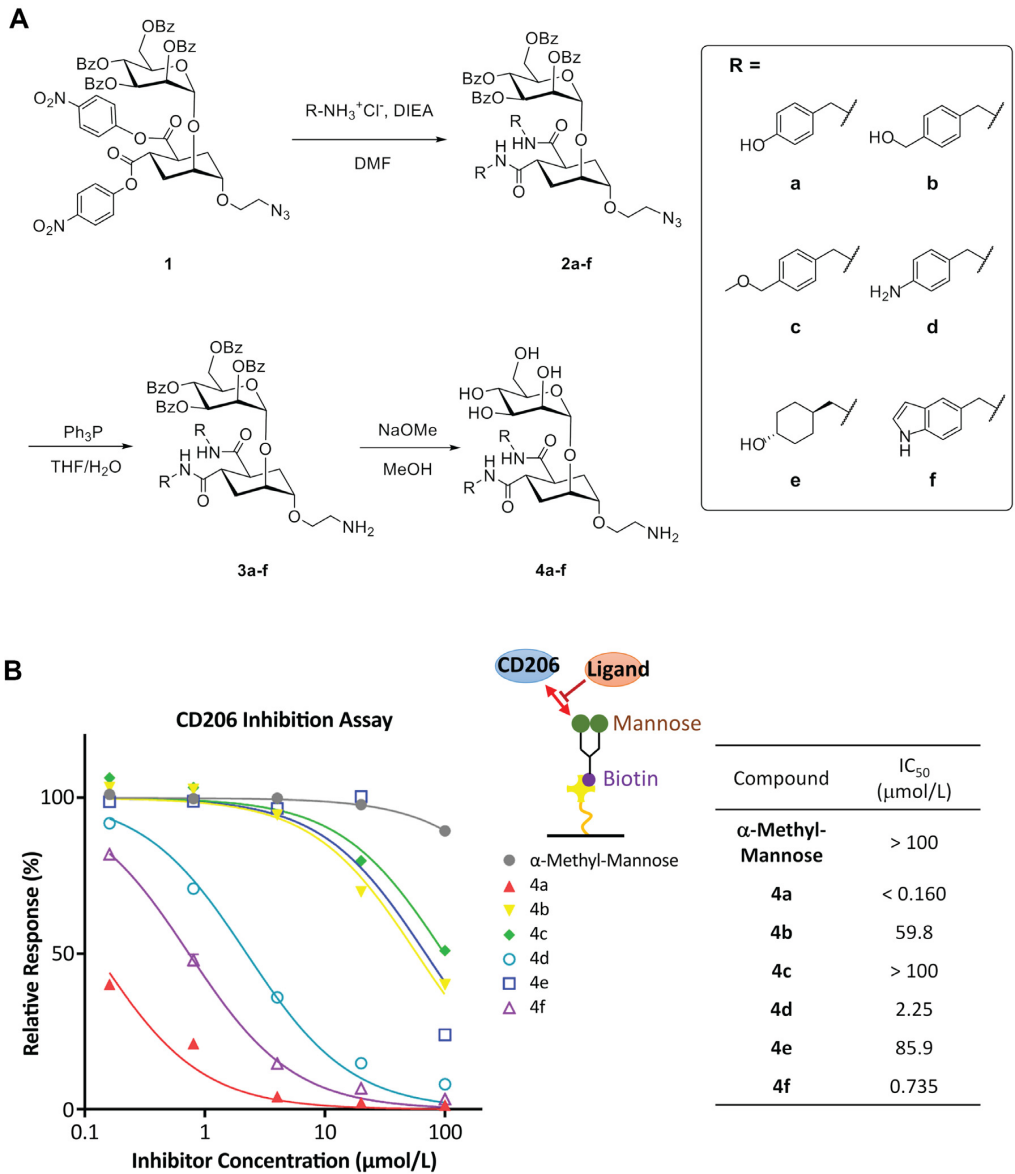
Synthetic scheme of chemically modified mannose ligands (CMMs) and their binding properties. (**A**) Synthesis of CMMs using a three-step reaction. (**B**) SPR results reflecting the inhibition of CD206 binding to the di-mannose surface by modified mannose compounds. Dose-response curves of percent activity were fit using a four-parameter logistic equation with XLfit software (ID Business Solutions, Guilford, UK), and IC_50_ values were calculated.

To construct the CD206L-siRNA conjugate, we first designed and synthesized CMM to a branched linker with a high affinity to CD206 (Figure 1). The synthetic route for the ligands is shown in Figure 2A. The conjugatable CMMs (**4a–f**) were obtained by a 3-step synthetic reaction: amidation of *para*-nitrophenyl ester **1** with alkyl or benzylamine derivatives (**2a–f**), reduction of the azide group, and deprotection of the benzoyl group on the mannose unit. The analytical data for the ligands are presented in detail in the Supporting Information (SI). The binding activity of ligands to di-mannose as inhibitors of CD206 was evaluated using surface plasmon resonance (SPR) analysis. Compact side chains with hydrogen donors, such as amino or hydroxyl group-substituted derivatives, showed high binding activity to CD206 (Figure 2B). The inhibitory activities of **4a** and **4f** were at least 1000 times higher than that of *α*-methyl mannose.

### Preparation and physicochemical characterization of CMM–siRNAs

**Figure 3.**
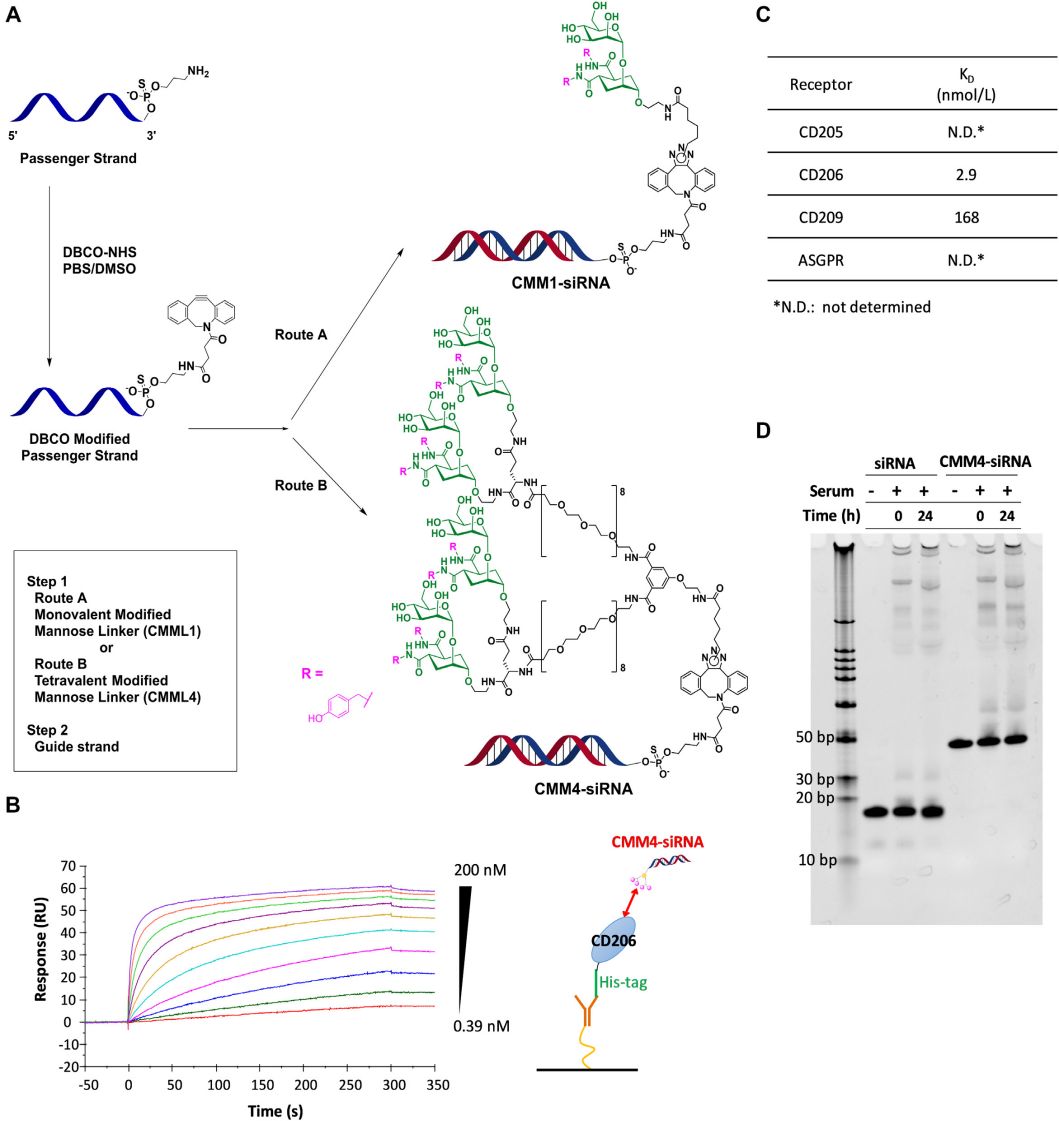
Preparation scheme and physicochemical characterization of CMM–siRNAs. (**A**) CMM–siRNAs were obtained using a three-step reaction. (**B**) SPR sensorgrams of the binding of CMM4–siRNA to CD206. (**C**) Binding affinities of modified mannose–siRNA to C-type lectins. (**D**) Native PAGE analysis of siRNA and CMM4–siRNAs in 90% mouse serum at 37°C.

Next, we synthesized two types of CMM–siRNAs, monovalent CMM–siRNA (CMM1–siRNA) and tetravalent CMM–siRNA (CMM4–siRNA). The synthetic scheme for these CMM–siRNAs is shown in Figure 3A. The synthetic scheme and method used for the generation of the ligand-linkers (CMML1 and CMML4) are shown in the Supplementary Information. The synthesized conjugates are listed in Table 1. The analytical data for the conjugates are described in detail in the Supplementary Information. The binding affinity between CMM4–siRNA and CD206 was evaluated by SPR analysis, and the dissociation constant (*K*_D_) was 2.9 nM (Figure 3B, C). To confirm the binding selectivity to the receptor, binding activities were evaluated using various sugar-binding receptors (ASGPR, CD205, CD209). The binding affinity to CD209, a mannose recognition receptor, showed moderate activity that was about 58 times lower than that between CMM4–siRNA and CD206. In contrast, this conjugate did not show any specific binding activities to ASGPR, a non-mannose-binding receptor. For the mannose-binding lectin CD205, the conjugate also did not show any specific binding activities. Thus, CMM4–siRNA exhibited high selectivity for CD206. We then evaluated the serum binding and stability in the presence of serum. Almost no binding was observed between CMM4–siRNA and serum proteins. In addition, we confirmed that the conjugate was stable in the presence of serum (Figure 3D).

### Cellular uptake and *in vitro* gene silencing

To verify the targeting effects of the CMM–siRNAs *in vitro*, we investigated the cellular uptake, gene silencing, and protein downregulation effects of CMM–siRNAs in CD206-positive cells. We used human monocyte-derived M1 and M2a macrophages and immature and mature DCs as CD206-positive cells (48,49). The expression of CD206 was observed in macrophages and DCs (Supplementary Figure S1).

**Figure 4.**
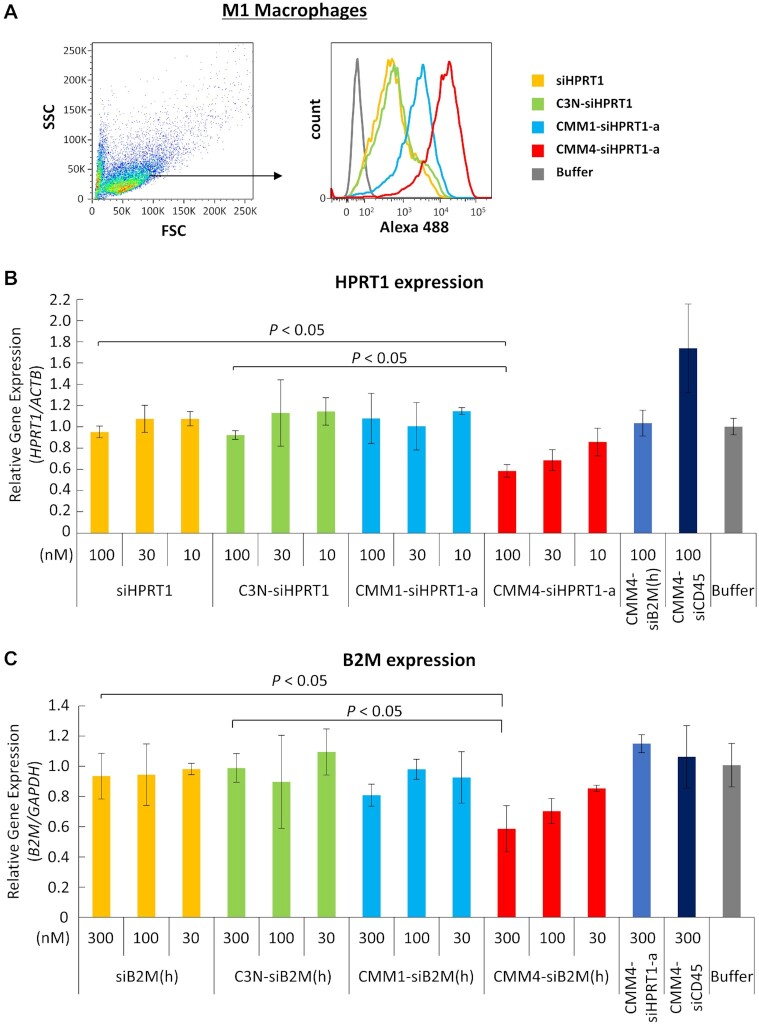
*In vitro* cellular uptake and gene silencing using CMM–siRNAs. (**A**) The cellular uptake in human monocyte-derived M1 macrophages. A488-labeled CMM1-siHPRT1-a, CMM4–siHPRT1-a, siHPRT1, and C3N-siHPRT1 were added to each cell type for 6 h. Cellular uptake was analyzed by flow cytometry. (**B**) Gene silencing of *HPRT1* in M1 macrophages. CMM1-siHPRT1-a, CMM4–siHPRT1-a, siHPRT1, C3N-siHPRT1, CMM4-siB2M(h), and CMM4-siCD45 were added to M1 macrophages for 4 days. *HPRT1* mRNA expression was determined using quantitative PCR and normalized to that of *ACTB* mRNA. (**C**) Gene silencing of *B2M* in M1 macrophages. CMM1-siB2M(h), CMM4-siB2M(h), siB2M(h), C3N-siB2M(h), CMM4–siHPRT1-a and CMM4-siCD45 were added to M1 macrophages for 4 days. *B2M* expression was determined using quantitative PCR and normalized to that of *GAPDH* mRNA.

In our initial experiments, we conducted the evaluation using M1 macrophages. Alexa Fluor 488 (A488)-labeled CMM4–siHPRT1-a was added to M1 macrophages at 30 nM. After incubation, the amount of A488-labeled CMM4–siHPRT1-a taken up was evaluated by FCM. The amount of CMM4–siHPRT1-a taken up by M1 macrophages was clearly increased compared with those of siHPRT1 and C3N-siHPRT1 as negative controls (Figure 4A and Supplementary Figure S2A). In parallel, we evaluated ligand valency-dependent uptake in M1 macrophages using CMM1-siHPRT1-a. The uptake amount was clearly dependent on ligand valency (Figure 4A; CMM1-siHPRT1-a versus CMM4–siHPRT1-a). Next, we investigated the gene silencing and protein downregulation effects in M1 macrophages. The siRNAs used in this study targeted hypoxanthine phosphor-ribosyltransferase 1 (*HPRT1*) and beta-2 microglobulin (*B2M*), which exhibit stable and high expression. CD45 target siRNA was used as a control target sequence to confirm target specificity in cells. CMM4–siHPRT1-a resulted in a strong enhancement of *HPRT1* gene silencing compared with siHPRT1 (Naked siRNA alone), C3N-siHPRT1 (ligand-unconjugated siRNA), CMM4-siB2M(h), and CMM4-siCD45 as negative controls (other targeted siRNAs; Figure 4B). In addition, we evaluated gene silencing using CMM4-siB2M(h) in M1 macrophages. Although enhancement of gene silencing was weaker than that of CMM4–siHPRT1-a, ligand-dependent enhancement of *B2M* gene silencing was observed (Figure 4C).

### *In vitro* protein downregulation

**Figure 5.**
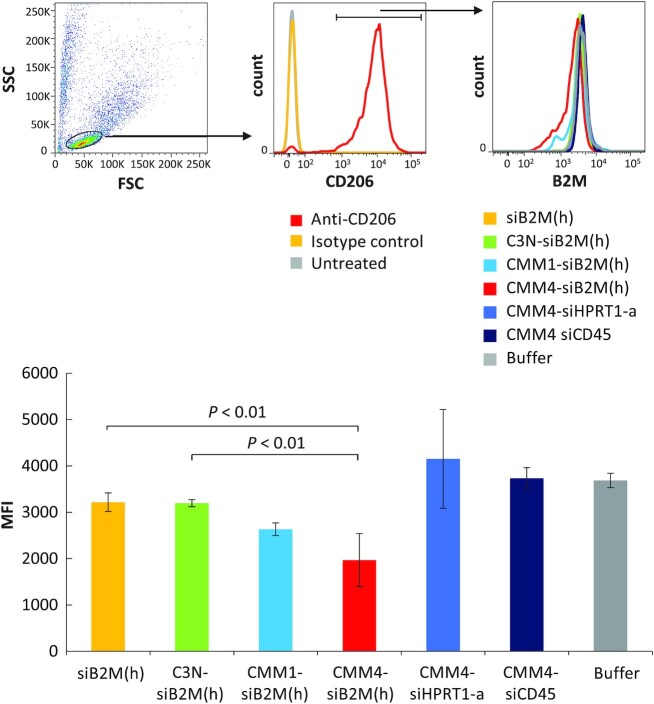
Protein downregulation in CD206-positive M1 macrophages. B2M protein downregulation in M1 macrophages, as analyzed by FCM. CMM1-siB2M(h), CMM4-siB2M(h), siB2M(h), C3N-siB2M(h), CMM4–siHPRT1-a and CMM4-siCD45 were added to M1 macrophages for 4 days and analyzed 3 days after medium exchange. The bar chart shows the means ± standard deviations of the geometric mean fluorescence intensity of triplicate experiments.

To confirm the downregulation of the target protein by silencing the target gene, we evaluated B2M protein expression using CMM1-siB2M(h) and CMM4-siB2M(h). B2M protein was expressed on the cell surface, which enabled protein downregulation measurement. To correctly confirm protein downregulation, we used several controls, including siB2M(h) (Naked siRNA), C3N-siB2M(h) (ligand unconjugated-siRNA), CMM-HPRT1-a and CMM-siCD45 (other targeted siRNAs). CMM4-siB2M(h) resulted in enhanced selective B2M protein downregulation compared with the controls (Figure 5). B2M protein downregulation efficiency was consistent with the observed gene silencing efficiency in M1 macrophages. Therefore, we confirmed that the CMM–siRNA showed ligand-dependent uptake, gene silencing, and protein downregulation effects in M1 macrophages.

### *In vitro* gene silencing in other CD206-expressing cells

**Figure 6.**
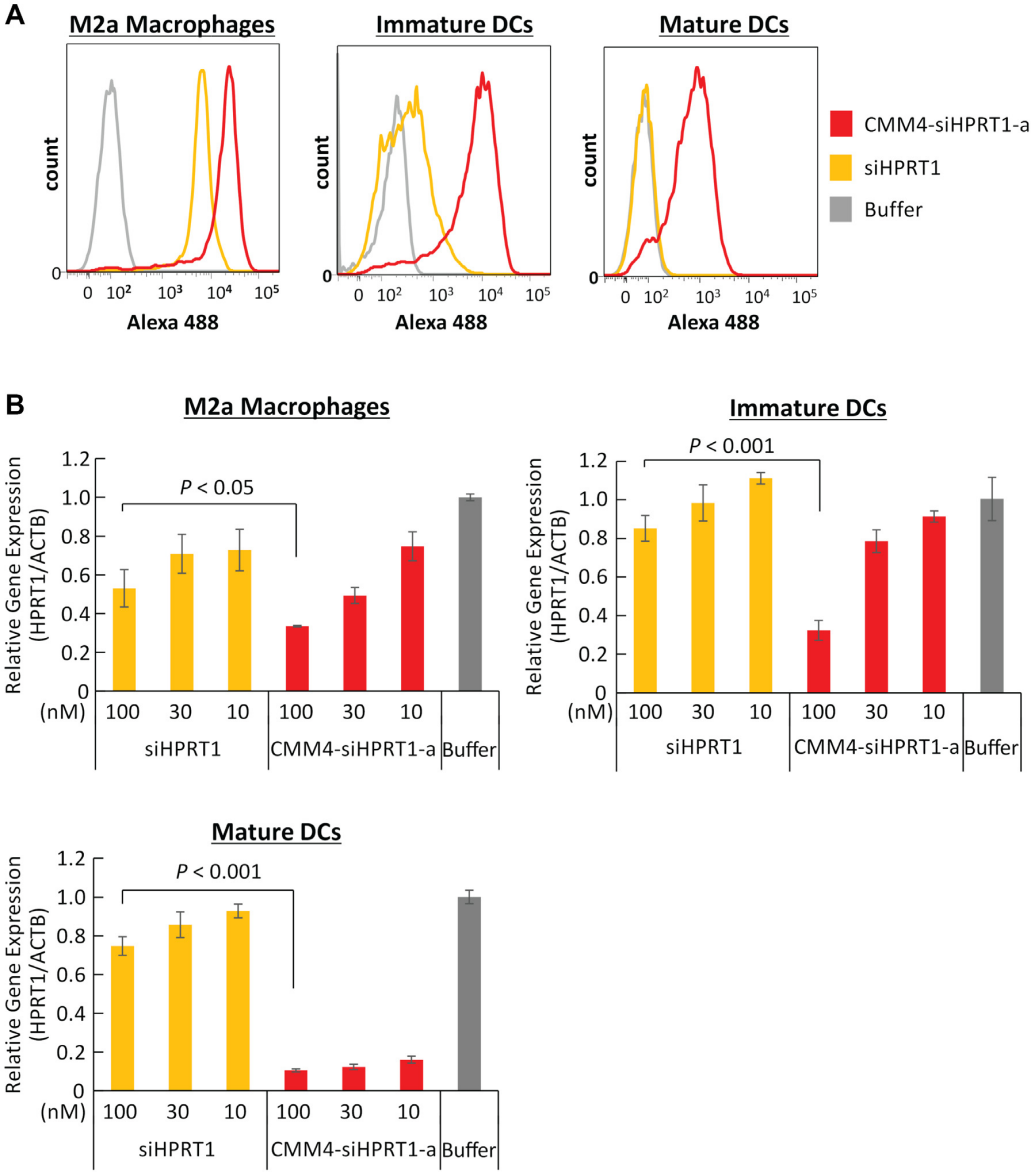
*In vitro* cellular uptake and gene silencing in CD206-positive M2a macrophages, immature DCs, and mature DCs using CMM4–siHPRT1. (**A**) Cellular uptake in human monocyte-derived M2a macrophages and immature and mature DCs. A488-labeled CMM4–siHPRT1-a and siHPRT1 were added to cells for 6 h. Cellular uptake was analyzed by flow cytometry. (**B**) Gene silencing activities in M2a macrophages, immature DCs, and mature DCs. CMM4–siHPRT1-a and siHPRT1 were added to cells for 4 days. *HPRT1* mRNA expression was determined using quantitative PCR and normalized to that of *ACTB* mRNA. The data represent means ± standard deviations of triplicate experiments.

To clarify whether the CMM4–siRNA showed similar effects in other CD206-expressing cells, we investigated cellular uptake and gene silencing effects in M2a macrophages and immature and mature DCs using CMM4–siHPRT1-a. In the cellular uptake assay, the amounts of CMM4–siHPRT1-a taken up by M2a macrophages and DCs were clearly increased compared with that of siHPRT1 as a negative control (Figure 6A). siRNA uptake was quantified using stem–loop reverse transcription (RT) qPCR. These results were closely correlated with those of FCM (Supplementary Figure S2). In addition, we performed fluorescent imaging analysis (Supplementary Figure S3), which demonstrated that CMM4–siHPRT1-a exhibited at least three-fold stronger gene silencing than siHPRT1 in M2a macrophages and DCs (Figure 6B). In particular, extremely strong enhancement of gene silencing (more than 10 times stronger than that of siRNA) was observed in mature DCs. To confirm the ligand effect, we evaluated gene silencing using cells lacking CD206 expression, such as hepatocytes. Although GalNAc–siRNA (GalNAc4–siHPRT1-a) showed strong gene silencing (more than 80% silencing at 10 nM), there was little suppression of *Hprt1* expression in the spleen or liver when using CMM–siRNA and siRNA (Supplementary Figure S4).

Moreover, we compared the *in vitro* uptake and gene silencing effects of CMM4–siRNA and siRNA as non-CD206-binding controls. The half-maximal inhibitory concentration [IC_50_] of gene silencing activities of CMM4–siRNA on M1 and M2a macrophages were approximately 100 and 30 nM, respectively (Figures 4B and 6B), whereas uptake rates by M1 and M2a macrophages, as quantified by stem–loop PCR at 4 h, were 259 000 and 874 000 copies/cells, respectively (Supplementary Figure S2). Moreover, when comparing the uptake and gene silencing activities of immature and mature DCs, gene silencing on mature DCs (IC_50_ < 10 nM; Figure 6B) was higher than that on immature DCs (IC_50_ = 30–100 nM; Figure 6B), whereas the uptake of conjugates by immature DCs (1 260 000 copies/cell; Supplementary Figure S2) was higher than that by mature DCs (647 000 copies/cell; Supplementary Figure S2). These results indicated that the uptake of conjugates was not always consistent with gene silencing activity.

Furthermore, because DCs express various lectins, including CD205, CD206, and CD209 (Supplementary Figure S1), we then investigated the principal receptors mediating the uptake of CMM4–siRNA. After downregulating each receptor by transfection with specific siRNAs (Supplementary Figure S5A), CMM4–siHPRT1-a was added to cells, and uptake was evaluated. In cases of CD205 or CD209 downregulation, the uptake of conjugates was unchanged compared with that before downregulation; by contrast, in cases of CD206 downregulation, uptake was reduced compared with that before downregulation. These findings indicate that the principal receptor for uptake by DCs was CD206 (Supplementary Figure S5B).

### *In vivo* distribution

**Figure 7.**
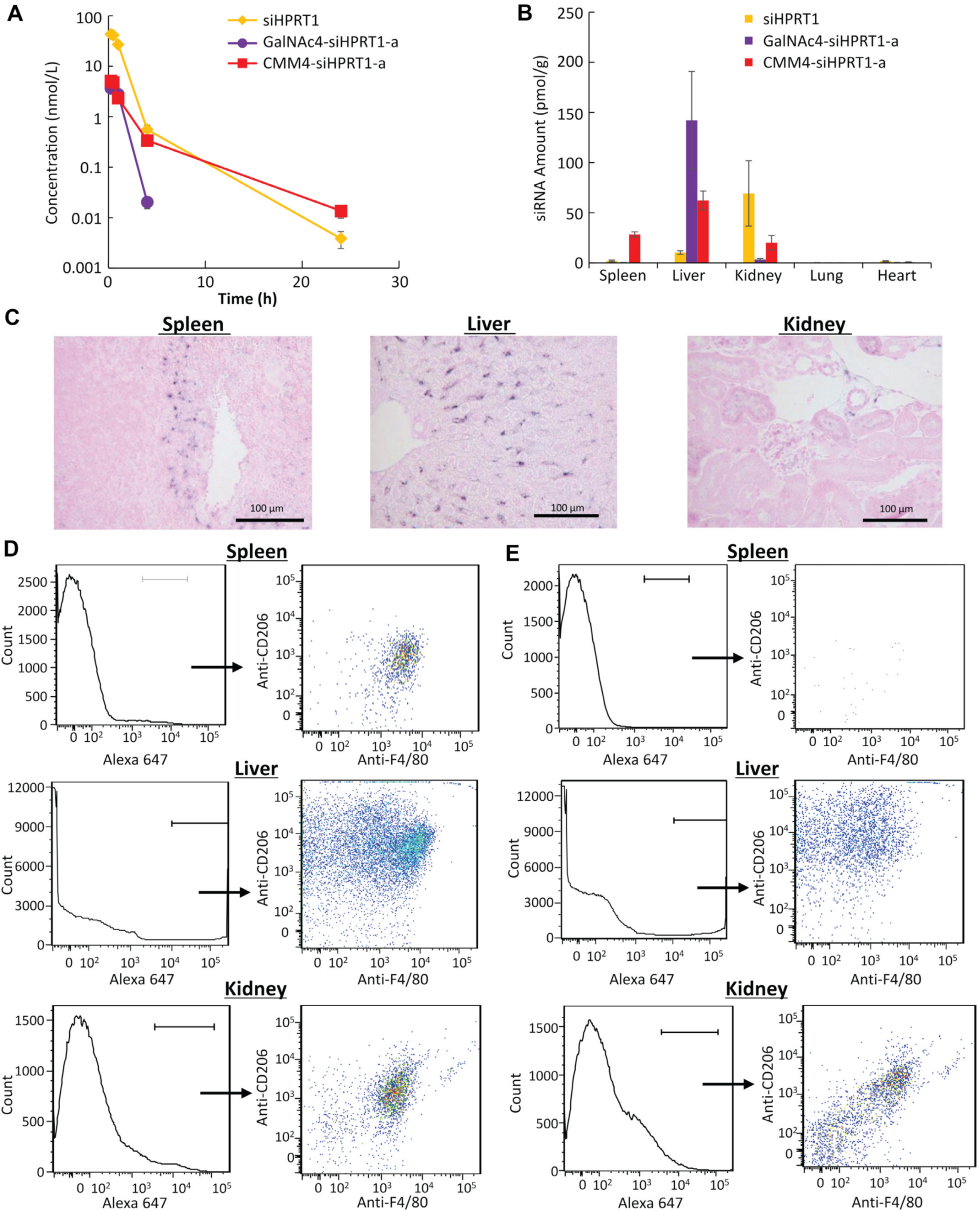
*In vivo* biodistribution and targeting ability. (**A**) Blood circulation properties of CMM4–siHPRT1-a, GalNAc4–siHPRT1-a, and siHPRT1 after a single subcutaneous injection (1 mg/kg) into C57BL6/J mice. (**B**) Biodistributions of siHPRT1, GalNAc4–siHPRT1-a, and CMM4–siHPRT1-a conjugates 24 h after a single subcutaneous injection. After dissociation of the cells from each organ, the amount of siRNA was quantified by stem–loop PCR. The values represent means ± standard deviations of triplicate experiments. (**C**) *In situ* hybridization of CMM4–siHPRT1-a in the spleen and liver 24 h after a single subcutaneous injection. Blue signal indicates the localization of modified mannose–siRNA. The boxed areas in the upper panels are magnified in the lower panels. (**D**) Cell-targeting profiles of A647-labeled CMM4–siHPRT1-a. (**E**) Cell-targeting profiles of A647-labeled siHPRT1. The population of compounds was determined by FCM using antibodies targeting F4/80 and CD206 markers.

Next, we evaluated the blood circulation properties of CMM4–siRNA for targeting CD206 cells in C57BL6/J mice administered a single subcutaneous injection of siHPRT1, GalNAc4–siHPRT1-a, or CMM4–siHPRT1-a. Quantification using RT-qPCR showed that these compounds were rapidly eliminated from the blood, regardless of the presence of the targeting moiety (Figure 7A).

The tissue distribution of each compound was then examined 24 h after injection. Although GalNAc4–siHPRT1-a and siHPRT1 accumulated in the liver and kidneys, respectively, CMM4–siHPRT1-a accumulated in the liver, spleen, and kidneys (Figure 7B). In addition, CMM4–siHPRT1-a accumulation was examined in the spleen and liver 24 h after injection by *in situ* hybridization (Figure 7C), indicating its tendency to accumulate in some cells of these three organs. To further clarify cell targeting profiles in the spleen, liver, and kidneys, Alexa Fluor 647 (A647)-labeled CMM4–siHPRT1-a was injected into mice. The results showed that it mainly accumulated in F4/80- and CD206-positive cells (Figure 7D). By contrast, A647-labeled siHPRT-1, as a non-ligand-conjugated siRNA, showed less accumulation and selectivity for CD206-positive cells than CMM4–siHPRT1 (Figure 7E). Our results thus indicated that the CMM4–siRNA conjugate selectively accumulated in CD206-positive cells.

### *In vivo* gene silencing

**Figure 8.**
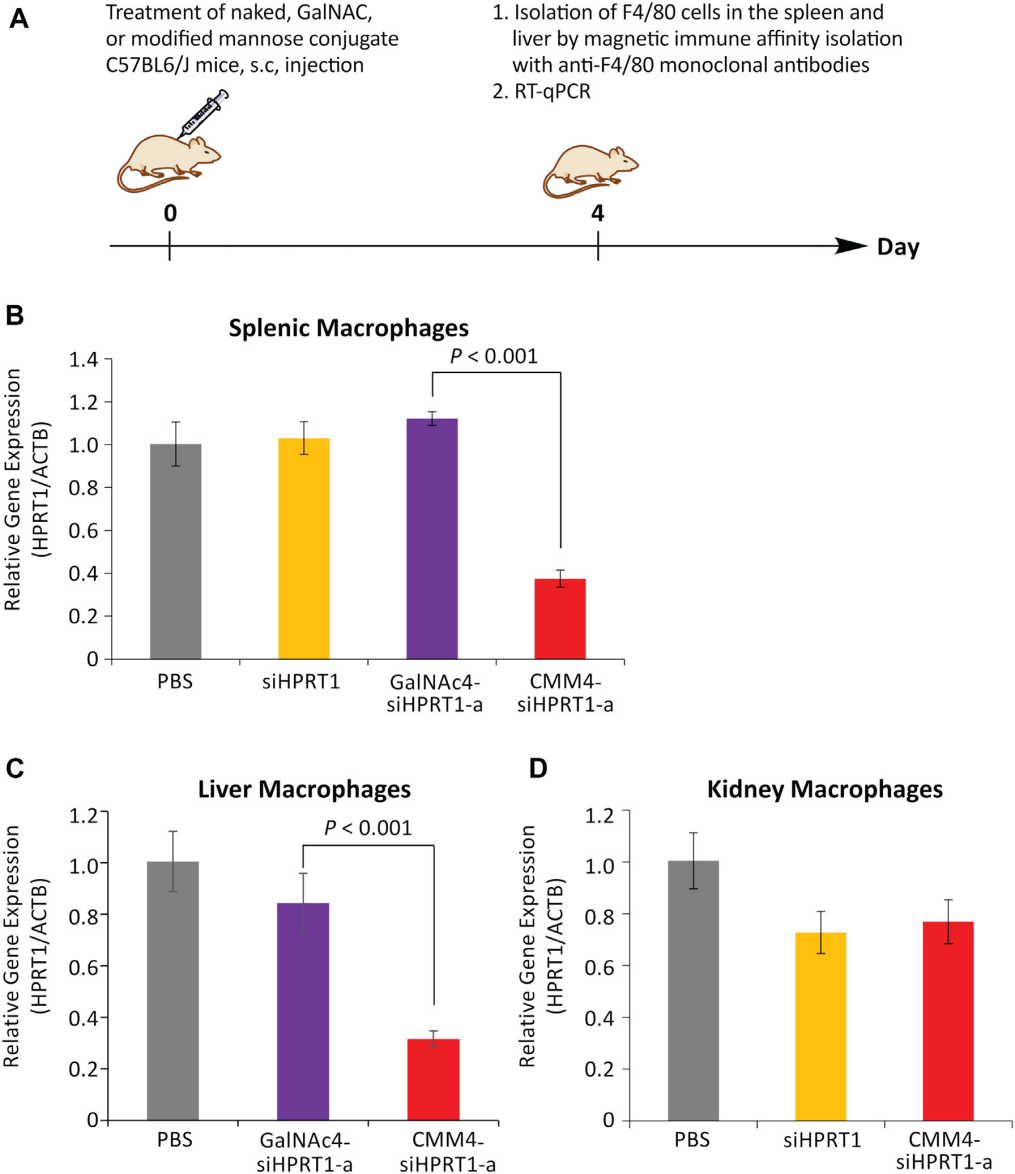
Gene silencing in CD206-positive macrophages in the spleen, liver, and kidneys of mice treated with CMM4–siHPRT1-a *in vivo*. (**A**) Schematic illustration of gene silencing. The gene silencing activities of oligonucleotides on splenic macrophages (**B**) and hepatic F4/80-positive macrophages (**C**) and kidney F4/80-positive macrophages (**D**). *Hprt1* mRNA expression was quantified by RT-qPCR and normalized to that of *ACTB* mRNA.

Next, the gene silencing effects of CMM4–siRNA on target genes *in vivo* were evaluated in mice. First, as shown in Figure 8A, after subcutaneous injection, F4/80-positive macrophages in the spleen, liver, and kidneys were isolated using anti-F4/80 antibody-loaded magnetic beads. The purity of the F4/80-positive cell population in the spleen, liver, and kidneys, as analyzed by FCM using antibodies to F4/80 and CD206 markers, was determined (Supplementary Figure S6).

The expression of *Hprt1* mRNA was then measured by RT-qPCR, and the results were normalized to the expression of the housekeeping gene actin beta (*ACTB*). CMM4–siHPRT1-a (10 mg/kg) strongly enhanced gene silencing in F4/80-positive cells in the spleen and liver compared with siRNA or GalNAc4–siHPRT1-a (Figure 8B, C; CMM4–siHPRT1-a: >60% gene silencing, siHPRT1 or GalNAc4–siHPRT1-a: <20% gene silencing). By contrast, the gene silencing activity of CMM4–siHPRT1-a in CD206-positive kidney cells was the same as the observed activity using siRNA (Figure 8D).

**Figure 9.**
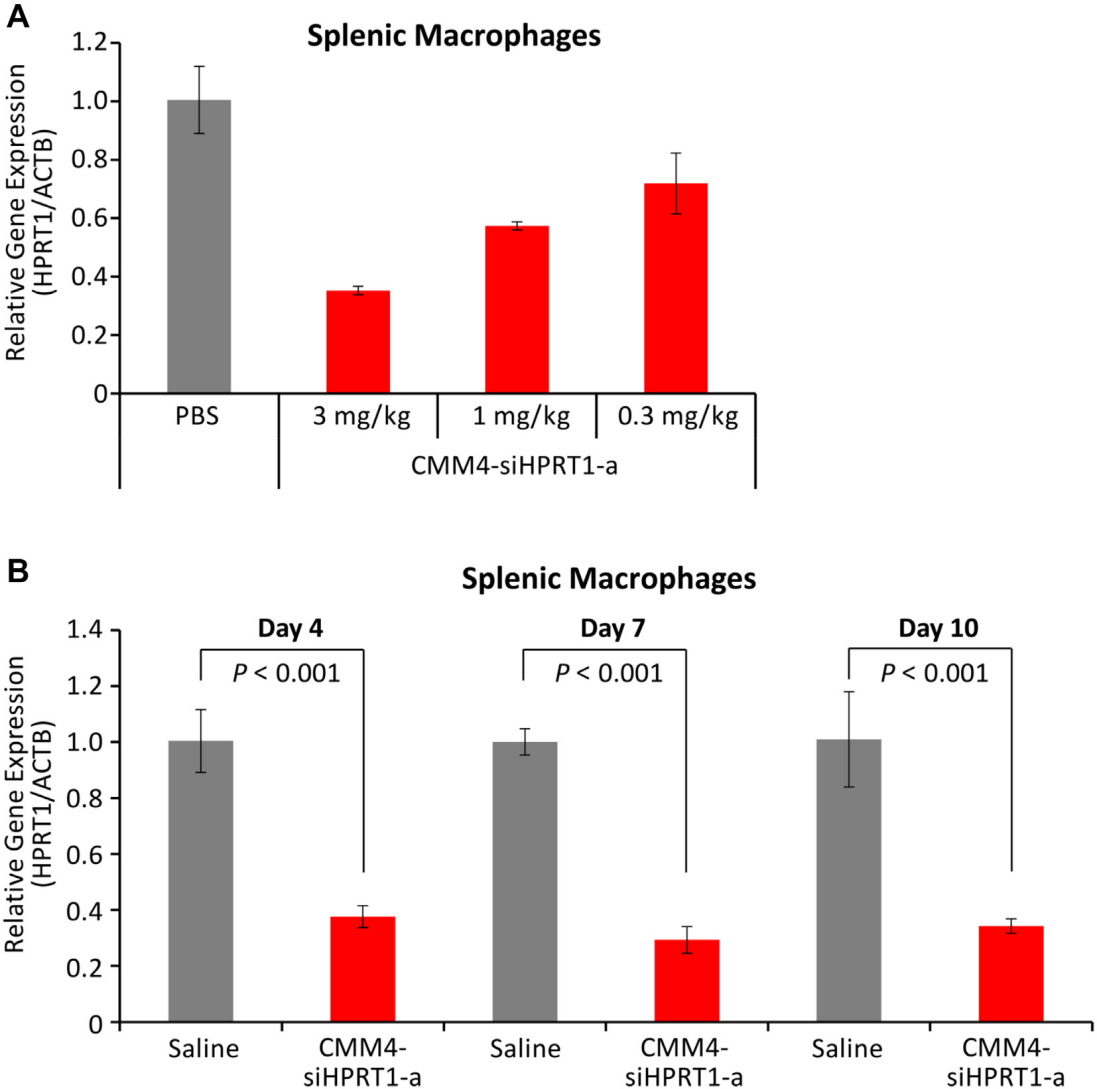
Dose-dependency and persistence of gene silencing. (**A**) Dose-dependent gene silencing of splenic F4/80-positive macrophages. (**B**) Continuous gene silencing of the target mRNA (*Hprt1*) with CMM4–siHPRT1-a. CMM4–siHPRT1-a was administered to mice subcutaneously (3 mg/kg, single dose). After 4, 7, and 10 days, the target mRNA in splenic F4/80-positive cells was evaluated using RT-qPCR.

To confirm the cell selectivity, we evaluated gene silencing effects in whole spleens and livers using each conjugate. Although strong gene silencing was observed in the liver when using GalNAc, no or little suppression of the target gene was observed in the spleen and liver when using CMM4–siHPRT1-a (Supplementary Figure S7). The gene-suppressing efficiency was consistent with accumulation in the spleen and liver (Figure 7). Next, to further clarify the properties of this conjugate, we evaluated the dose-dependent effects of CMM4–siHPRT1-a and continuous suppression of the *Hprt1* gene using this conjugate. We confirmed the suppression of the target gene was almost linear at doses of 0.3 to 3 mg/kg. At a dose of 1 mg/kg, CMM4–siHPRT1-a suppressed target gene expression by 43% (Figure 9A). The gene silencing observed in F4/80-positive cells in the spleen persisted for at least 10 days (Figure 9B).

**Figure 10.**
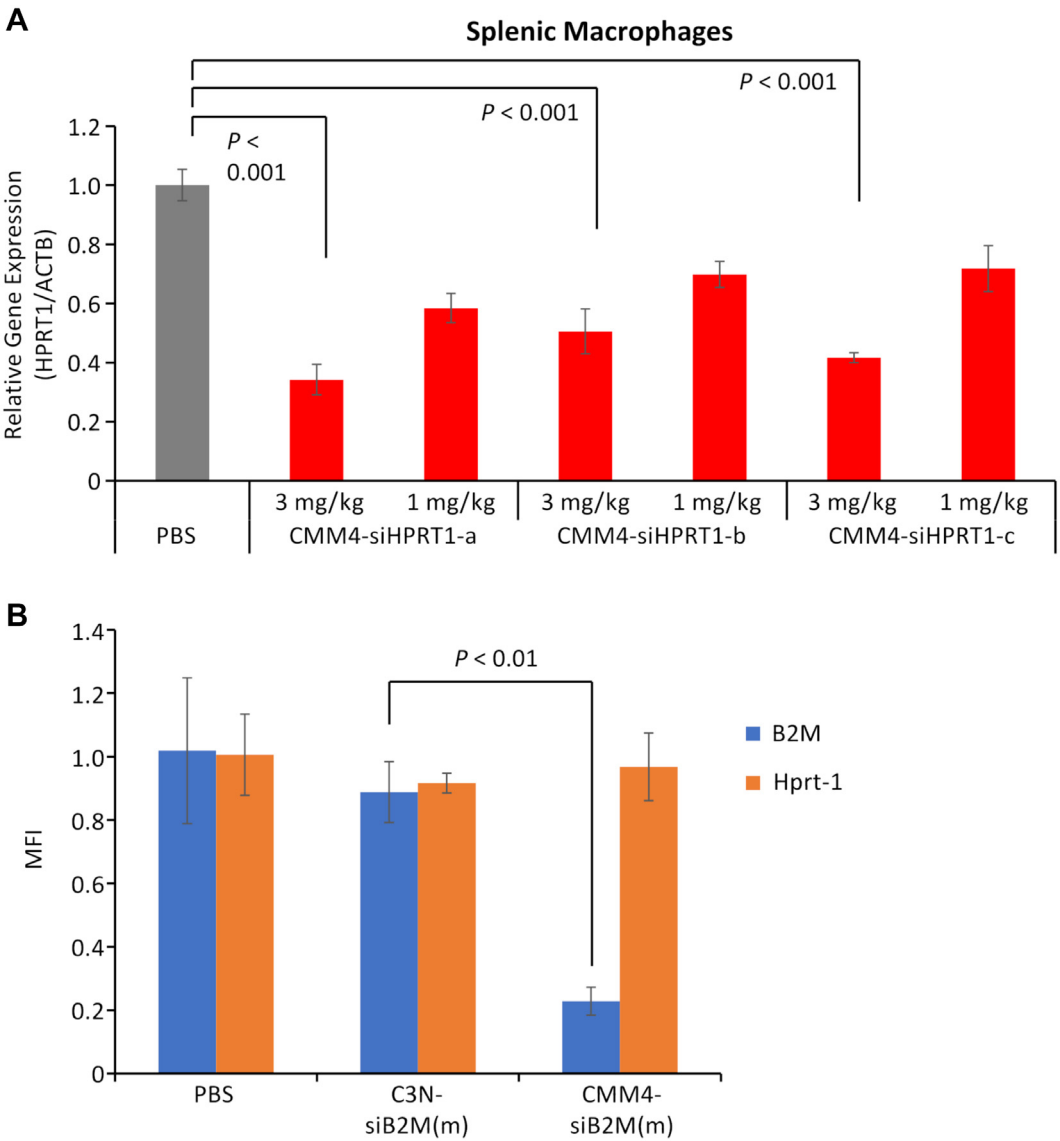
Effects of the conjugates on gene silencing. Gene silencing in splenic F4/80-positive macrophages was analyzed by RT-qPCR. (**A**) Four days after subcutaneous administration of CMM4–siRNAs (CMM4-HPRT1-a, CMM4–siHPRT1-b, CMM4–siHPRT1-c) and (**B**) 10 days after subcutaneous administration of CMM4-siB2M(h) and C3N-siB2M(h).

Furthermore, when we switched to another siRNA sequence for the *Hprt1* gene, CMM4–siHPRT1-b and CMM4–siHPRT1-c showed similar gene silencing effects (Figure 10A). Similar to our evaluation of *in vitro* gene silencing, we conducted an evaluation of *in vivo* gene silencing in splenic macrophages using CMM4-siB2M(m). We observed strong and selective suppression of the *B2M* gene, similar to *Hprt1* (Figure 10B).

### *In vivo* protein downregulation

**Figure 11.**
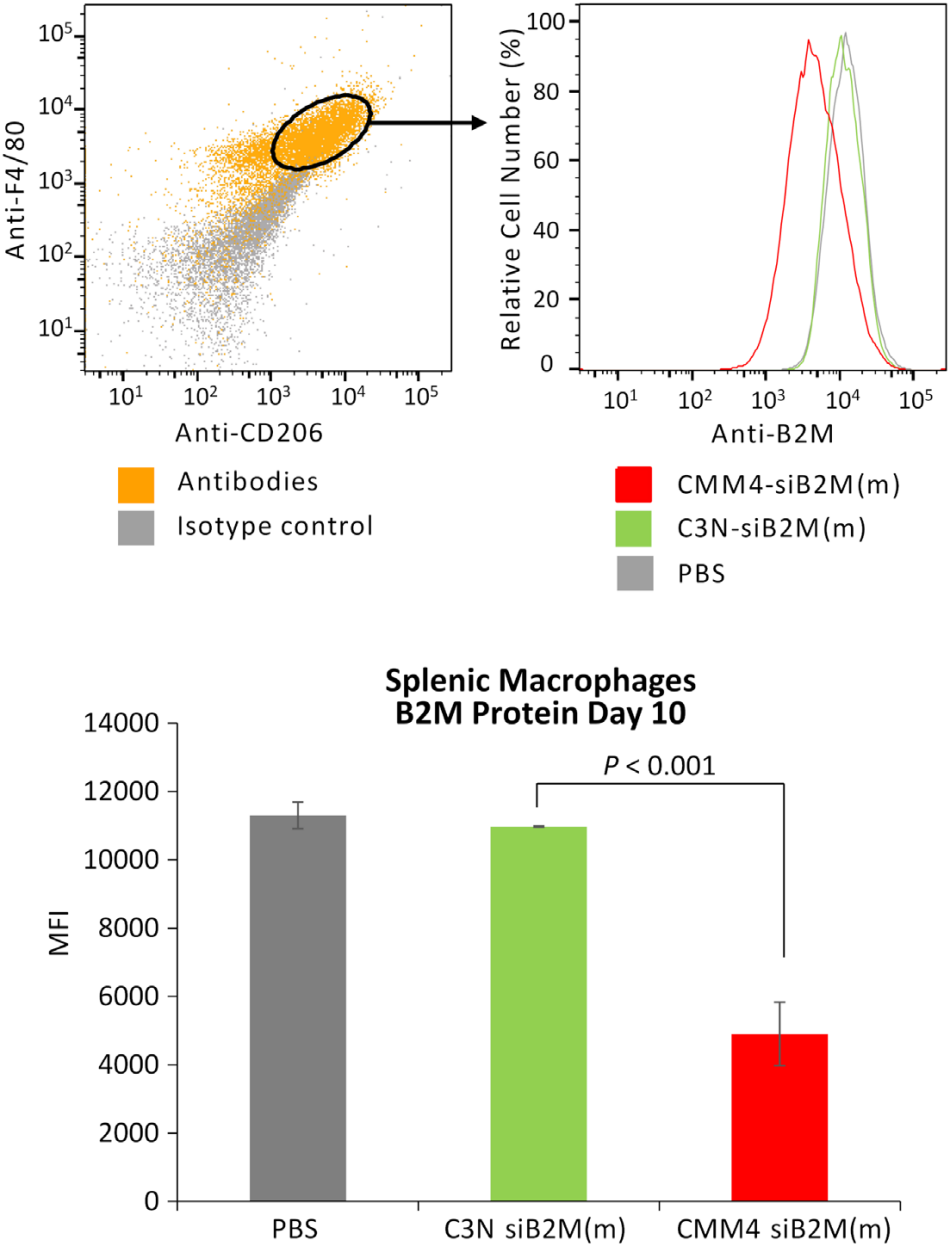
Protein downregulation in splenic CD206/F480-positive macrophages. The amount of target protein (B2M protein) in individual animals was quantified by FCM. Values represent means ± standard deviations of the geometric mean fluorescence intensity of triplicate experiments.

**Figure 12.**
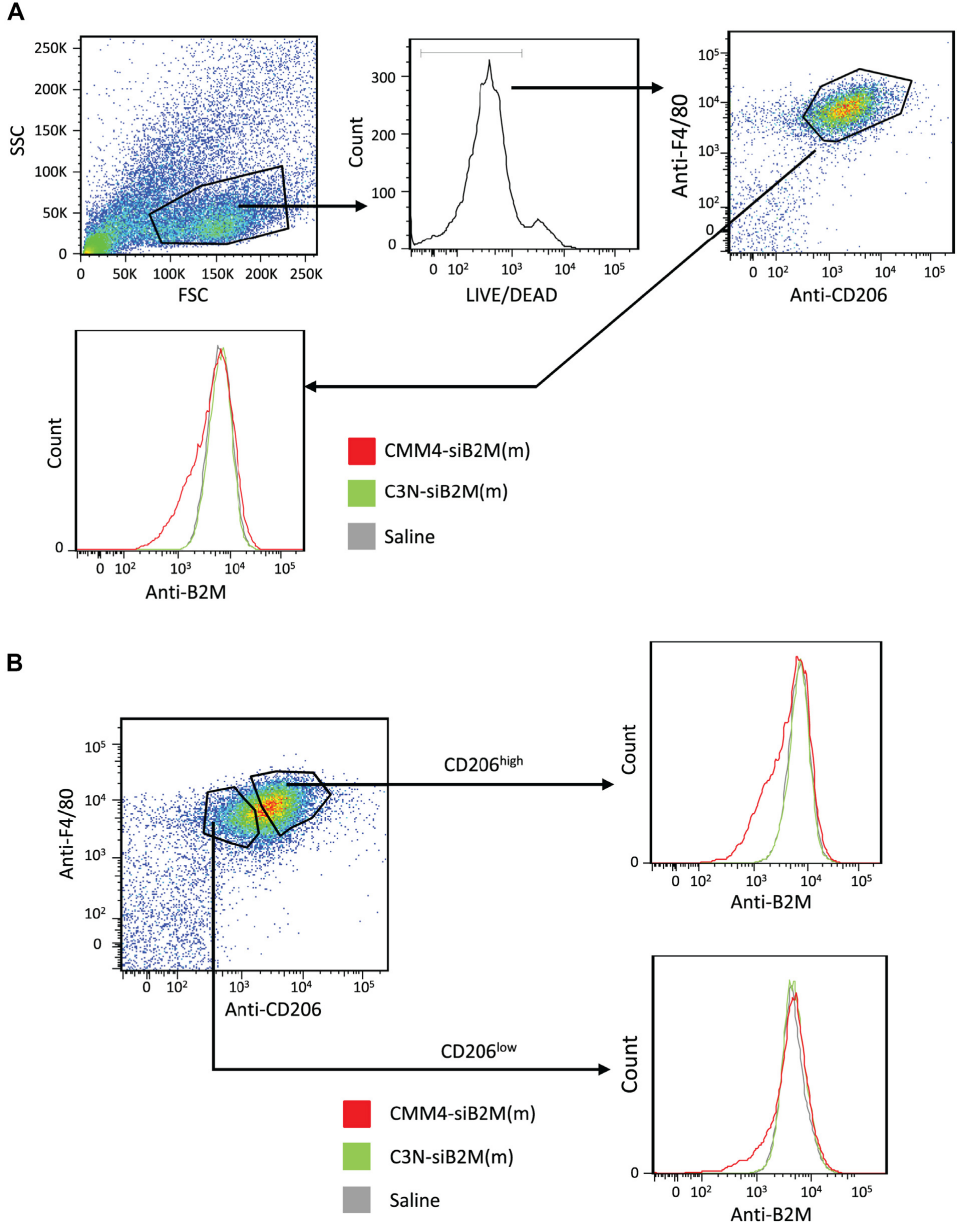
Downregulation in protein levels in mouse kidneys treated with CMM4-siB2M(m) *in vivo*. (**A**) The amount of target protein (B2M protein) in individual animals was quantified by FCM. (**B**) Analysis of downregulation of the target protein (B2M protein) in CD206-high/low populations.

Protein downregulation by CMM4-siB2M(m) was also evaluated *in vivo*, as shown in Figures 11 and 12. In the spleen, B2M protein levels, as measured by FCM in both F4/80 and CD206-positive cell regions, were strongly reduced by CMM4-siB2M(m) (Figure 11). Contrastingly, no marked downregulation in B2M expression was observed using C3N-siB2M(m) as a CMM-unconjugated siRNA. Next, we evaluated protein downregulation in kidney macrophages using CMM-siB2M(m) (Figure 12). To clarify whether CMM4–siRNA exhibited a CD206-dependent effect (owing to receptor-mediated gene silencing), we analyzed B2M protein downregulation using CMM4-siB2M(m) (Figure 12A). B2M protein downregulation was dependent on CD206 expression and was higher in CD206^high^ cells than in CD206^low^ cells (Figure 12B). Therefore, these findings confirmed that systemic administration of CMM4–siRNA showed *in vivo* targeted delivery and downregulated the target gene and protein in CD206-expressing cells.

## DISCUSSION

Systemically administered siRNAs that exhibit selective targeting with strong gene-silencing activities for extrahepatic cells are highly desirable because they have great potential utility for creating druggable targets from undruggable ones. Here, we demonstrated a novel strategy for the targeted delivery of siRNA to macrophages and DCs by conjugating siRNAs to chemically modified mannose ligands. The conjugate generated in this study exhibited strong and selective binding to CD206 followed by subsequent silencing of CD206-expressing cells *in vivo*.

Targeting CD206 using a mannose ligand may enable accumulation of target molecules in the cells owing to high CD206 recycling rates (<5 min) and intracellular binding mechanisms (40), similar to GalNAc–siRNA, which is a pharmaceutically acceptable construct (13,14). However, it is difficult to construct paucivalent mannose ligand-conjugated siRNAs, such as GalNAc–siRNA, because of the low affinity of mannose to CD206 (42–44). Therefore, very high multivalent scaffolds, such as polymers and nanoparticles, were usually used in previous studies (50,51). In this study, we found that chemical modification of the mannose ligand-conjugated tetravalent linker construct exhibited high binding activity to CD206. Although examination of a high-affinity mannose ligand for CD206 has not yet been reported (52), we found that the high-affinity ligand for CD209 developed by Bernaldi's group (47,53) had high binding activity for CD206. In addition, a structure-activity relationship (SAR) study of CD206 using a series of mannose derivatives revealed great enhancement of the binding affinity toward natural mannose ligand (at least 1000 times higher than the natural mannose ligand) and a different SAR compared with CD209 (47). Multivalent construction of a glycan ligand using a linker can enhance the binding activity of the ligand to a receptor. Thus, when designing a paucivalent ligand construct, it is important to modify the design based on the receptor structure (46). Based on information concerning the interactions of several types of glycan clusters with the carbohydrate recognition domain (54,55), our study demonstrated tetravalent linking with CMM **4a** enabled high binding affinity at the nanomolar level with CD206 selectivity (Figure 3).

In addition to specific and strong binding activity to CD206, it is essential for targeted delivery to enhance uptake into receptor-expressing cells and thereby promote gene silencing and protein downregulation. Our CMM–siRNAs exhibited enhanced uptake and gene silencing effects compared with siRNA and C3N-siRNA as non-CD206-binding compounds (Figures 4 and 5). In other studies, multivalent sugar ligand-conjugated siRNAs have been shown to enhance gene silencing, accompanied by increased valency-dependent uptake (56,57). As expected, valency-dependent uptake, gene silencing, and protein downregulation were observed compared with CMM1–siRNA and CMM4–siRNA. The selectivity of the target mRNA was also confirmed using three types of target sequences. The gene silencing activity of the siB2M(h) conjugate was weaker than that of the siHPRT1-a conjugate in our previous report (57), and the same tendency was observed in this study. Although optimization of the sequence and modification pattern in this study has not been fully investigated, these results indicate that siRNA optimization is also important for validating the conjugate.

Regarding the mechanism of CMM–siRNAs, we evaluated the amount of siRNA uptake into macrophages and DCs required for gene silencing. Interestingly, we found that the increased uptake ratio of siRNAs between CMM4–siRNA and siRNA for each type of CD206-expressing cell (Supplementary Figure S2) was not always consistent with the increased gene silencing ratio (Figures 4 and 6). In addition, considering the uptake of CMM4–siRNA required for gene silencing activity in each type of CD206-expressing cell, it is clear that the degree of gene silencing activity did not necessarily depend on the uptake of siRNA. Although we can attribute this result to differences in the intracellular environment, such as different metabolic activities and characteristics of cytosolic transfer among cells (58), the precise cause remains unknown. Further analyses, such as time-dependent metabolic stability analysis and evaluation of the cytosolic transfer efficiency based on comparisons of siRNA uptake and quantification of the RNA-induced silencing complex loading number of nucleic acids, will be needed in order to elucidate the detailed mechanisms. Recently, in their investigation of intracellular trafficking of siRNAs using LNP–siRNA and GalNAc–siRNA, several groups reported valuable data to help understand the mechanism (59,60); however, to the best of our knowledge, few reports have evaluated the intracellular trafficking of extrahepatic-targeting ligand-siRNA conjugates. Thus, further metabolic stabilization of siRNAs and cytosolic transfer from endosomes/lysosomes to the cytosol (endosomal escape) could clarify potential strategies for gene silencing in extrahepatic target organs and cells.

Although few reports have described receptor-mediated targeted delivery to extrahepatic organs and cells using ligand-conjugated siRNAs, the correlations of KD with accumulation on receptor-expressing cells using ligand-conjugated siRNAs for extrahepatic targeting have not been extensively evaluated (17–21). To confirm the targeting ability and gene silencing effects of these conjugates *in vivo*, we assessed targeted delivery to CD206-expressing tissue-resident macrophages using wild-type mice because CD206 is known to be expressed by tissue-resident macrophages (red pulp macrophages, hepatic macrophages, alveolar macrophages, and sinusoidal endothelial cells, among other cell types) in these mice (61–64). GalNAc4–siRNA and siRNA selectively accumulated in the liver and kidneys, respectively, consistent with previous reports (58,65). By contrast, although CMM4–siRNA accumulated in the liver, spleen, and kidneys, this conjugate accumulated only in areas containing CD206-positive cells (i.e. strongly accumulated in CD206-positive cells compared with siRNA), not throughout the whole tissue, as determined by FCM (Figure 7).

Consistent with this accumulation, CMM4–siRNA showed gene silencing effects in CD206-positive macrophages in the spleen and liver (Figure 8B, C, and Supplementary Figure S7). These results clearly demonstrated the selective accumulation in and gene silencing of splenic and hepatic CD206-expressing cells. However, similar to our findings in kidney macrophages, while CMM4–siRNA accumulated on CD206-positive kidney macrophages, enhancement of gene silencing in these cells, as analyzed by RT-qPCR, was not observed (Figure 8D). According to the accumulation profile of CMM4–siRNA *in vivo*, this conjugate tends to mainly accumulate in CD206-positive macrophages in the kidneys. Therefore, to clarify the gene silencing profiles of this conjugate in kidney macrophages, we investigated the protein downregulation profiles in CD206 high-positive (CD206^high^) and CD206 low-positive (CD206^low^) populations of macrophages using CMM4–siRNA (Figure 12). In the CD206^high^ population, CMM4–siRNA enhanced protein downregulation compared with ligand-unconjugated siRNA. Contrarily, the protein downregulation effect was weak in the CD206^low^ population. Therefore, these results indicated that our conjugates exhibited receptor expression-dependent gene silencing and protein downregulation in CD206-positive macrophages in the kidneys. However, although CD206 expression was almost the same in both splenic and kidney macrophages (Supplementary Figure S6), the gene silencing characteristics seemed to differ between splenic and kidney macrophages. As with the *in vitro* profile, a detailed mechanistic evaluation should be performed in future studies to fully clarify the gene silencing and protein downregulation profiles of each cell type.

This study was limited by its assessment of the targeted delivery of siRNAs using only wild-type mice. We, therefore, have yet to explore the impact of siRNA delivery using a disease model. In the future, when creating novel drugs using this technology, we should pay attention to the clinical relevance of mouse models with regard to human disease. Further examinations should be conducted using animal models that properly reflect CD206 expression in each human disease. Moreover, there was no clear toxicity from the observation of body weight and organ structure after treatment with CMM4–siRNA at 10 mg/kg, and other parameters associated with toxicity, such as observation of liver toxicity and cytokine release, in murine and nonhuman primates are yet to be evaluated. Such evaluations should be performed in the future.

In conclusion, in this study, we presented a proof-of-concept study of the targeted delivery of contents to macrophages and DCs using a CD206-targetable modified mannose–siRNA conjugate as a simplified, novel, targeted delivery platform for nucleic acid-based drugs. Our findings support the potential development of treatments for diseases caused by or associated with aberrant gene expression in macrophages and DCs. Moreover, these findings are expected to lead to the development of new targeting siRNA-based therapeutics for non-hepatic cases.

## DATA AVAILABILITY

The authors declare that all data supporting the findings of this study are available within the article and the Supplementary Information files.

## Supplementary Material

gkac308_Supplemental_File
