# Supplementary material for: Targeted delivery to macrophages and dendritic cells by chemically modified mannose ligand-conjugated siRNA
Source: Nucleic Acids Res. 2022 May 7;50(9):4840–59. doi: 10.1093/nar/gkac308 (PMC9122583; doi:10.1093/nar/gkac308)
Supplement: gkac308_Supplemental_File [file gkac308_supplemental_file.pdf]

Supplementary Information

Targeted delivery to macrophages and dendritic cells by chemically modified mannose ligand-conjugated siRNA

Keiji Uehara[1]*, Toshimasa Harumoto[1], Asana Makino[1], Yasuo Koda[1], Junko Iwano[2], Yasuhiro Suzuki[1], Mari Tanigawa[1], Hiroto Iwai[1], Kana Asano[1], Kana Kurihara[1], Akinori Hamaguchi[1], Hiroshi Kodaira[2], Toshiyuki Atsumi[1], Yoji Yamada[1], Kazuma Tomizuka[1]

[1] Research Unit, R&D Division, Kyowa Kirin Co., Ltd., Otemachi Financial City Grand Cube, 1-9-2 Otemachi, Chiyoda-ku, Tokyo 100-0004, Japan
[2] Translational Research Unit, R&D Division, Kyowa Kirin Co., Ltd., 1188 Shimotogari, Nagaizumi-cho, Sunto-gun, Shizuoka, 411-8731, Japan

* To whom correspondence should be addressed. Tel: +81-80-7200-7306;

Email: keiji.uehara.vk@kyowakirin.com

This PDF file includes:

Figure S1. Preparation and characterization of human monocyte-derived M1 macrophages, M2a macrophages, immature DCs and mature DCs.

Figure S2. *In vitro* cellular uptake of CMM4-siHPRT1-a in human monocyte-derived M1 macrophages, M2a macrophages, immature DCs, and mature DCs.

Figure S3. Fluorescence images of AF488-labeled CMM4-siHPRT1 and siHPRT1 in M2a macrophages.

Figure S4. *In vitro* gene silencing on human primary hepatocytes.

Figure S5. An analysis of the principal receptors for the uptake of CMM4-siHPRT1-a.

Figure S6. Purity of splenic, hepatic, kidney F4/80-positive cells isolated by F4/80 mAb-loaded magnetic beads.

Figure S7. Gene-silencing activities of siRNA conjugate on whole spleen and whole liver.

Supplementary Text

Analytical data

Supplementary References

Supplementary Figure

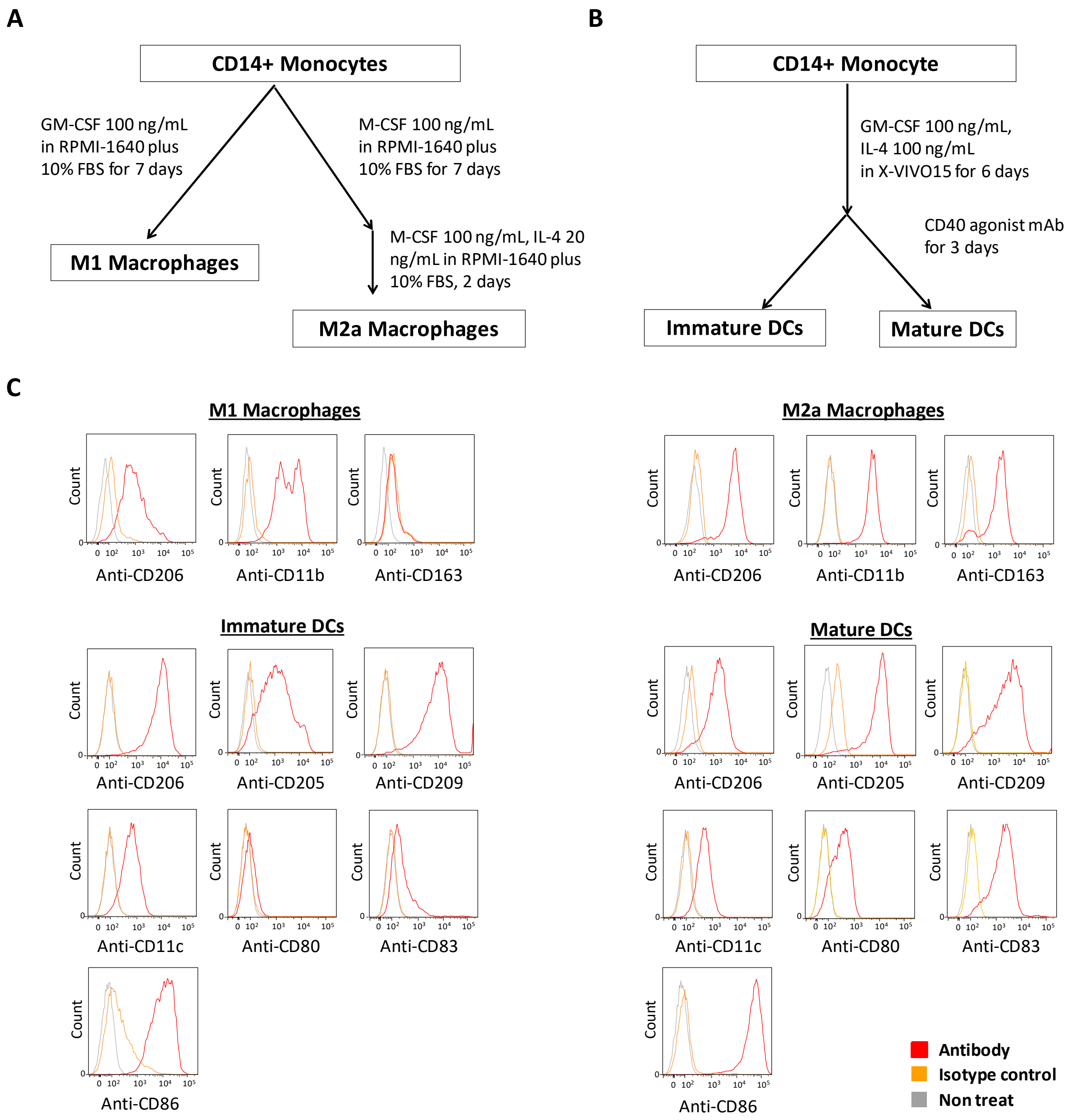

Figure S1. Preparation and characterization of human monocyte-derived M1 macrophages, M2a macrophages, immature DCs and mature DCs. (A) Preparation scheme of human monocyte-derived M1 and M2a macrophages. (B) Preparation scheme of human monocyte-derived immature and mature DCs. (C) Protein expression on the surface of human monocyte-derived M1 macrophages, M2a macrophages, immature DCs, and mature DCs, as analyzed by FCM.

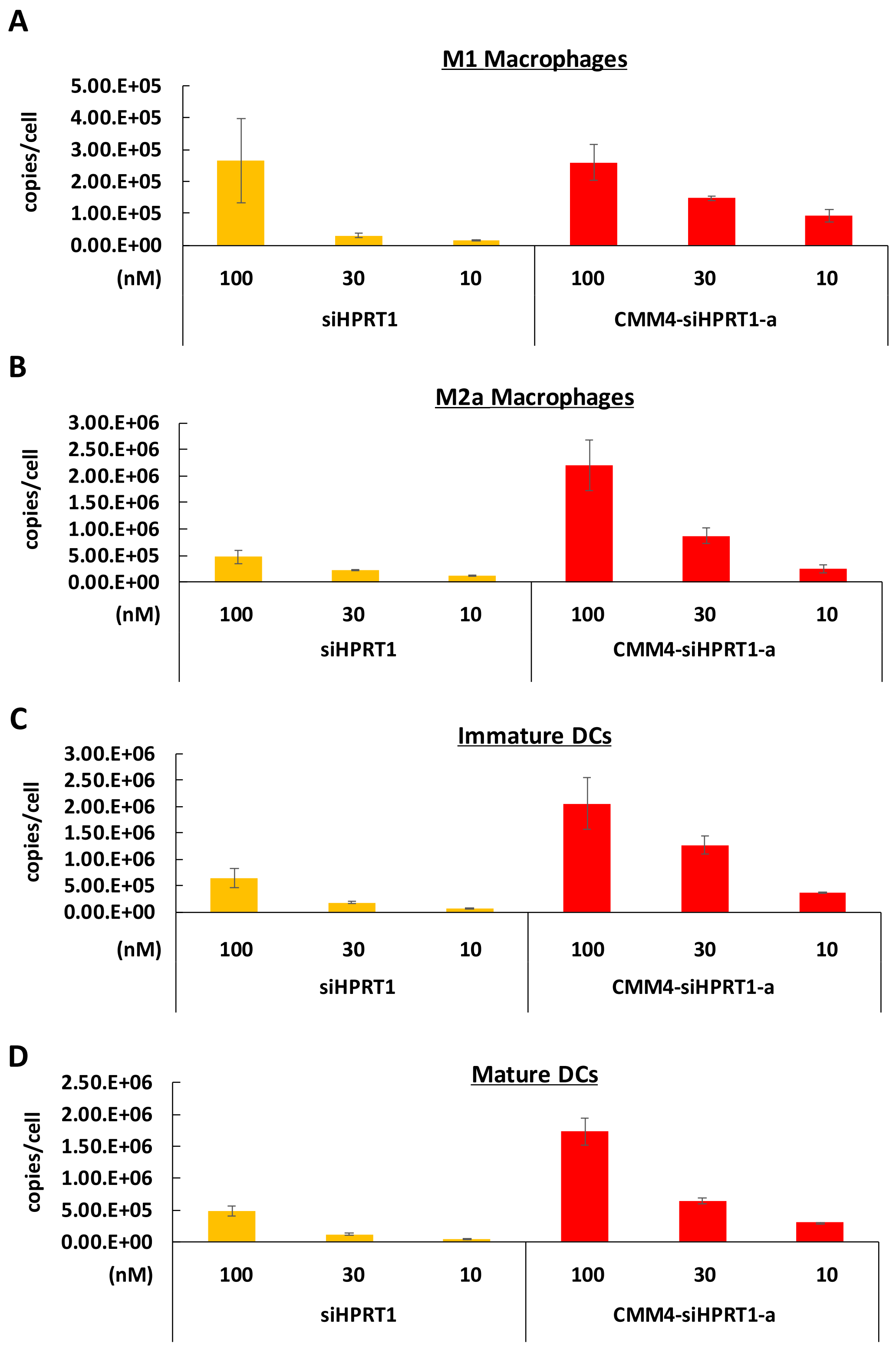

Figure S2. ***In vitro*** **cellular uptake of CMM4-siHPRT1-a in human monocyte-derived M1 macrophages, M2a macrophages, immature DCs, and mature DCs.** Cells were transfected with CMM4-siHPRT1 or siHPRT1 for 6 h. The cellular uptake of each siRNA was analyzed by stem-loop RT-qPCR. Results are expressed as means ± standard deviations of triplicate experiments.

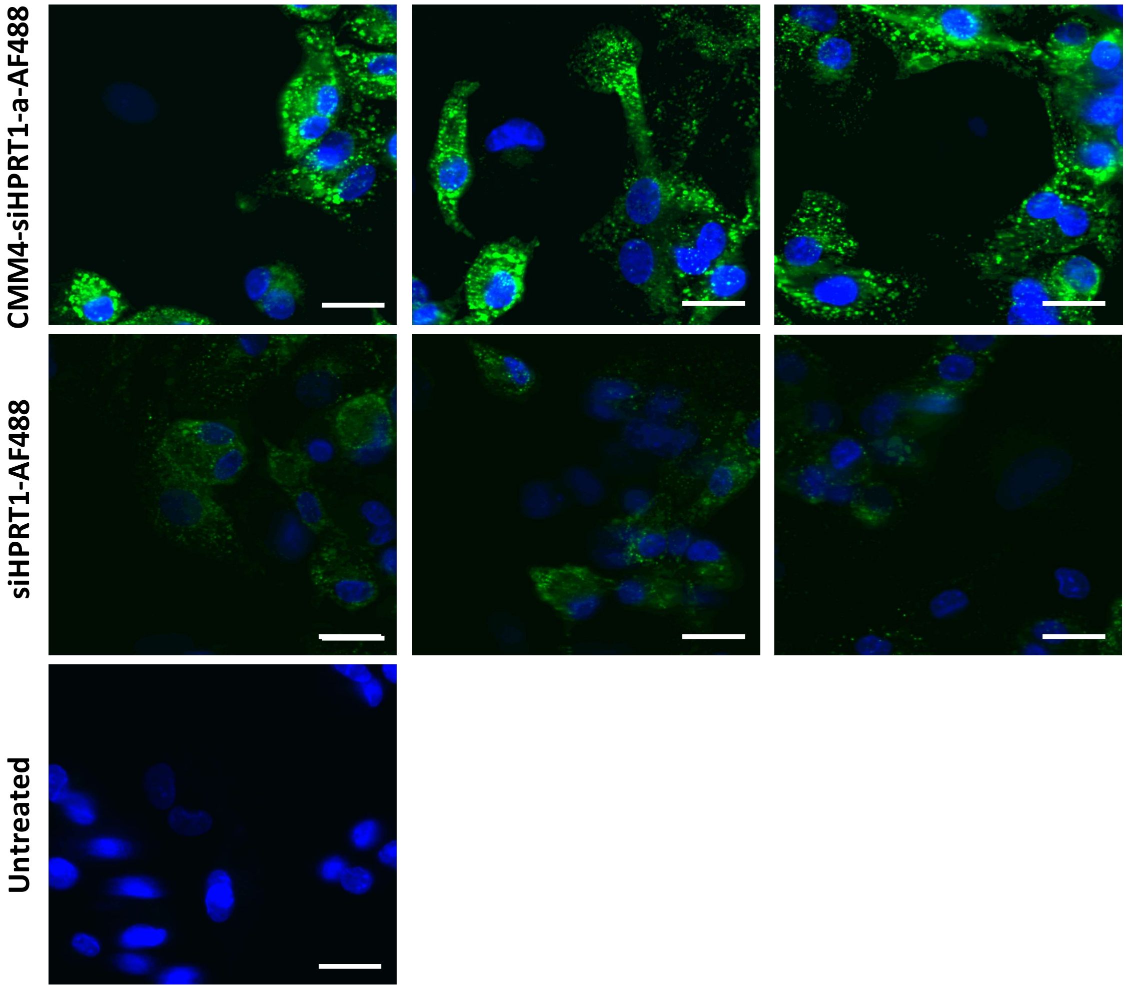

Figure S3. Fluorescence images of AF488-labeled CMM4-siHPRT1 and siHPRT1 in M2a macrophages. Confocal microscopy images of M2a macrophages at 1 h after treatment with AF488-labeled CMM4-siHPRT1 or siHPRT1 at a concentration of 30 nM. Nuclei were stained with Hoechst 33342. Green represents AF488-labeled CMM4-siHPRT1 siHPRT1 or siHPRT1. Three fields are shown for each sample. Scale bars, 20 μm.

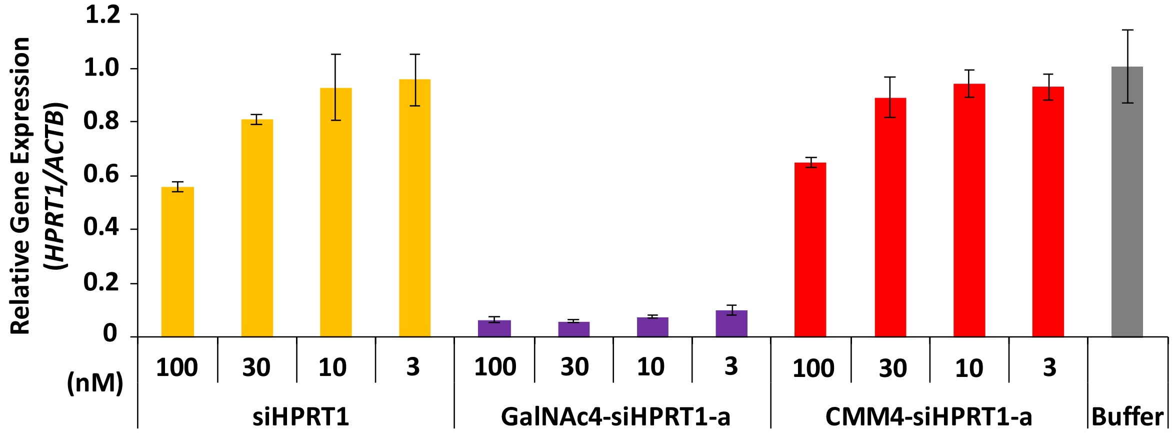

Figure S4. *In vitro* gene silencing on human primary hepatocytes. Gene-silencing activities of siRNA conjugate on human primary hepatocytes expressing ASGPR but not CD206. Each HPRT1-targeting siRNA was added for 6 h. After changing the medium, the HPRT1 mRNA expression was quantified at 24 h by quantitative PCR and normalized to that of ACTB mRNA. Results are expressed as means ± standard deviations of triplicate experiments.

A

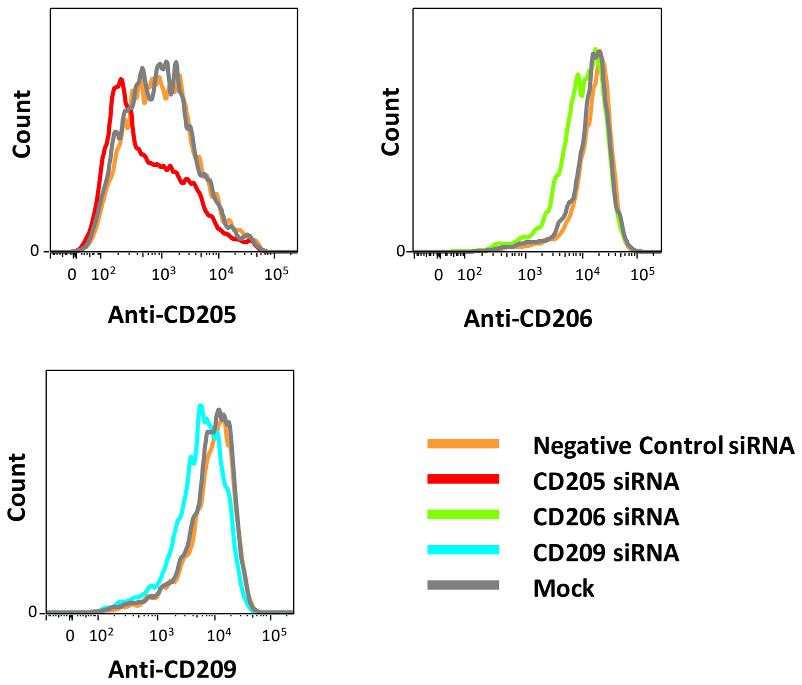

B

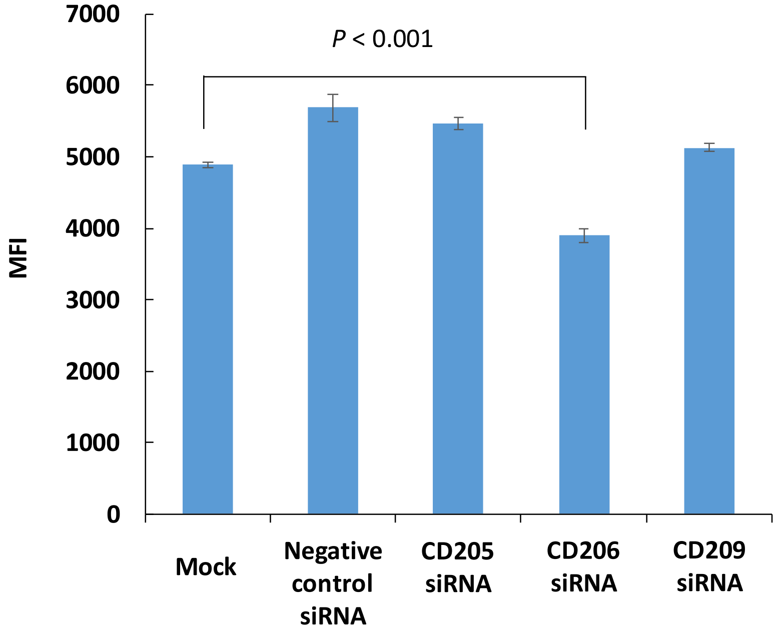

Figure S5. An analysis of the principal receptors for the uptake of CMM4-siHPRT1-a. (A) Each siRNA targeting C-type lectin (CD205, CD206 and CD209) was transfected to immature DCs at 5 nmol/L. After four days, the expression of the receptor was analyzed by flow cytometry. (B) CMM4-siHPRT1-a was added to siRNA-transfected immature DCs at 30 nmol/L for 1 h. The fluorescence intensity was measured by flow cytometry, and the geometric mean fluorescence intensity (MFI) was calculated. The values represent the mean ± SD of triplicate experiments.

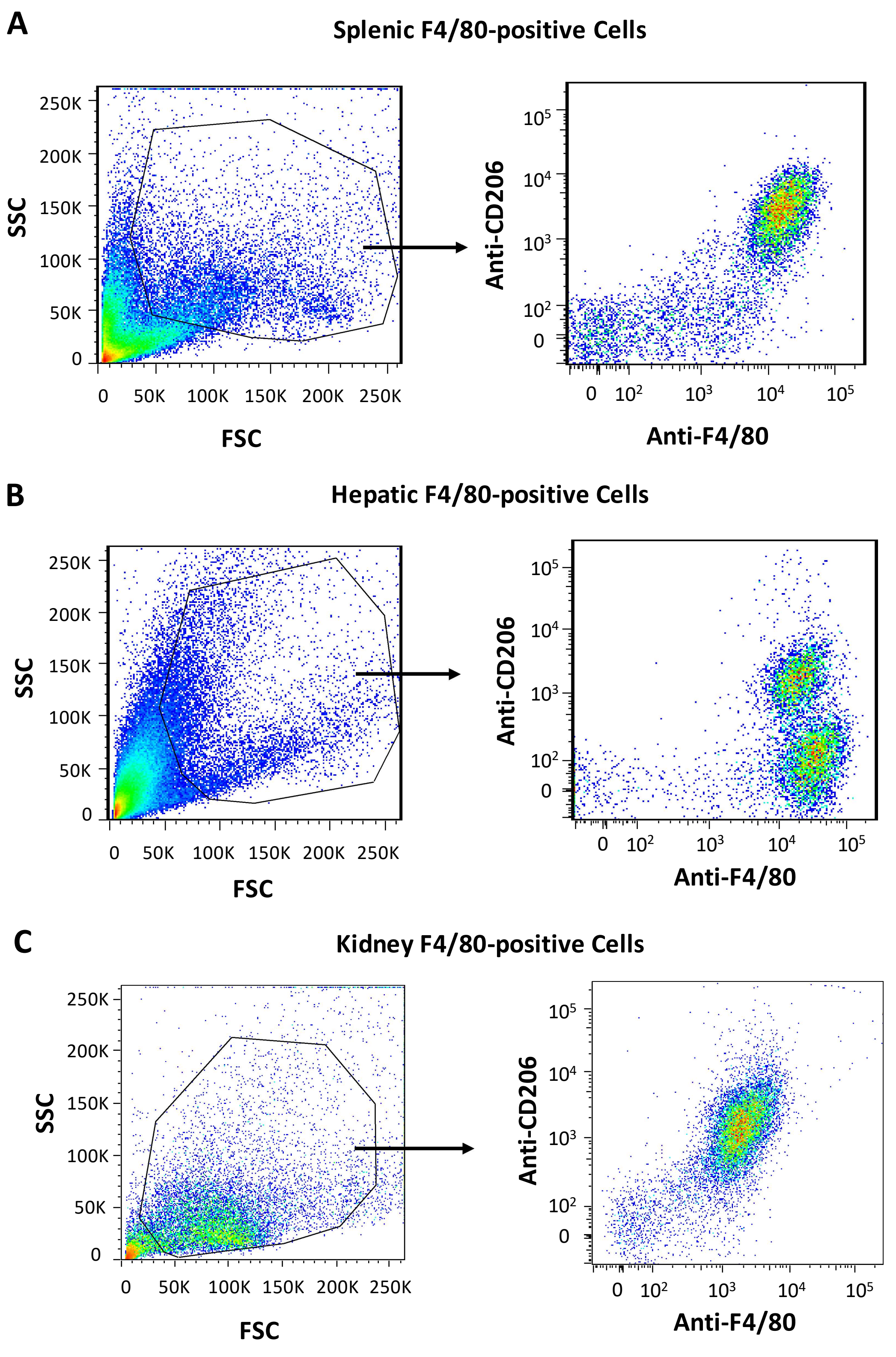

Figure S6. Purity of splenic, hepatic, kidney F4/80-positive cells isolated by F4/80 mAb-loaded magnetic beads. Splenic (A), hepatic (B), and kidney (C) cells were dissociated, and F4/80-positive cells were isolated using anti-F4/80 MicroBeads. The purity of the F4/80-positive cell population was determined by FCM using antibodies to F4/80 and CD206 markers.

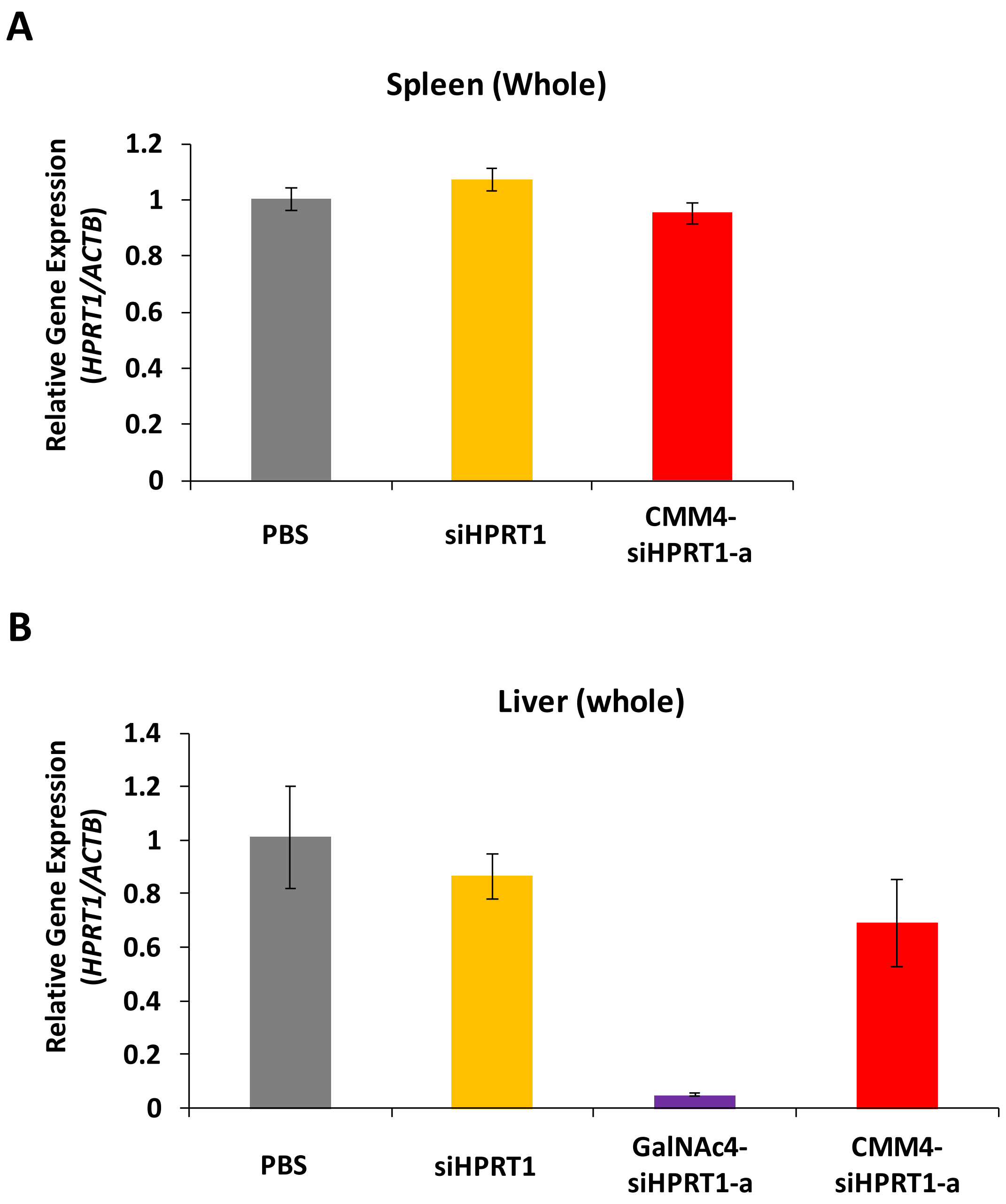

Figure S7. Gene-silencing activities of siRNA conjugate on whole spleen and whole liver. siHPRT1, GalNAc-siHPRT1, and CMM4-siHPRT1-a conjugates were administered via a single subcutaneous injection (10 mg/kg). Four days after administration, whole spleens and livers were collected and analyzed by RT-qPCR. *Hprt1* mRNA expression in whole spleens (A) and livers (B) was determined using quantitative PCR and normalized to the expression of *Actb* mRNA. Results are expressed as means ± standard deviations of triplicate experiments.

Supplementary Text

General information of chemical synthesis

Reagents and solvents obtained from commercial suppliers were used without purification or drying unless otherwise noted. Methyl α-D-mannopyranoside was purchased from FUJIFILM Wako Pure Chemical Corporation. Compound **1** was prepared as described in the literature (1). Compound **15** was prepared as described in the literature (2).

^{1}H NMR was recorded by a Bruker instrument at 400 and 500 MHz. ^{13}C NMR was recorded by a Bruker instrument at 101 MHz. ESI-MS was recorded by LC-MS system using Waters Aquity UPLC system with Waters SQ Detecor, Waters Photodiode Array Detector and Waters ELS Detector modules.

<u>LC-MS analysis</u>
Column: AQUITY UPLC BEH C18 (1.7 µm, 2.1 x 50 mm, Waters), Bufferr A: 0.05% formic acid, Buffer B: 0.05% MeCN, Program: liner gradient of 10-90% buffer B over 7 min, Flow rate: 0.6 mL/min, Column Temperature: 40 °C

<u>Synthesis of chemically modified mannose monomers **4a–f**</u>

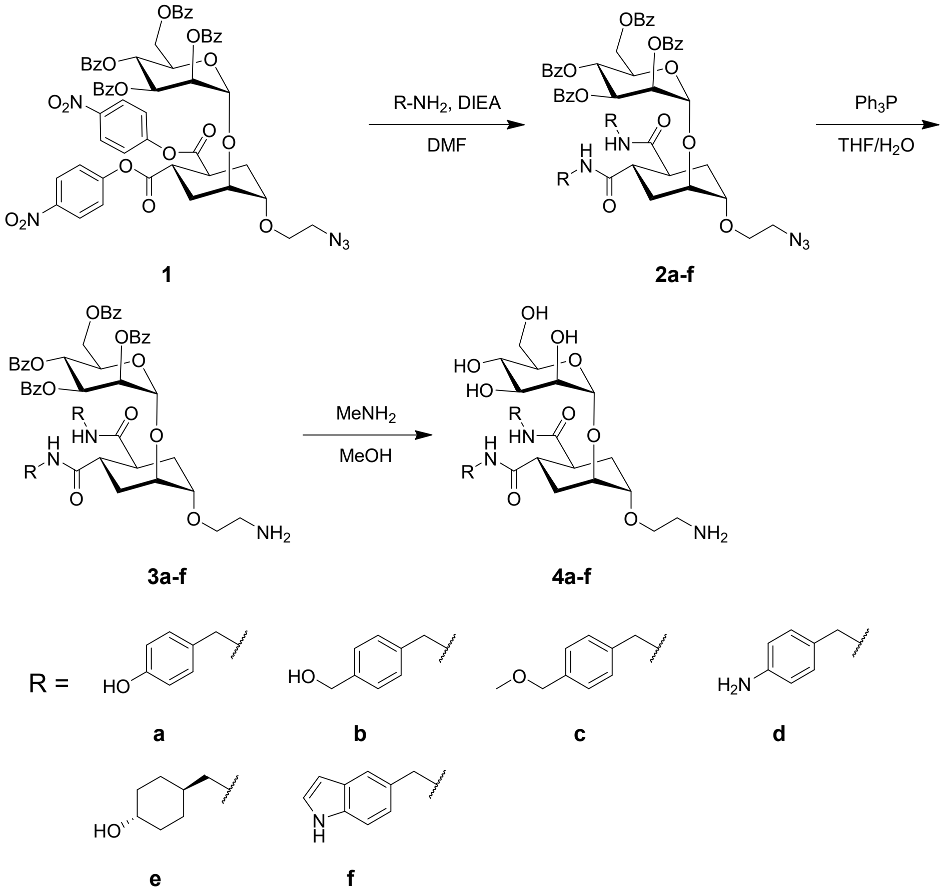

General procedure 1: Condensation using alkylamine

To a 0.1 M stirred solution of compound **1** (1 eq) in DMF, we added *N*, *N*-diisopropylethylamine (DIEA, 6 eq) and alkylamine (2.5 eq) at room temperature, and the reaction mixture was stirred at the same temperature for 48 h. The reaction was monitored by thin-layer chromatography (TLC). After completion of the reaction, the reaction mixture was evaporated under reduced pressure to give the crude compound. The crude compound was purified by reverse-phase column chromatography using a grace instrument, eluted in 10% MeCN/water (0.1% formic acid), and concentrated under reduced pressure to afford compounds **2a–f.**

General procedure 2: Reduction using triphenylphosphine

To a 0.05 M stirred solution of compounds **2a–f** (1 eq) in THF/H_2O (100:1), we added triphenylphosphine (Ph_3P, 1.2 eq) at room temperature, and the reaction mixture was stirred at the same temperature for 20 h. The reaction was monitored by TLC. After completion of the reaction, the reaction mixture was evaporated under reduced pressure to give a residue. The residue was washed with n-pentane to give a crude compound. The crude compound was purified by preparative HPLC to afford compounds **3a–f.**

General procedure 3: Deprotection using sodium methoxide

Compounds **3a–f** (1 eq) were dissolved in 2 M methylamine in MeOH to yield a 0.05 M solution at room temperature, and the reaction mixture was stirred at the same temperature for 16 h. The reaction was monitored by LC-MS. After completion of the reaction, the reaction mixture was evaporated under reduced pressure to give a crude compound. The crude compound was purified by preparative HPLC to afford compounds **4a–f.**

Compound 2a: Using general procedure 1 starting from 4-(aminomethyl)phenol; obtained as crude mixture

ESI-MS *m*/*z*: 1062 $[M + H]^+$

Compound 3a: Using general procedure 2 starting from compound **2a**; yield: 32% over two steps.

^1H NMR (500 MHz, DMSO-*d*6) δ 1.77-1.94 (4H, m), 2.64-2.67 (2H, m), 2.84-2.85 (2H, m), 3.37-3.40 (1H, m), 3.49-3.52 (1H, m), 3.71-3.72 (1H, m), 4.02-4.06 (2H, m), 4.10-4.16 (3H, m), 4.54 (2H, d, *J* = 9.16 Hz), 4.62-4.64 (1H, m), 5.42 (1H, brs), 5.73-5.76 (2H, m), 5.93 (1H, t, *J* = 9.61 Hz), 6.64-6.70 (4H, m), 6.99-7.05 (4H, m), 7.36 (2H, t, *J* = 7.78 Hz), 7.45-7.51 (4H, m), 7.53-7.69 (6H, m), 7.71-7.75 (2H, m), 7.93 (4H, dd, *J* = 13.43, 7.63 Hz), 8.05 (2H, d, *J* = 7.32 Hz), 8.21-8.25 (2H, m), 9.22 (2H, brs). NH_2 protons are omitted. ESI-MS *m*/*z*: 1037 $[M + H]^+$

Compound 4a: Using general procedure 3 starting from compound **3a**; yield: 78%

^{1}H NMR (400 MHz, CD_3OD) δ: 1.89-1.93 (4H, m), 2.78-2.88 (4H, m), 3.50-3.63 (4H, m), 3.65-3.79 (3H, m), 3.83-3.87 (1H, m), 3.88 (1H, dd, J = 3.4, 1.6 Hz), 4.02 (1H, q, J = 2.9 Hz), 4.13-4.22 (4H, m), 4.92 (1H, d, J = 1.8 Hz), 6.70-6.73 (4H, m), 7.06-7.08 (4H, m). ^{13}C NMR (101 MHz, CD_3OD) δ: 28.7, 29.6, 41.8, 41.9, 42.2, 43.6, 63.1, 68.8, 70.5, 72.2, 72.4, 72.6, 75.5, 76.1, 100.1, 116.2, 116.2, 129.9, 130.7, 157.7, 176.8, 176.8. ESI-MS *m*/*z*: 620.46 $[M + H]^+$

Compound 2b: Using general procedure 1 starting from (4-[aminomethyl]phenyl)methanol; yield: 65%.

1H NMR (400 MHz, $CDCl_3$) δ 1.72-2.06 (2H, m), 2.15-2.27 (2H, m), 2.95-3.03 (2H, m), 3.22-3.33 (2H, m), 3.54-3.62 (2H, m), 3.77-3.78 (1H, m), 4.08 (1H, brs), 4.29-4.40 (3H, m), 4.40-4.44 (1H, m), 4.48-4.52 (2H, m), 4.54-4.63 (4H, m), 4.65-4.72 (1H, m), 5.26 (1H, d, J = 1.71 Hz), 5.69 (1H, dd, J = 3.18, 1.71 Hz), 5.88 (1H, dd, J = 10.4, 3.6 Hz), 6.10-6.15 (1H, m), 6.39 (1H, t, J = 5.99 Hz), 7.10 (1H, t, J = 5.75 Hz), 7.17-7.24 (9H, m), 7.31-7.44 (7H, m), 7.50-7.61 (3H, m), 7.73 (2H, dd, J = 8.31, 1.22 Hz), 7.95-8.02 (4H, m), 8.07 (2H, dd, J = 8.44, 1.34 Hz). NH protons are omitted.

ESI-MS *m*/*z*: 1090.72 $[M + H]^+$

Compound 3b: Using general procedure 2 starting from compound **2b**; yield: 59%.

1H NMR (400 MHz, $CDCl_3$) δ 1.72-2.06 (2H, m), 2.15-2.27 (2H, m), 2.95-3.03 (2H, m), 3.22-3.33 (2H, m), 3.54-3.62 (2H, m), 3.77-3.78 (1H, m), 4.08 (1H, brs), 4.29-4.40 (3H, m), 4.40-4.44 (1H, m), 4.48-4.52 (2H, m), 4.54-4.63 (4H, m), 4.65-4.72 (1H, m), 5.26 (1H, d, J = 1.71 Hz), 5.69 (1H, dd, J = 3.18, 1.71 Hz), 5.88 (1H, dd, J = 10.4, 3.6 Hz), 6.10-6.15 (1H, m), 6.39 (1H, t, J = 5.99 Hz), 7.10 (1H, t, J = 5.75 Hz), 7.17-7.24 (9H, m), 7.31-7.44 (7H, m), 7.50-7.61 (3H, m), 7.73 (2H, dd, J = 8.31, 1.22 Hz), 7.95-8.02 (4H, m), 8.07 (2H, dd, J = 8.44, 1.34 Hz). NH protons are omitted.

ESI-MS *m*/*z*: 1090.72 $[M + H]^+$

Compound 4b: Using general procedure 3 starting from compound **3b**; yield: 36%.

^{1}H NMR (400 MHz, CD_3OD) δ: 1.95-1.99 (4H, m), 2.85-2.96 (2H, m), 3.15-3.19 (2H, m), 3.57-3.92 (9H, m), 4.06 (1H, q, J = 2.9 Hz), 4.32 (4H, s), 4.58 (4H, s), 4.96 (1H, d, J = 1.8 Hz), 7.24 (4H, dd, J = 8.2, 1.9 Hz), 7.30 (4H, d, J = 8.1 Hz). ^{13}C NMR (101 MHz, CD_3OD) δ: 28.7, 29.1, 40.9, 41.6, 41.8, 43.7, 63.2, 64.9, 65.7, 68.8, 71.9, 72.3, 72.6, 75.6, 76.5, 100.3, 128.2, 128.2, 128.4, 128.4, 138.9, 139.0, 141.6, 141.7, 176.6, 177.1.

ESI-MS *m*/*z*: 648.48 $[M + H]^+$

Compound 2c: Using general procedure 1 starting from (4-[methoxymethyl]phenyl)methanamine; obtained as a crude mixture.

ESI-MS *m/z*: 1118.68 [M + H]$^+$

Compound 3c: Using general procedure 2 starting from compound **2c**; yield: 39% over two steps.

^{1}H NMR (500 MHz, DMSO-d6) δ 1.80 (1H, t, J = 12.05 Hz), 1.93-1.96 (3H, m), 2.67 (2H, t, J = 5.34 Hz), 2.89-2.92 (2H, m), 3.24 (3H, s), 3.26 (3H, s), 3.38-3.42 (1H, m), 3.51-3.53 (1H, m), 3.73-3.74 (1H, m), 4.08-4.09 (1H, m), 4.18-4.37 (8H, m), 4.54-4.65 (3H, m), 5.43 (1H, s), 5.74-5.77 (2H, m), 5.89-5.98 (1H, m), 7.18-7.24 (8H, m), 7.36 (2H, t, J = 7.78 Hz), 7.45-7.75 (10H, m), 7.94 (4H, dd, J = 14.34, 7.32 Hz), 8.05 (2H, d, J = 7.32 Hz), 8.38-8.42 (2H, m). NH_2 protons are omitted.

ESI-MS *m/z*: 1092.87 [M + H]$^+$

Compound 4c: Using general procedure 3 starting from compound **3c**; yield: 38%.

^{1}H NMR (400 MHz, CD_3OD) δ: 1.92-1.99 (4H, m), 2.87-2.90 (2H, m), 3.14-3.17 (2H, m), 3.34 (3H, s), 3.34 (3H, s), 3.55-3.90 (9H, m), 4.04 (1H, q, J = 2.9 Hz), 4.30 (4H, t, J = 4.4 Hz), 4.41 (4H, s), 4.94 (1H, d, J = 1.5 Hz), 7.21-7.27 (8H, m). ^{13}C NMR (101 MHz, CD_3OD) δ: 28.7, 29.1, 40.9, 41.6, 41.8, 43.7, 58.2, 63.2, 65.7, 68.8, 71.9, 72.3, 72.6, 75.4, 75.6, 76.5, 100.3, 128.4, 128.5, 129.1, 129.1, 138.3, 138.4, 139.5, 139.5, 176.6, 177.1.

ESI-MS *m/z*: 676.50 [M + H]$^+$

Compound 2d: Using general procedure 1 starting from 4-(aminomethyl)aniline; yield: 54%.

^{1}H NMR (400 MHz, $CDCl_3$) δ 1.97-2.03 (2H, m), 2.14- 2.22 (2H, m), 2.89 (2H, t, J = 12.2 Hz), 3.24-3.28 (2H, m), 3.49-3.64 (2H, m), 3.72-3.76 (2H, m), 4.15-4.40 (3H, m), 4.41-4.60 (2H, m), 4.66-4.68 (1H, m), 5.25 (1H, m), 5.64-5.66 (1H, m), 5.85-5.89 (1H, m), 6.09-6.11 (2H, m), 6.59-6.68 (5H, m), 7.04-7.09 (4H, m), 7.37-7.45 (10H, m), 7.51-7.61 (4H, m), 7.72-7.74 (2H, m), 7.96-8.07 (8H, m).

ESI-MS *m/z*: 1060.07 [M + H]$^+$

Compound 3d: Using general procedure 2 starting from compound **2d**; yield: obtained as a crude mixture.

ESI-MS *m*/*z*: 1034.53 $[M + H]^+$

Compound 4d: Using general procedure 3 starting from compound **3d**; yield: 49% over two steps

^{1}H NMR (400 MHz, CD_3OD) δ: 1.84-1.96 (4H, m), 2.79-2.89 (4H, m), 3.50-3.79 (8H, m), 3.84 (1H, dd, J = 11.7, 1.8 Hz), 3.88 (1H, dd, J = 3.3, 1.8 Hz), 4.01 (1H, q, J = 2.9 Hz), 4.13-4.17 (4H, m), 4.92 (1H, d, J = 1.8 Hz), 6.65-6.69 (4H, m), 7.00 (4H, dt, J = 9.0, 2.3 Hz). ^{13}C NMR (101 MHz, CD_3OD) δ: 28.7, 29.5, 41.8, 41.9, 42.2, 43.7, 63.1, 68.8, 70.3, 72.2, 72.4, 72.6, 75.5, 76.1, 100.1, 116.6, 129.3, 129.6, 147.8, 176.7, 176.8.

ESI-MS *m*/*z*: 618.46 $[M + H]^+$

Compound 2e: Using general procedure 1 starting from (1r,4r)-4-(aminomethyl)cyclohexan-1-ol hydrochloride; obtained as a crude mixture.

ESI-MS *m*/*z*: 1074.62 $[M + H]^+$

Compound 3e: Using general procedure 2 starting from compound **2e**; obtained as a crude mixture.

ESI-MS *m*/*z*: 1048.62 $[M + H]^+$

Compound 4e: Using general procedure 3 starting from compound **3e**; yield: 30% over three steps.

^{1}H NMR (400 MHz, CD_3OD) δ: 0.92-1.01 (4H, m), 1.16-1.29 (4H, m), 1.36-1.39 (2H, m), 1.79-1.90 (12H, m), 2.77-2.85 (4H, m), 2.90-3.03 (4H, m), 3.43-3.79 (9H, m), 3.85 (1H, dd, J = 11.8, 1.9 Hz), 3.90 (1H, dd, J = 3.4, 1.6 Hz), 4.02 (1H, d, J = 3.0 Hz), 4.94 (1H, d, J = 1.5 Hz). ^{13}C NMR (101 MHz, CD_3OD) δ: 28.9, 29.8, 29.9, 29.9, 35.9, 38.4, 38.5, 41.8, 41.9, 42.4, 46.0, 63.1, 68.8, 70.9, 71.4, 72.3, 72.4, 72.6, 75.5, 76.1, 100.2, 177.1.

ESI-MS *m*/*z*: 632.53 $[M + H]^+$

Compound 2f: Using general procedure 1 starting from (1H-indol-5-yl)methanamine; yield: 65%.

^{1}H NMR (500 MHz, $CDCl_3$) δ 1.99-2.07 (1H, m), 2.21-2.25 (3H, m), 2.88-3.10 (2H, m), 3.24-3.26 (2H, m), 3.53-3.56 (1H, m), 3.57-3.59 (1H, m), 3.77 (1H, d, *J* = 3.0 Hz), 4.07 (1H, d, *J* = 2.0 Hz), 4.29 (1H, dd, *J* = 14.0, 9.0 Hz), 4.35-4.39 (1H, m), 4.45-4.54 (4H, m), 4.67 (1H, d, *J* = 9.5 Hz), 5.25 (1H, d, *J* = 1.5 Hz), 5.63-5.67 (1H, m), 5.83-5.89 (1H, m), 6.10 (1H, t, *J* = 5.0 Hz), 6.24 (1H, t, *J* = 5.0 Hz), 6.51-6.53 (2H, m), 6.82 (1H, t, *J* = 10.0 Hz), 7.03-7.10 (2H, m), 7.11-7.15 (2H, m), 7.17-7.23 (2H, m), 7.27-7.29 (2H, s), 7.31-7.39 (m, 8H), 7.50-7.56 (4H, m), 7.64 (2H, dd, *J* = 8.5, 1.0 Hz), 7.91 (2H, dd, *J* = 8.5, 1.5 Hz), 8.00-8.07 (4H, m), 8.10-8.21 (2H, m).

ESI-MS *m*/*z*: 1108.61 $[M + H]^+$

Compound 3f: Using general procedure 2 starting from compound **2f**; obtained as a crude mixture.

ESI-MS *m*/*z*: 1082.71 $[M + H]^+$

Compound 4f: Using general procedure 3 starting from compound **3f**; yield: 47% over two steps.

^{1}H NMR (400 MHz, CD_3OD) δ: 1.87-2.00 (4H, m), 2.83-2.87 (4H, m), 3.50-3.79 (8H, m), 3.84 (1H, dd, J = 11.8, 1.4 Hz), 3.88 (1H, dd, J = 3.2, 1.6 Hz), 4.02 (1H, q, J = 3.0 Hz), 4.25 (2H, d, J = 14.4 Hz), 4.36 (2H, dd, J = 14.4, 4.1 Hz), 4.92 (1H, d, J = 1.8 Hz), 6.39 (2H, d, J = 3.0 Hz), 6.98 (2H, dt, J = 8.4, 2.0 Hz), 7.20 (2H, d, J = 3.0 Hz), 7.31 (2H, d, J = 8.4 Hz), 7.40 (2H, s). ^{13}C NMR (101 MHz, CD_3OD) δ: 28.7, 29.6, 42.1, 42.2, 42.3, 44.9, 63.3, 69.0, 70.2, 72.3, 72.6, 72.7, 75.7, 76.3, 100.2, 102.5, 112.4, 120.5, 122.6, 126.2, 129.7, 130.4, 137.2, 176.8, 176.8.

ESI-MS *m*/*z*: 666.50 $[M + H]^+$

Synthesis of tetravalent chemically modified mannose linker (CMML4: 11)

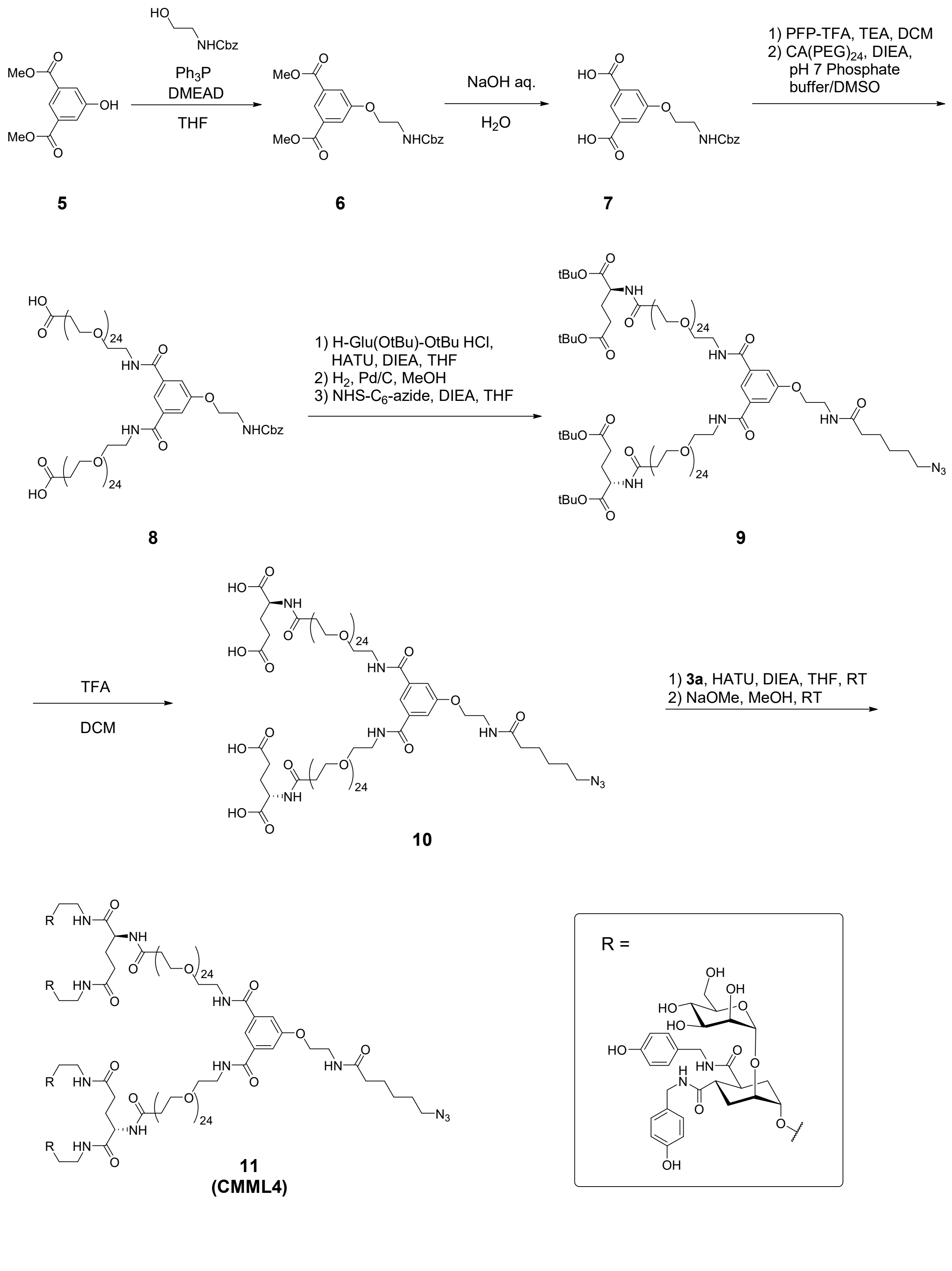

Dimethyl 5-(2-(((benzyloxy)carbonyl)amino)ethoxy)isophthalate (**6**)

To a solution of crude dimethyl 5-hydroxyisophthalate (**5**, 100 mg, 476 μmol) in THF (5 ml) was added benzyl (2-hydroxyethyl) carbamate (139 mg, 714 μmol) and triphenyl phosphine (476 mg, 1.43 mmol), followed by the addition of bis(2-methoxyethyl) azodicarboxylate (DMEAD, 167 mg, 714 μmol). After stirring overnight at room temperature, ethyl acetate and water were added to the reaction mixture. The aqueous phase was extracted with ethyl acetate. The combined organic layers were washed with water and brine, dried with $MgSO_4$ and concentrated *in vacuo*. The residue was separated by silica gel chromatography, and compound **6** was obtained as a white solid (73.0 mg, 40%).

^{1}H-NMR (400 MHz, $CDCl_3$) δ: 3.65 (2H, q, *J* = 5.4 Hz), 3.94 (6H, s), 4.13 (2H, t, *J* = 5.3 Hz), 5.12 (2H, s), 5.20 (1H, br s), 7.29-7.39 (5H, m), 7.73 (2H, d, *J* = 1.3 Hz), 8.29 (1H, t, *J* = 1.5 Hz). ESI-MS *m/z*: 432 $[M + HCO_2]^-$

5-(2-(((benzyloxy)carbonyl)amino)ethoxy)isophthalic acid (**7**)

To a cooled solution of compound **6** (201 mg, 551 μmol) in methanol (50 ml), 2 mol/L aqueous sodium hydroxide (NaOH aq., 19.7 mL, 39.5 mmol) was gradually added. After stirring overnight at room temperature, the mixture was evaporated and quenched with 10% (wt) aqueous citric acid solution. The aqueous phase was extracted with ethyl acetate. The combined organic layers were washed with water and brine, dried with $MgSO_4$ and concentrated *in vacuo*. Compound **7** was obtained (3.08 g, 87%) and subjected to esterification with pentafluorophenyl trifluoroacetate (PFP-TFA) without further purification.

1,1'-(5-(2-(((benzyloxy)carbonyl)amino)ethoxy)-1,3-phenylene)bis(1-oxo-5,8,11,14,17,20,23,26,29,32,35,38,41,44,47,50,53,56,59,62,65,68,71,74-tetracosaoxa-2-azaheptaheptacontan-77-oic acid) (**8**)

To a cooled solution of compound **7** (40.5 mg, 113 μmol) and triethylamine (TEA, 157 μL, 1.13 mmol) in dichloromethane (DCM, 5.0 ml), PFP-TFA (77 μL, 451 μmol) was gradually added. After 2 h of stirring at room temperature, the mixture was evaporated and quenched with 10% (wt) aqueous citric acid solution. The aqueous phase was extracted with chloroform. The combined organic layers were washed with phosphate buffer and brine, dried with $MgSO_4$ and concentrated *in vacuo*. Crude PFP ester was obtained (77.4 mg).

Next, carboxy-PEG-amine compound (CA(PEG)24; Thermo Scientific; 312 mg, 273 μmol) was added to the solution of PFP ester (77.4 mg) and DIEA (98.0 μL, 564 μmol) in DMSO. After stirring overnight at room temperature, the mixture was evaporated and quenched with 10% (wt) aqueous citric acid solution. The aqueous phase was extracted with chloroform. The combined organic layers were washed with brine, dried with $MgSO_4$ and concentrated *in vacuo*. Compound **8** was obtained (300 mg) and subjected to amidation with H-Glu(OtBu)-OtBu without further purification.

((Glu(OtBu)-OtBu)-PEG_{24})$_2$-Ph-azide (**9**)

To a solution of compound **8** in DMF (5.0 mL) was added DIEA (197 μL, 1.13 mmol), H-Glu(OtBu)-OtBu (100 mg, 339 μmol) and 1-[Bis(dimethylamino)methylene]-1H-1,2,3-triazolo[4,5-b]pyridinium 3-Oxide Hexafluorophosphate (HATU, 258 mg, 679 μmol). After stirring for 3 h at room temperature, chloroform and 10% (wt) aqueous citrate acid were added to the reaction mixture. The combined organic layers were washed with water and brine, dried with $MgSO_4$ and concentrated *in vacuo*. ^{t}Bu Protected Glu modified **8** was obtained (415 mg) as a crude compound.

Next, the deprotection of Cbz group in ^{t}Bu Protected Glu modified **8** was performed using Pd/C (50 mg) under H_2 atmosphere. After filtration and concentration of the reaction mixture, DIEA (196 μL, 1.13 mmol) and 2,5-dioxopyrrolidin-1-yl 6-azidohexanoate (81.4 mg, 248 μmol) were added to a solution of azide reduced compound in THF (5.0 mL). After stirring for 3 h at room temperature, chloroform and 10% (wt) aqueous citrate acid were added to the reaction mixture. The combined organic layers were washed with water and brine, dried with $MgSO_4$ and concentrated *in vacuo*. The residue was separated by silica gel chromatography (chloroform/methanol: 98/2 to 90/10) to obtain compound **9** (89.7 mg, 26% for 5 steps from compound **6**).

^{1}H-NMR ($CDCl_3$) δ: 1.44 (18H, s), 1.46 (18H, s), 1.62-1.67 (4H, m), 1.86-1.89 (2H, m), 2.20-2.36 (10H, m), 2.51 (4H, t, *J* = 5.8 Hz), 3.26 (2H, t, *J* = 7.0 Hz), 3.63-3.65 (194H, m), 3.73-3.76 (4H, m), 4.14 (2H, t, *J* = 5.1 Hz), 4.46-4.50 (2H, m), 7.60 (2H, dd, *J* = 1.0, 0.5 Hz), 7.92 (1H, br s). ESI-MS *m/z*: 1533 $[M - 2H]^{2-}$

<u>(Glu-PEG_{24})$_2$-Ph-azide (**10**)</u>

To a cooled solution of compound **9** (89.7 mg, 29.0 μmol) in DCM (8 mL) was added trifluoro acetic acid (2 mL). After stirring for 3 h at room temperature, the reaction mixture was concentrated *in vacuo*. Diethyl ether was added to the residue, and the white precipitate was isolated as the deprotected compound (72.4 mg, 87%). Compound **10** was subjected to amidation with compound **4a** without further purification.

<u>Chemically modified mannose linker (CMML4: **11**)</u>

To a solution of compound **10** (4.0 mg, 1.3 μmol) in *N*,*N*-dimethylacetamide (0.4 mL) was added DIEA (5.0 μL, 29 μmol), HATU (4.2 mg, 11 μmol) and compound **3a** (6.7 mg, 6.5 μmol). After stirring overnight at room temperature, the reaction mixture was purified by prep. HPLC. After the concentration of the isolated fraction, a reaction of the isolated compound with 5 M sodium methoxide solution in methanol (5 mL) at room temperature for 3 h gave the crude compound **11**. The crude compound was purified by prep. HPLC to afford purified compound **11** (0.3 mg, 4.0%).

^{1}H-NMR (400 MHz, D_2O) δ: 1.19-1.27 (2H, m), 1.42-1.57 (4H, m), 1.79-1.92 (18H, m), 2.01-2.10 (2H, m), 2.20-2.53 (10H, m), 2.76-2.85 (8H, m), 3.11 (2H, t, J = 6.8 Hz), 3.31-3.36 (8H, m), 3.56-3.74 (222H, m), 3.87-3.88 (8H, m), 3.96-3.98 (8H, m), 4.14-4.22 (20H, m), 4.98 (4H, s), 6.79 (16H, dd, J = 8.1, 3.5 Hz), 7.06 (16H, dd, J = 8.1, 4.6 Hz), 7.52 (2H, s), 7.80 (1H, s).

ESI-MS *m/z*: 1322.92 $[M + 4H]^{4+}$

Synthesis of monovalent chemically modified mannose linker (CMML1: 13)

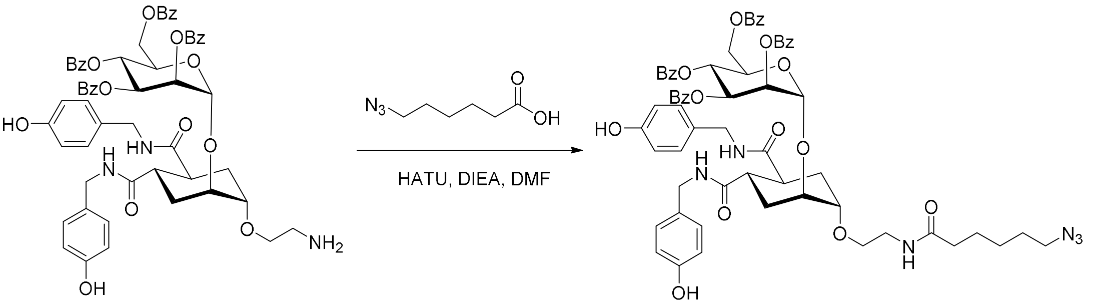

3a **12**

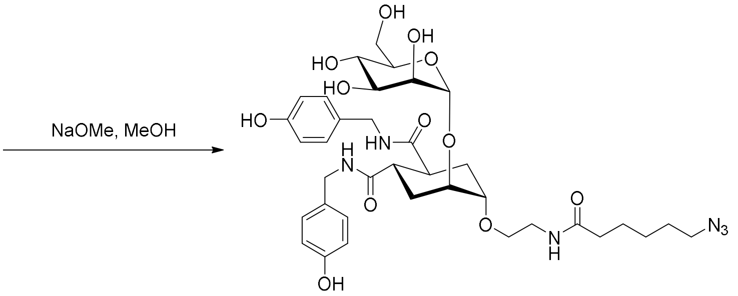

13

To a 0.1 M stirred solution of compound **3a** (50.0 mg, 48.0 μmol) in DMF (965 μL), we added *N*, *N*-diisopropylethylamine (25.3 μL, 145 μmol), 6-azidohexanoic acid (8.47 μL, 58.0 μmol), and HATU (22.0 mg, 55 μmol) at room temperature, and the reaction mixture was stirred at the same temperature for 1 h. The reaction was monitored by LC-MS analysis. After completion of the reaction, saturated $NaHCO_3$ aq. was added to the reaction mixture. The aqueous phase was extracted with ethyl acetate. The organic layers were washed with saturated NH_4Cl aq., water, and brine, dried with Na_2SO_4, and concentrated *in vacuo*. The residue was dissolved in methanol (864 μL), and 5 M sodium methoxide in methanol (96.0 μL, 480 μmol) was added. The reaction was monitored by LC-MS analysis. After completion of the reaction, 6N HCl was added to the reaction mixture to approximately pH 7. The solution was concentrated *in vacuo* and purified by preparative HPLC. The pure fractions were combined and lyophilized to obtain compound **13** as a white solid (3.1 mg, 4.1 μmol, 8.5% over two steps).

^{1}H NMR (400 MHz, DMSO-d6) δ: 1.25-1.29 (2H, m), 1.49 (4H, dt, J = 21.9, 7.0 Hz), 1.71-1.74 (4H, br m), 2.07 (2H, t, J = 7.4 Hz), 2.73-2.75 (2H, m), 3.19 (2H, dd, J = 11.5, 5.7 Hz), 3.28 (2H, t, J = 6.8 Hz), 3.48-3.51 (11H, m), 3.66 (1H, dd, J = 11.4, 1.3 Hz), 3.70 (1H, dd, J = 3.2, 1.6 Hz), 3.80 (1H, d, J = 2.8 Hz), 4.07 (4H, d, J = 5.8 Hz), 4.77 (1H, d, J = 1.5 Hz), 6.66 (4H, dt, J = 9.1, 2.4 Hz), 6.99 (4H, dd, J = 8.6, 2.0 Hz), 7.77 (1H, t, J = 5.7 Hz), 8.08 (1H, t, J = 6.0 Hz), 8.20 (1H, t, J = 6.1 Hz), 9.22 (2H, br s). ^{13}C NMR (101 MHz, DMSO-d6) δ: 24.8, 25.8, 28.0, 35.2, 38.8, 41.4, 50.6, 61.4, 67.0, 67.1, 70.6, 71.0, 74.4, 74.6, 98.7, 114.9, 128.3, 129.9, 129.9, 156.1,172.3, 174.0, 174.1. ESI-MS *m*/*z*: 759.59 $[M + H]^+$

Synthesis of Man-Lys(Man)-PEG3-biotin (17)

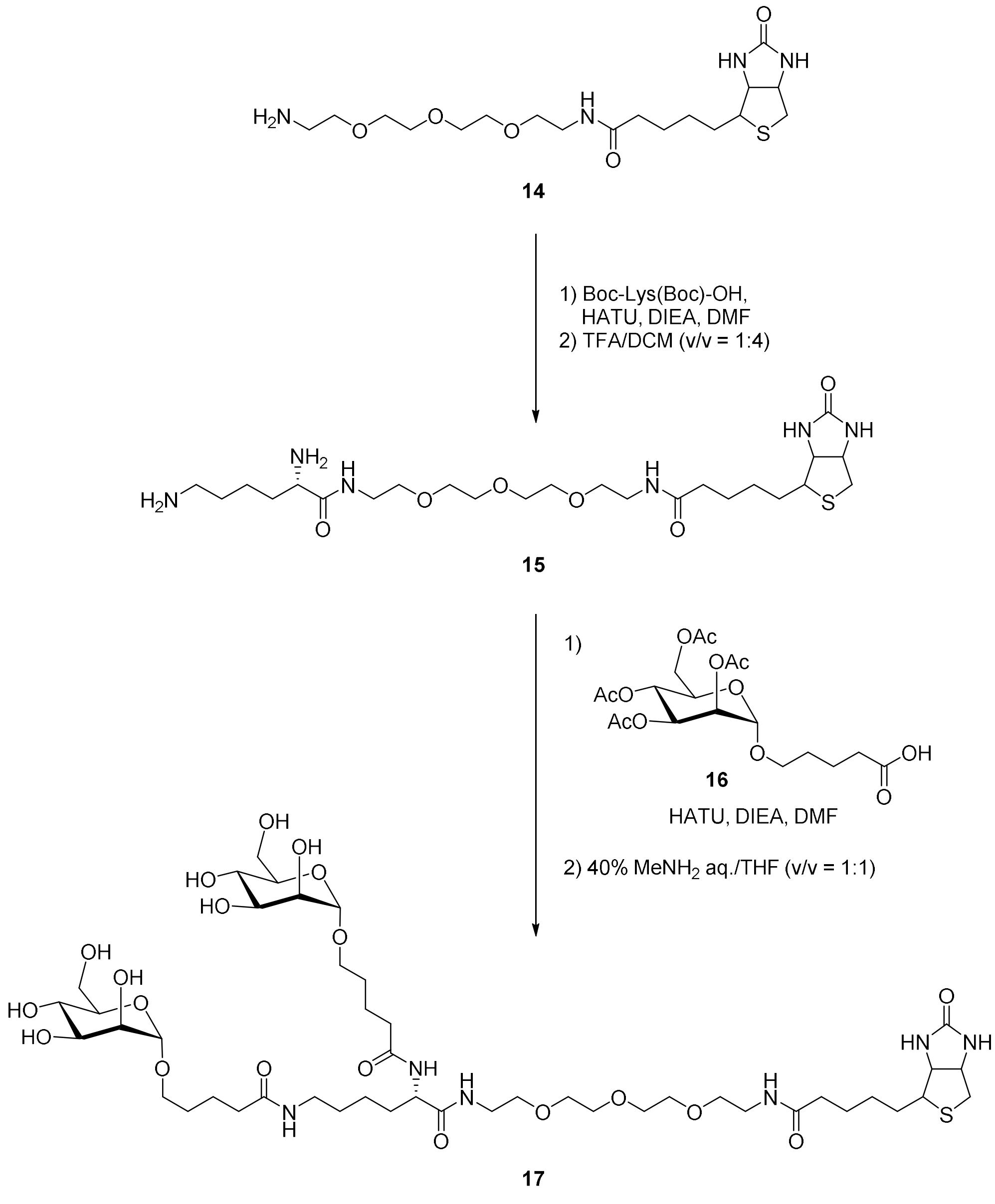

Lys-PEG3-biotin (**15**)

To a solution of Biotin-PEG3-Amine (**14**: 100 mg, 239 μmol) in DMF (5 mL) was added DIEA (209 μL, 1.20 mmol), followed by the addition of HATU (184 mg, 484 μmol) and Boc-Lys(Boc)-OH (166 mg, 479 μmol). After stirring overnight at room temperature, the reaction mixture was extracted by chloroform and concentrated *in vacuo*. The combined organic layers were washed with water and brine, dried with $MgSO_4$ and concentrated *in vacuo*. The residue was separated by silica gel chromatography (chloroform/methanol: 98/2 to 90/10) to obtain Boc-Lys(Boc)-PEG3-biotin.

Next, to a solution of Boc-Lys(Boc)-PEG3-biotin in DCM (6 mL) was added TFA (1.5 mL). After stirring overnight at room temperature, the reaction mixture was concentrated *in vacuo*. Compound **15** was

obtained (131 mg, 42%) and subjected to amidation with mannose derivative (**16**) without further purification.

ESI-MS *m*/*z*: 548 $(M + H)^+$

Man-Lys(Man)-PEG3-biotin (**17**)

To a solution of **15** (26 mg, 20 μmol) in DMF (0.8 mL) was added DIEA (34 μL, 200 μmol), followed by the addition of HATU (24 mg, 62 μmol) and **16** (19 mg, 42 μmol). After stirring for 2 h at room temperature, the reaction mixture was extracted by chloroform. The combined organic layers were washed with water and brine, dried with $MgSO_4$ and concentrated *in vacuo*. The residue was separated by silica gel chromatography (chloroform/methanol: 98/2 → 90/10) to obtain acetyl protected Man-Lys(Man)-PEG3-biotin.

Next, to a solution of acetyl protected Man-Lys(Man)-PEG3-biotin in THF (0.5 mL) was added 40% methylamine aqueous solution (0.5 mL). After stirring for 2 h at room temperature, the reaction mixture was concentrated *in vacuo*. The residue was purified by prep. HPLC to afford purified compound **17** (6.8 mg, 50%)

^{1}H NMR (400 MHz, D_2O) δ: 1.32-1.82 (20H, m), 2.27-2.33 (6H, m), 2.79 (1H, d, J = 13.1 Hz), 3.00 (1H, dd, J = 13.1, 5.0 Hz), 3.19 (2H, t, J = 6.8 Hz), 3.32-3.48 (5H, m), 3.55-3.58 (2H, m), 3.62-3.70 (16H, m), 3.75-3.80 (6H, m), 3.89-3.90 (2H, m), 3.94 (2H, dd, J = 3.0, 1.5 Hz), 4.24 (1H, dd, J = 8.6, 5.9 Hz), 4.43 (1H, dd, J = 7.9, 4.5 Hz), 4.62 (1H, dd, J = 7.9, 4.8 Hz), 4.86 (2H, s). ESI-MS *m*/*z*: 1071.89 $[M + H]^+$

Analytical data

^{1}H NMR chart of compound **4a**

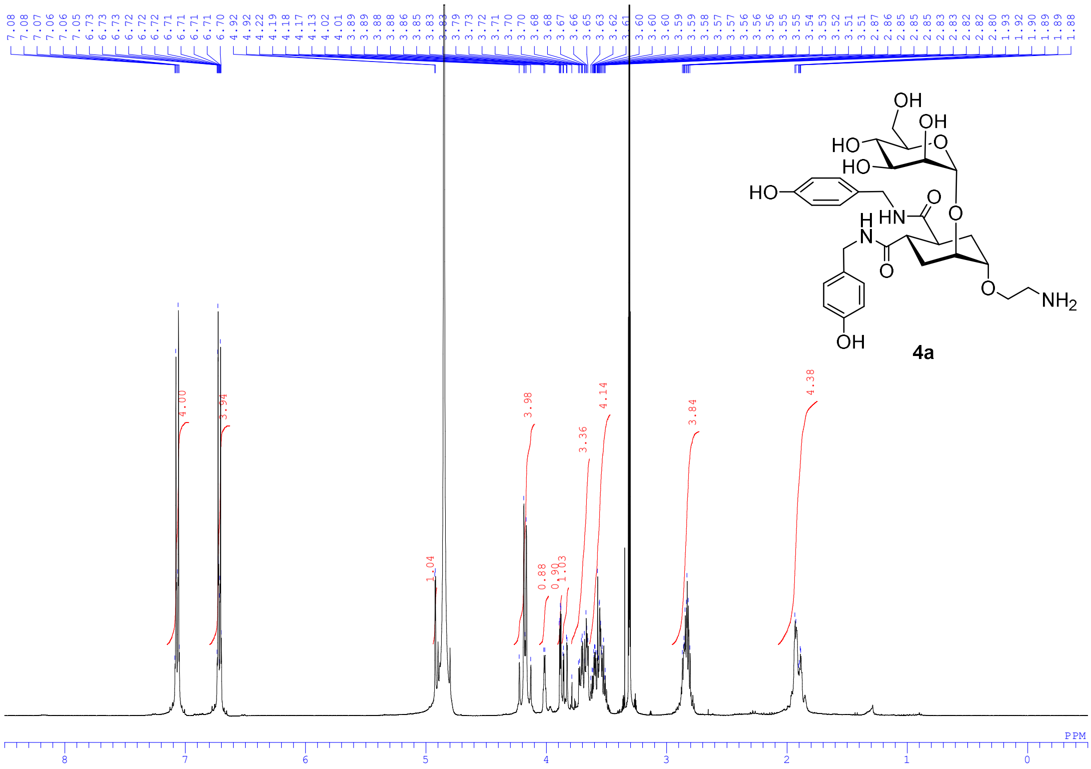

^{13}C NMR chart of compound **4a**

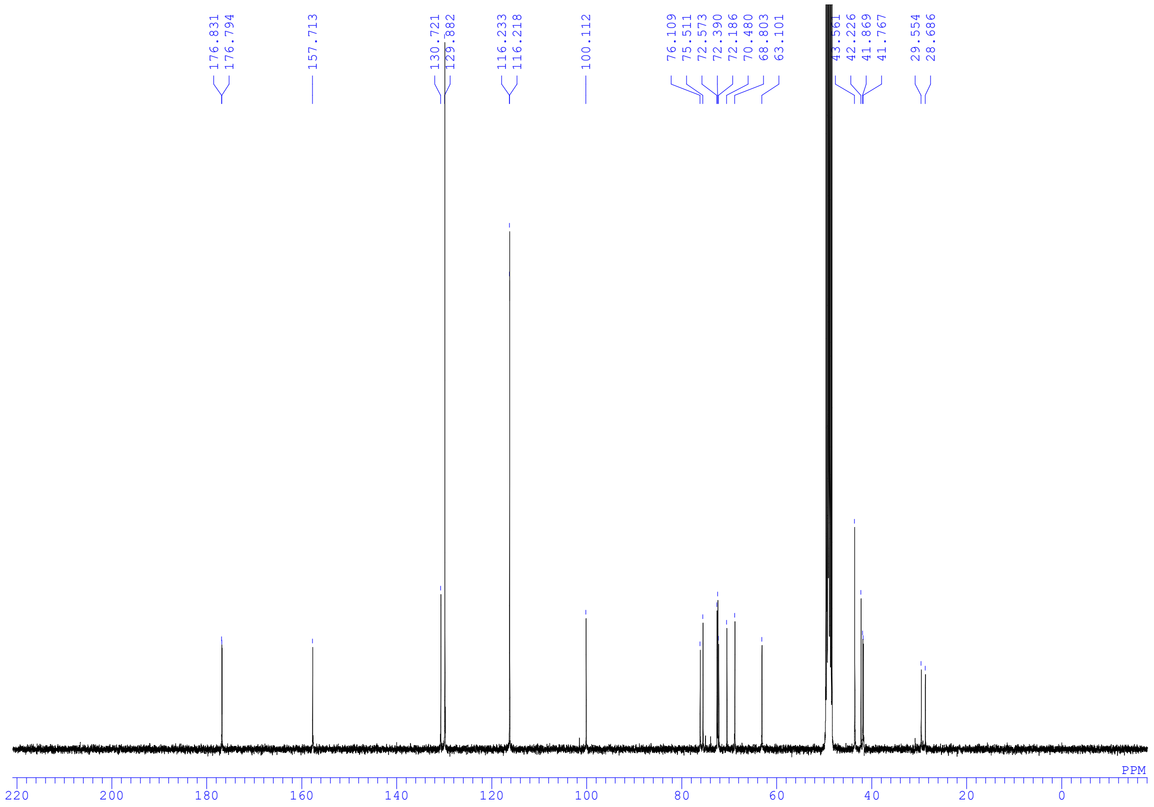

^{1}H NMR chart of compound **4b**

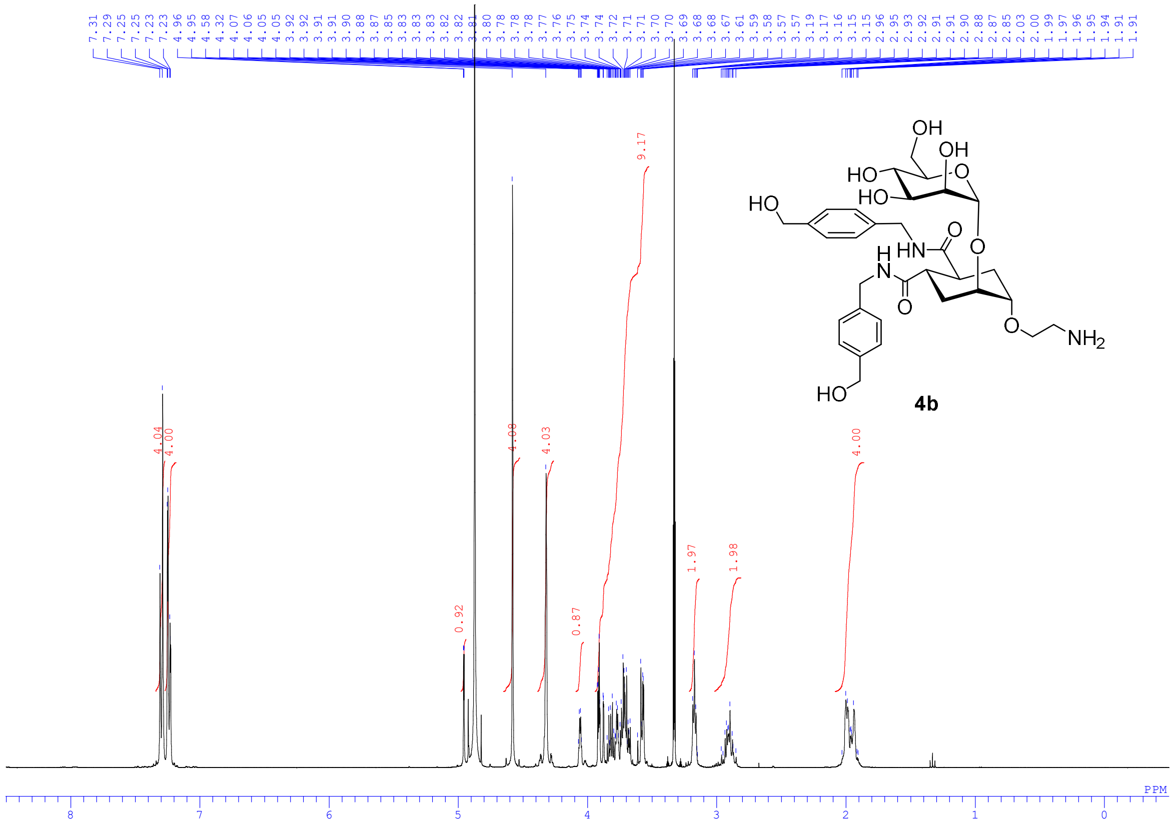

4b

^{13}C NMR chart of compound **4b**

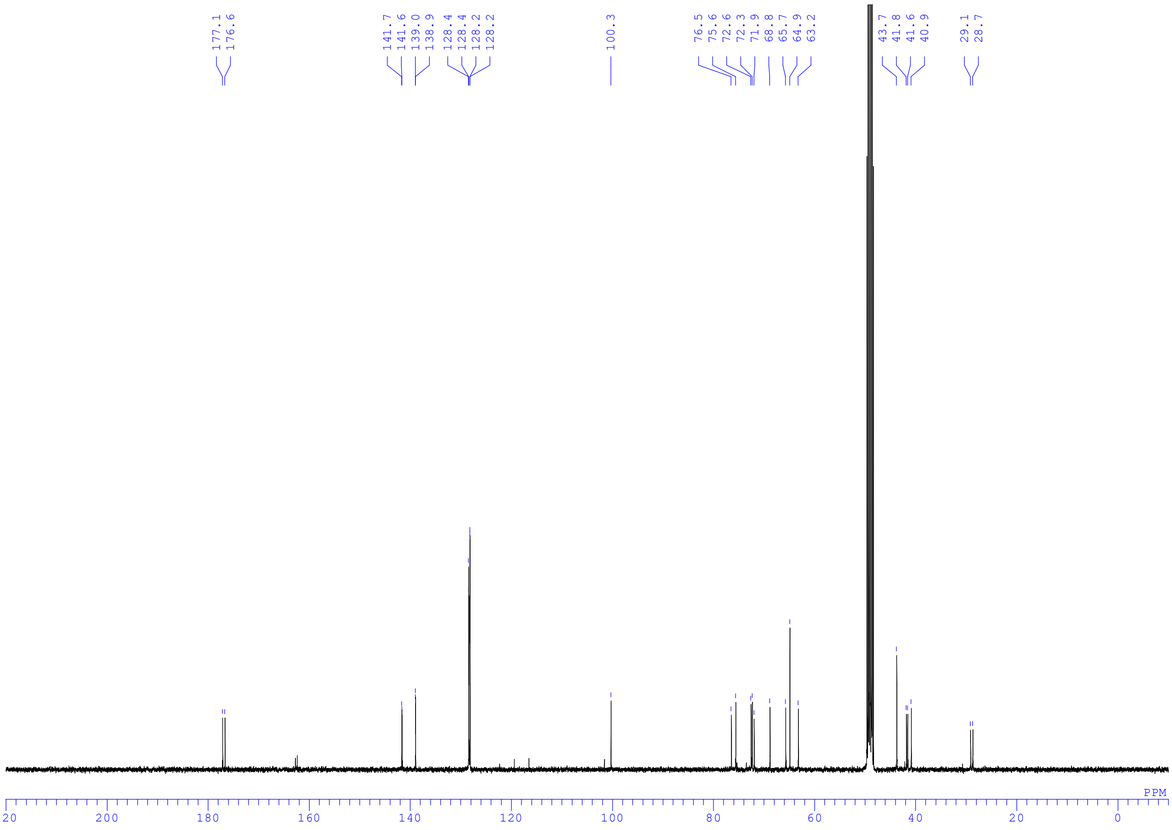

^{1}H NMR chart of compound **4c**

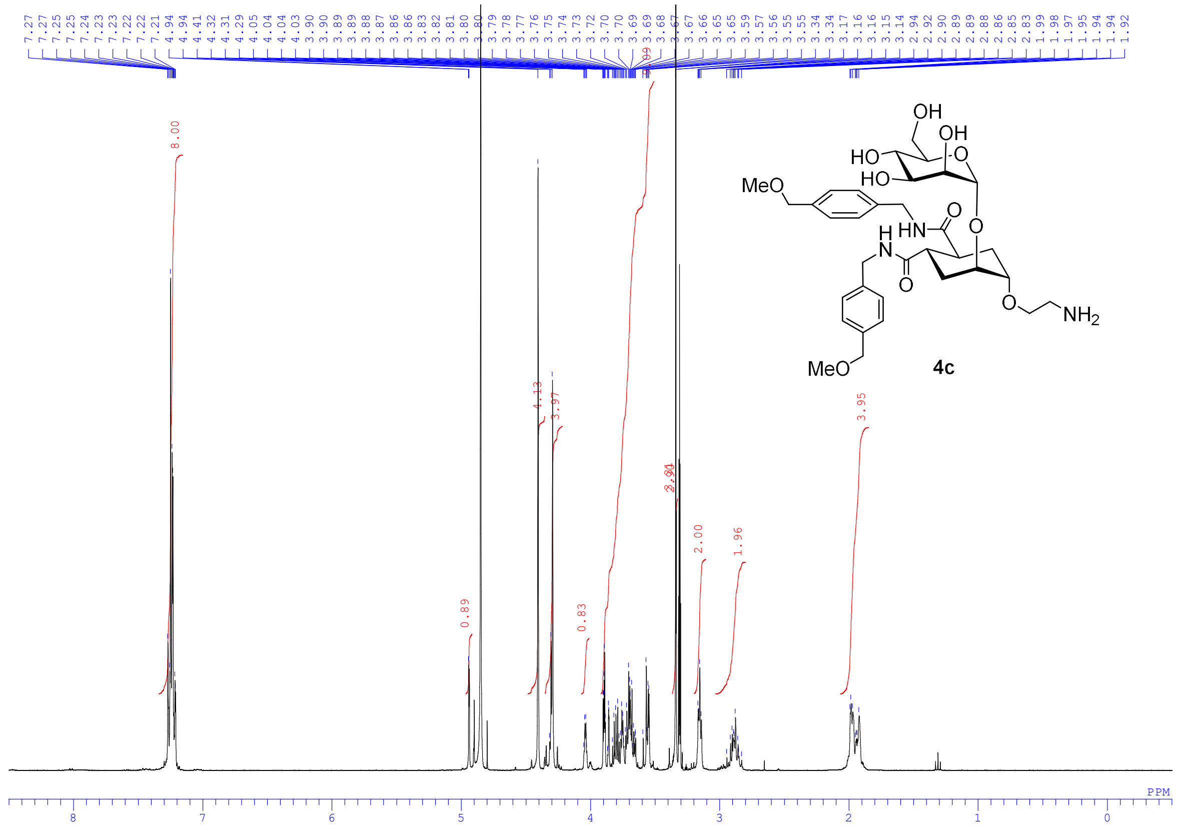

^{13}C NMR chart of compound **4c**

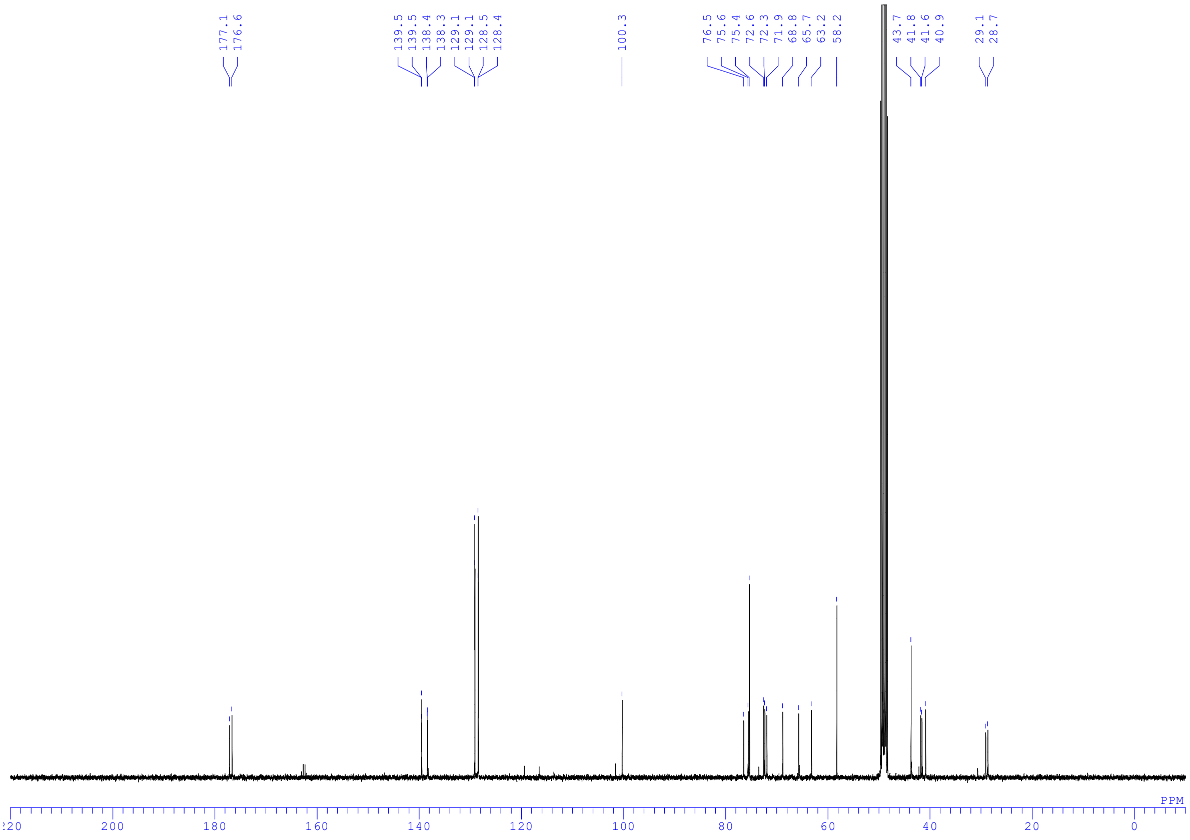

^{1}H NMR chart of compound **4d**

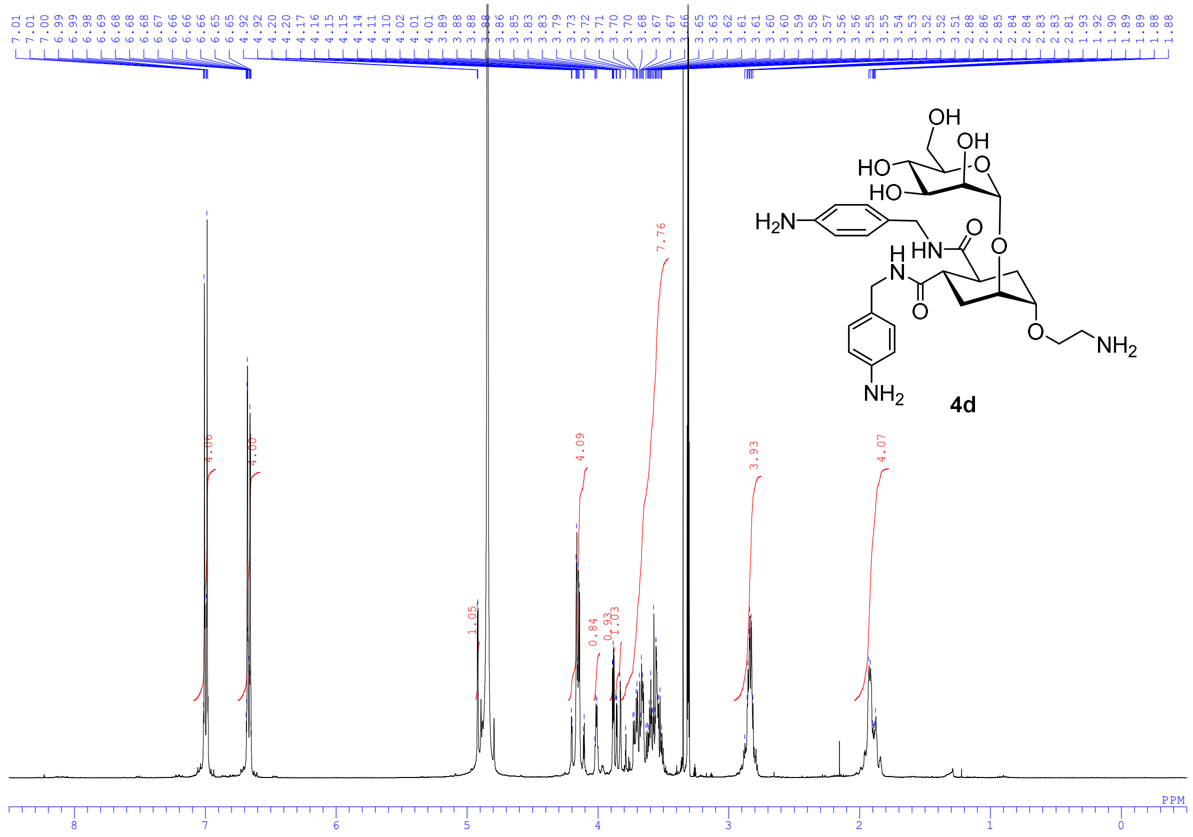

^{13}C NMR chart of compound **4d**

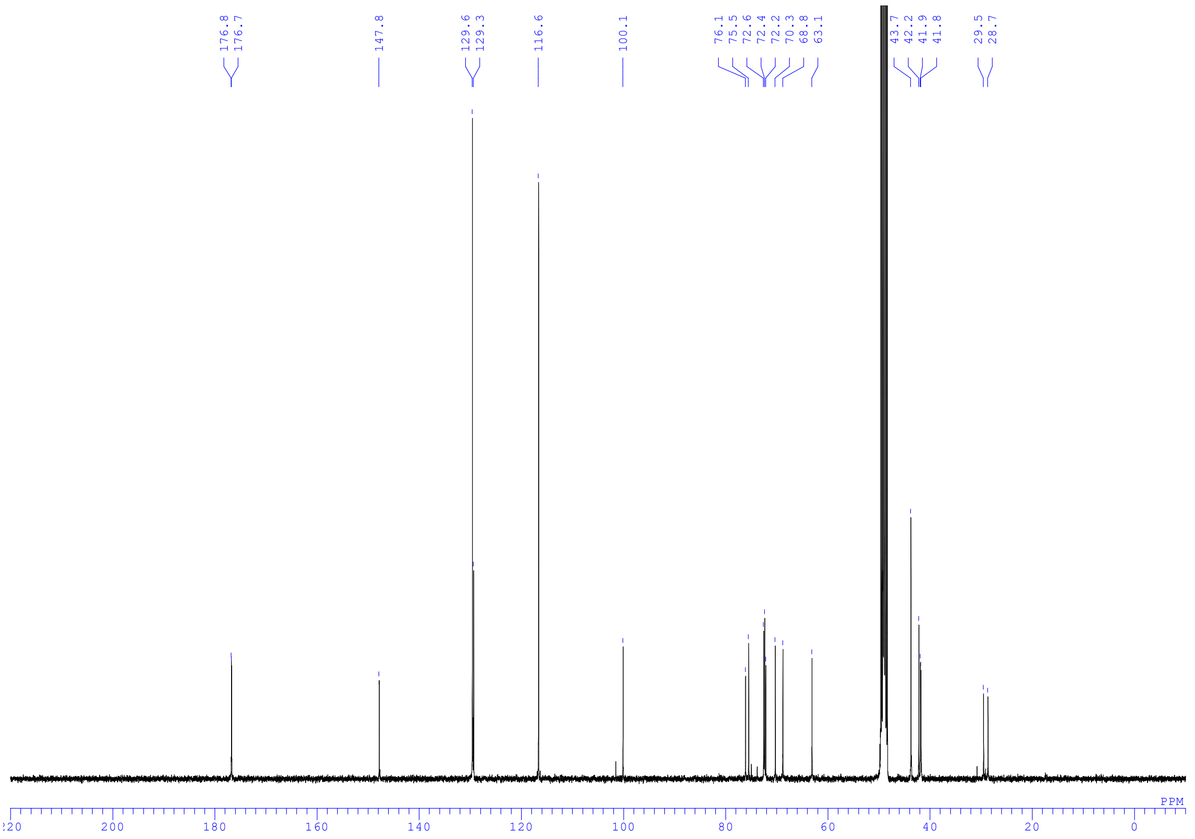

^{1}H NMR chart of compound **4e**

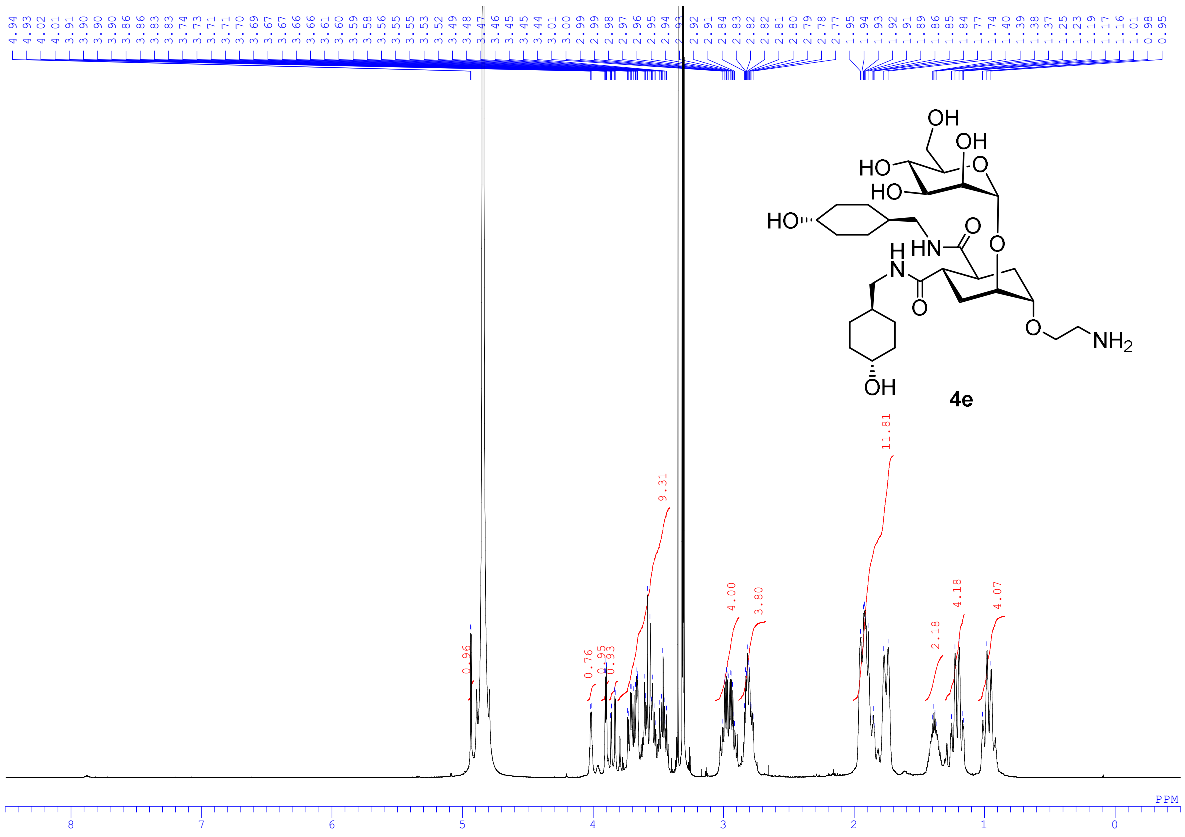

^{13}C NMR chart of compound **4e**

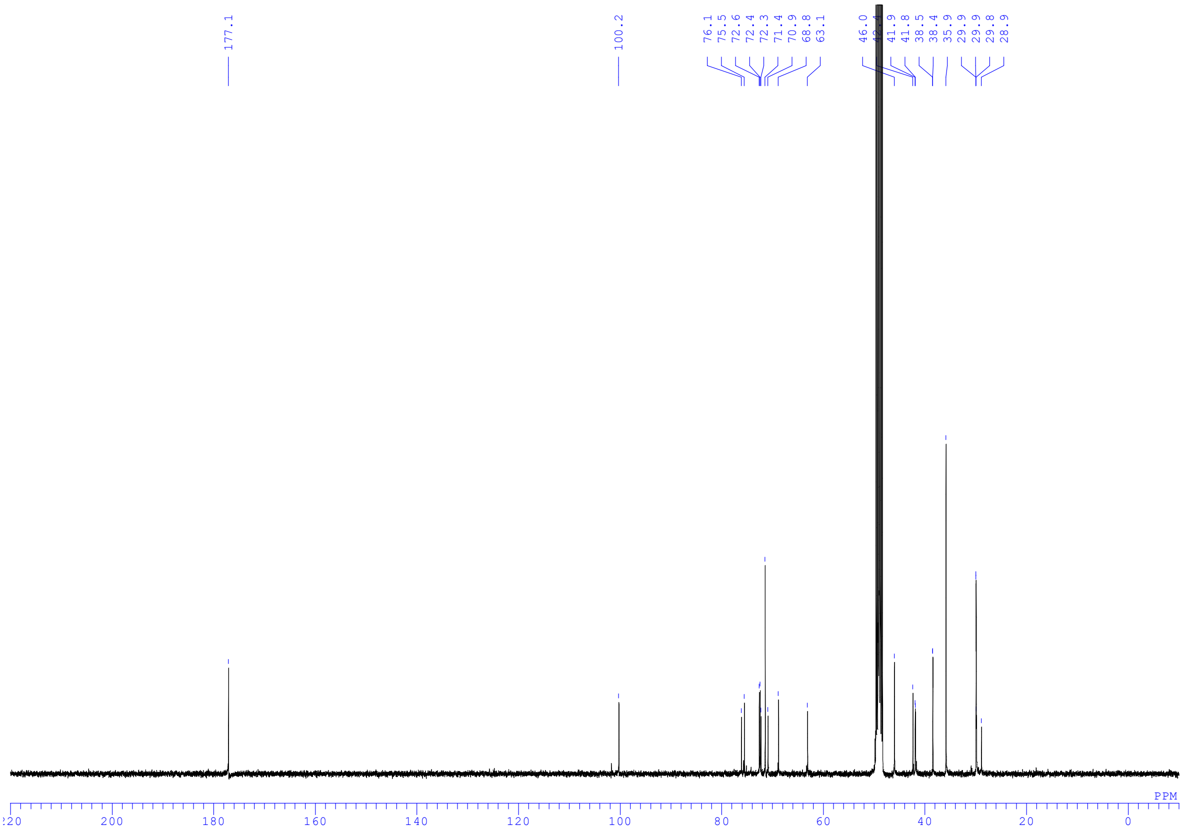

^{1}H NMR chart of compound **4f**

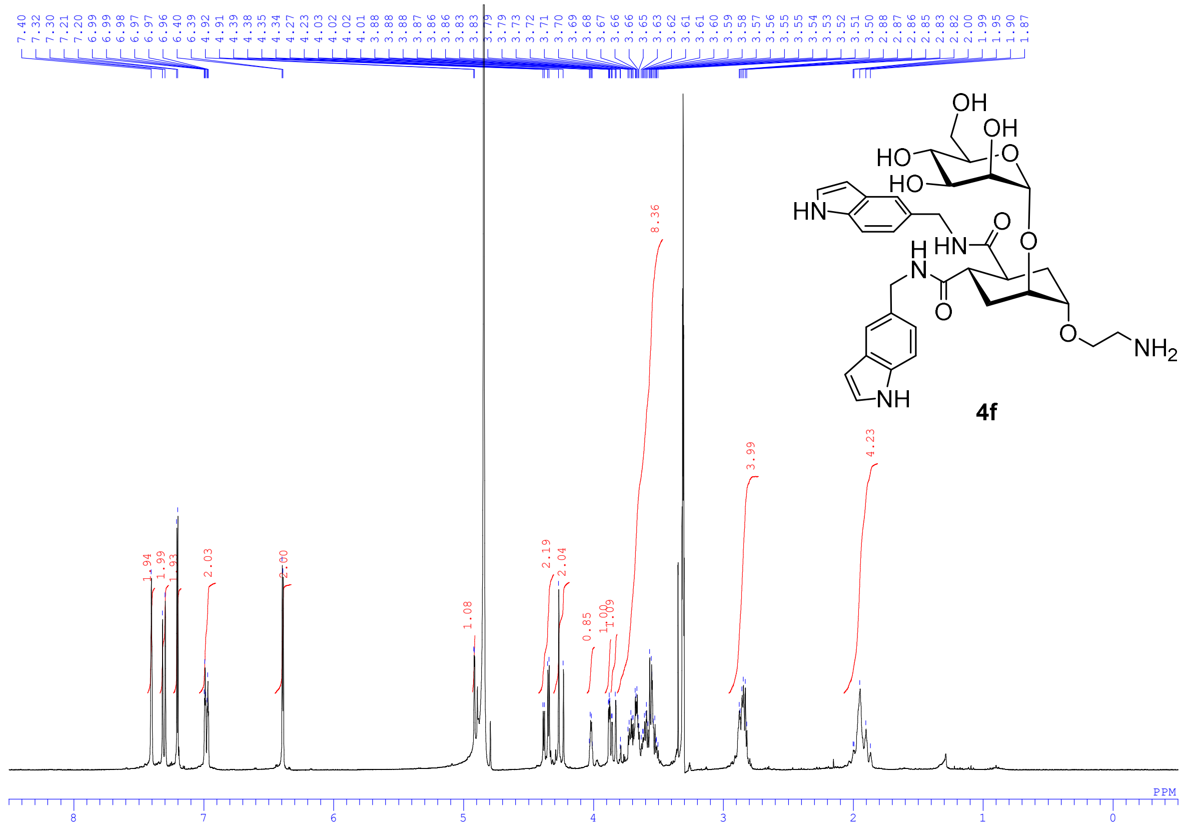

^{13}C NMR chart of compound **4f**

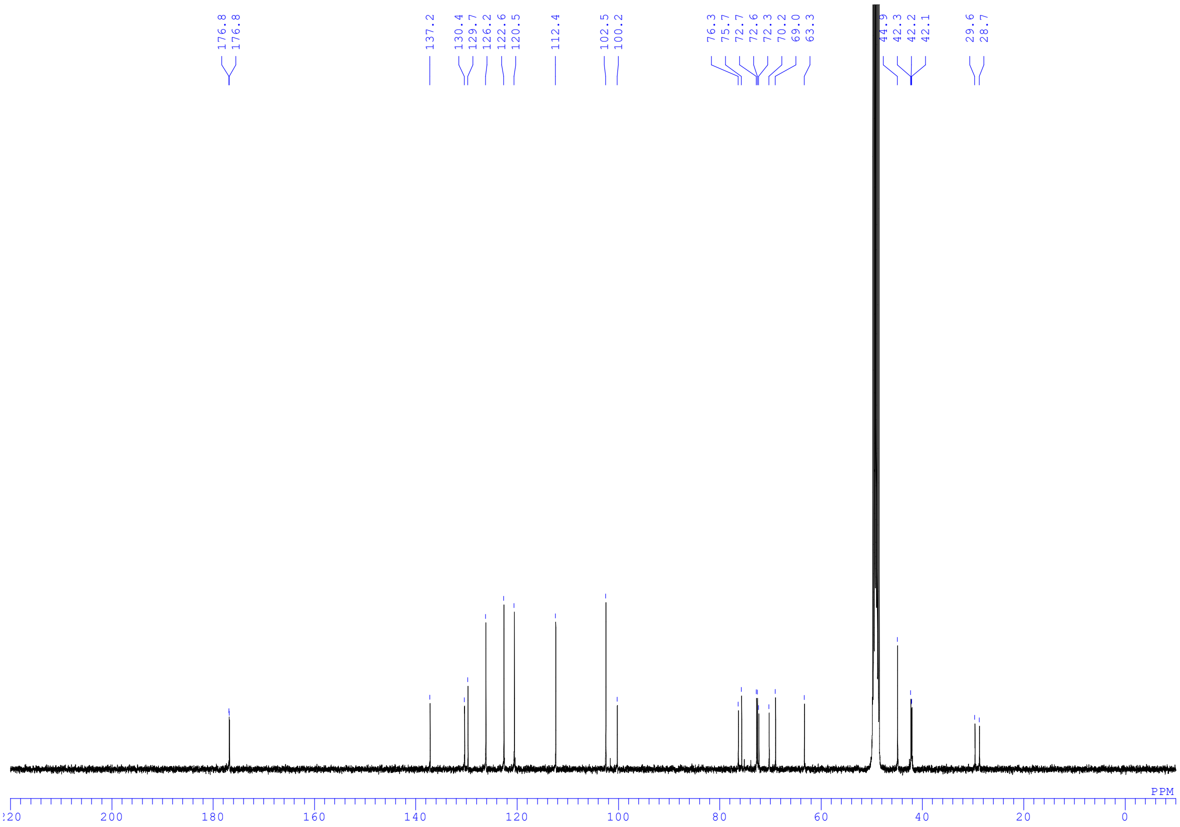

^{1}H NMR chart of chemically modified mannose linker (**CMML4**: **11**)

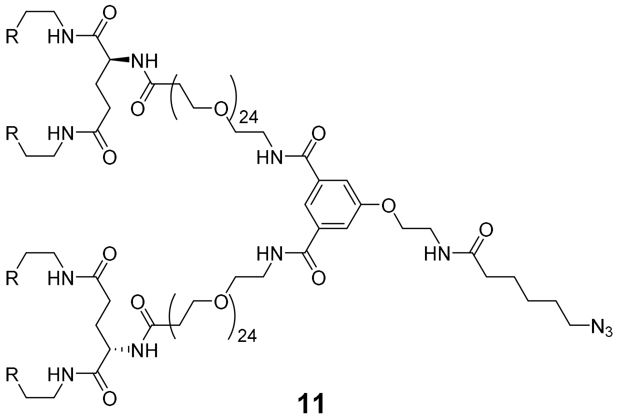

11
(CMML4)

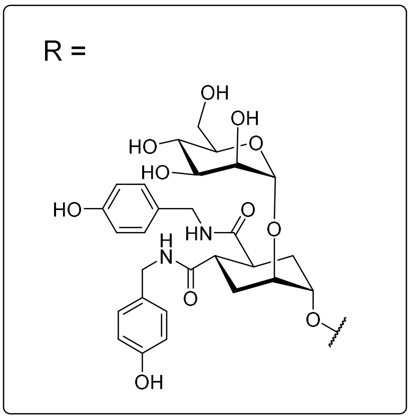

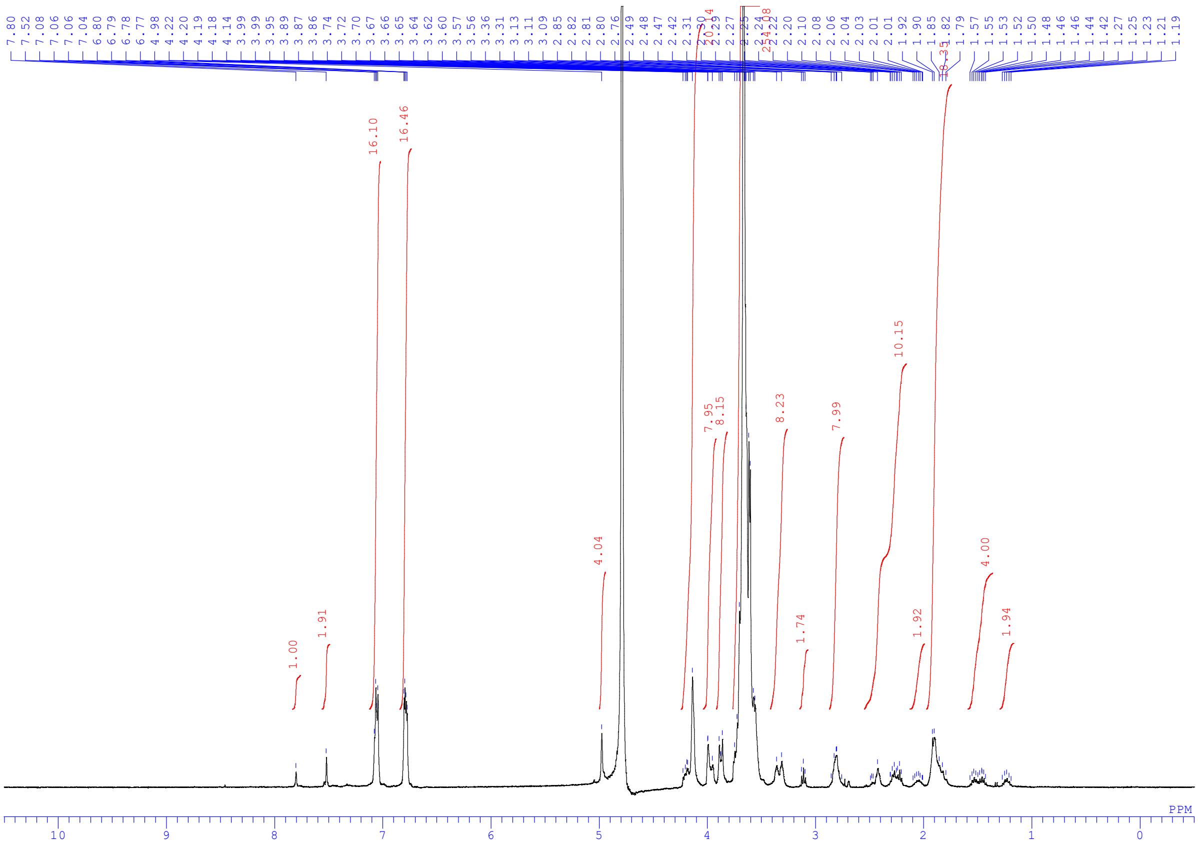

^{1}H NMR chart of monovalent chemically modified mannose linker (CMML1: **13**)

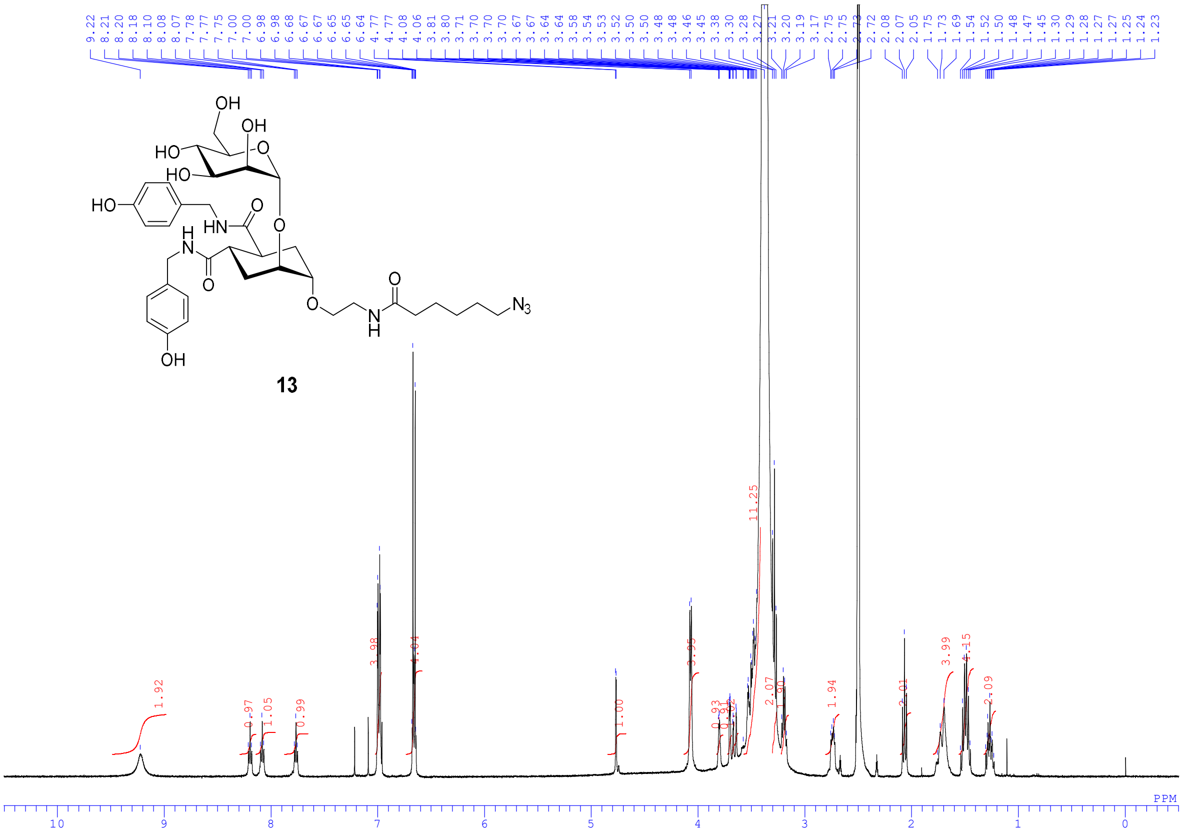

^{13}C NMR chart of monovalent chemically modified mannose linker

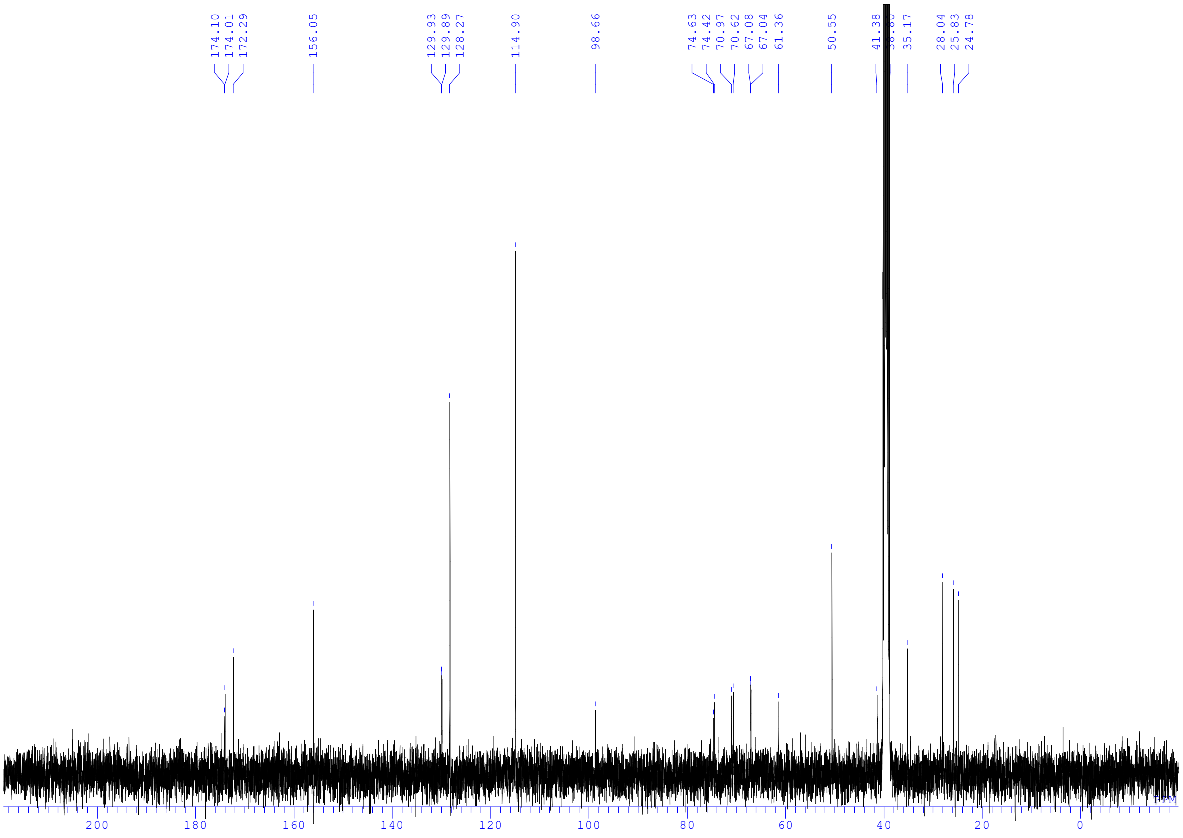

^{1}H NMR chart of Man-Lys(Man)-PEG3-biotin

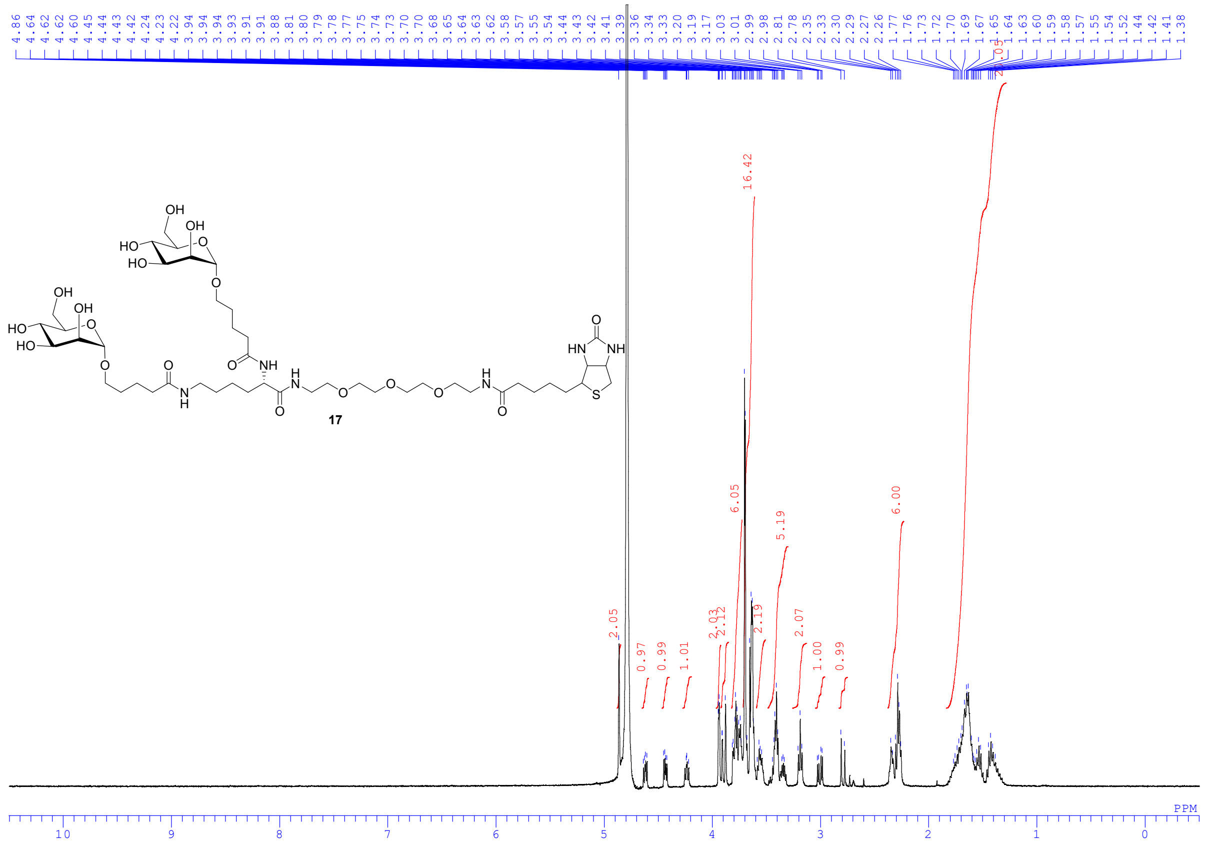

LC-MS of CMML4 (Detection: 276 nm)

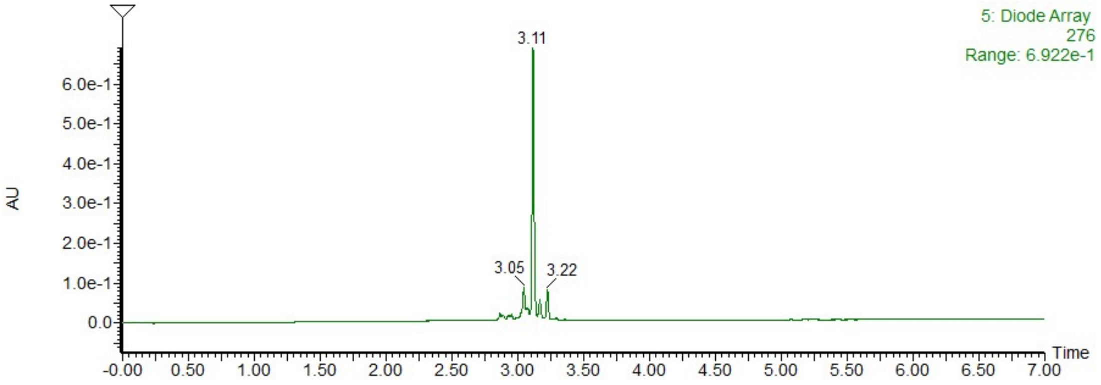

LC-MS of CMML1 (Detection: 276 nm)

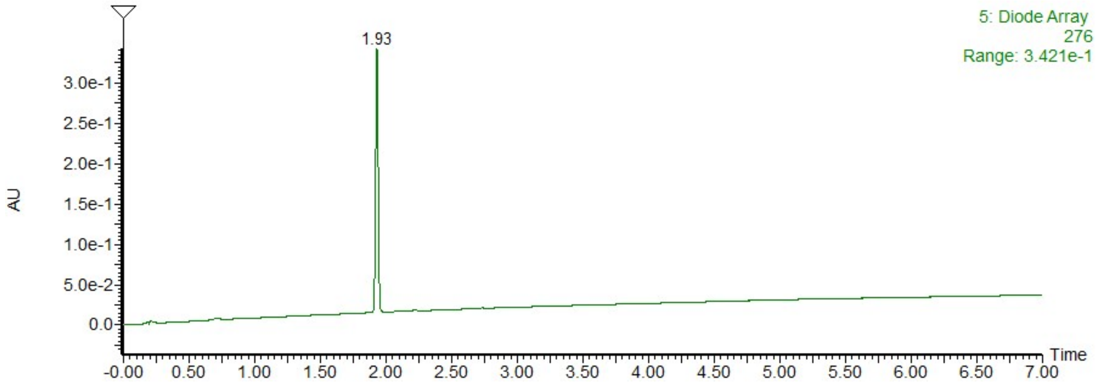

LC-MS of Man-Lys(Man)-PEG3-biotin (Detection: ELSD)

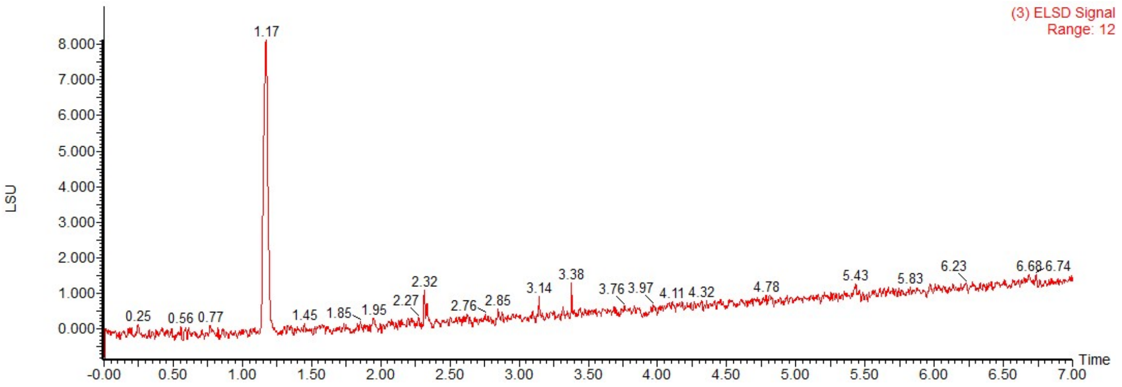

LC-MS analysis of CMM-oligonucleotides

Oligonucleotides were analyzed by Agilent Infinity 1260 LC-MS system.

LC-MS analysis (LC condition 1)

Column: ACQUITY UPLC Oligonucleotide BEH C18 (130Å, 1.7 μm, 2.1 x 50 mm, Waters), Buffer A: 8.6 mM TEA/100 mM HFIP, Buffer B: MeCN, Program: a linear gradient of 10-90% buffer B over 9 min followed by 10% buffer B over 3 min, flow rate: with 0.6 mL/min, Column Temperature: 60 °C.

LC-MS analysis (LC condition 2)

Column: ACQUITY UPLC Oligonucleotide BEH C18 (130Å, 1.7 μm, 2.1 x 100 mm, Waters), Buffer A: 8.6 mM TEA/100 mM HFIP, Buffer B: MeCN, Program: a linear gradient of 10-90% buffer B over 16 min followed by 10% buffer B over 4 min, flow rate: with 0.3 mL/min, Column Temperature: 60 °C.

LC-MS analysis (LC condition 3)

Column: ACQUITY UPLC Oligonucleotide BEH C18 (130Å, 1.7 µm, 2.1 x 75 mm, Waters), Buffer A: 8.6 mM TEA/100 mM HFIP, Buffer B: MeCN, Program: a linear gradient of 10-90% buffer B over 18 min followed by 10% buffer B over 4 min, flow rate: with 0.4 mL/min, Column Temperature: 60 °C.

LC-MS of CMM4-siHPRT1-a passenger strand (LC conditions 1)

calcurated mass: 12549.61, observed mass: 12549.42

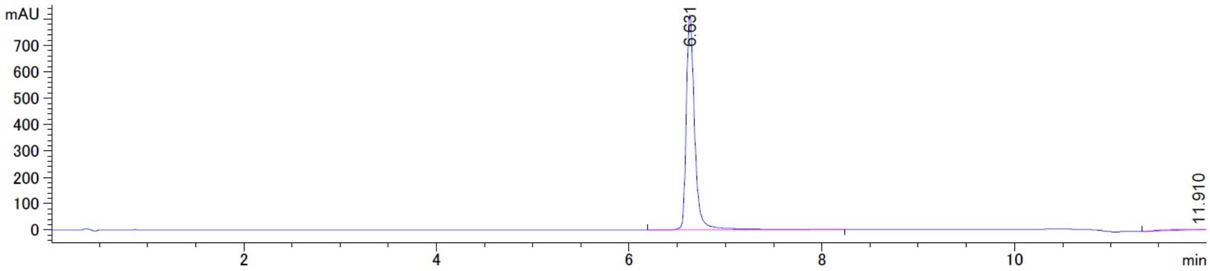

LC-MS of CMM4-siHPRT1-b passenger strand (LC conditions 2)

calcurated mass: 12633.86, observed mass: 12635.07

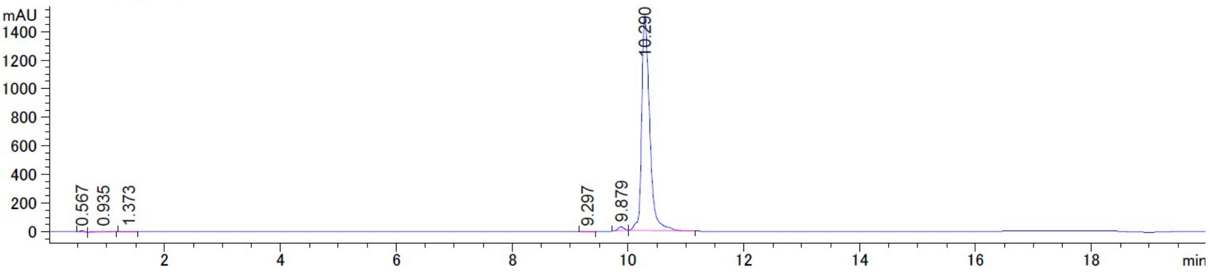

LC-MS of CMM4-siHPRT1-c passenger strand (LC conditions 2)

calcurated mass: 12645.89, observed mass: 12647.03

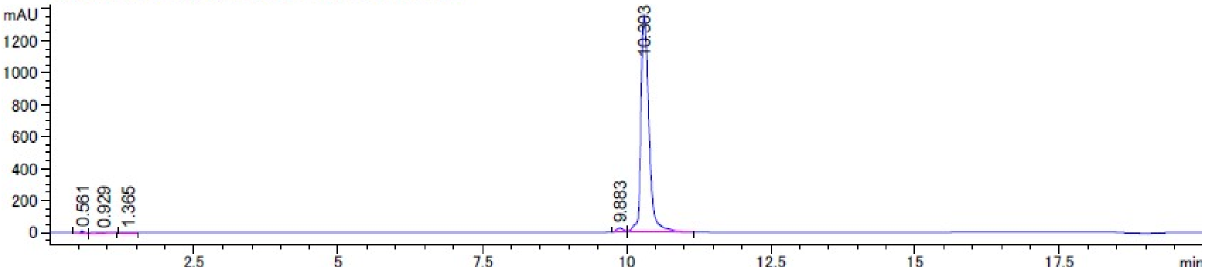

LC-MS of CMM4-siB2M(h) passenger strand (LC conditions 1)

calcurated mass: 12540.60, observed mass: 12540.18

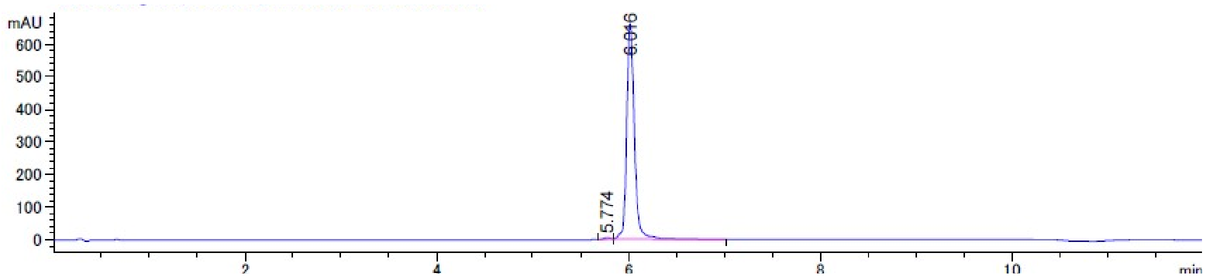

LC-MS of CMM4-siB2M(m) passenger strand (LC conditions 1)

calcurated mass: 12564.62, observed mass: 12564.41

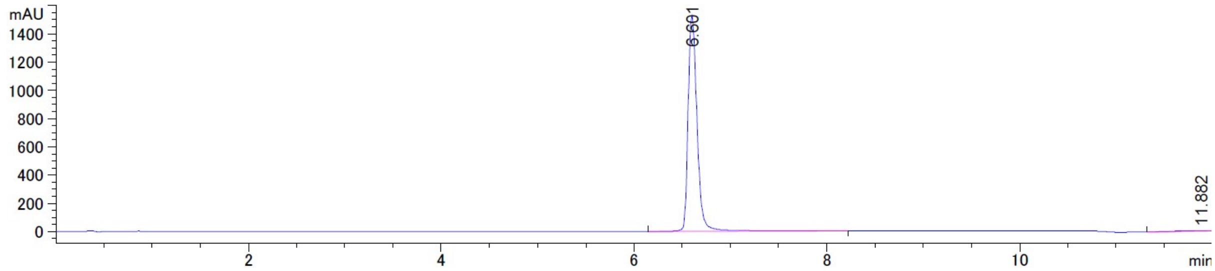

LC-MS of CMM4-siCD45 passenger strand (LC conditions 1)

calcurated mass: 12626.70, observed mass: 12626.46

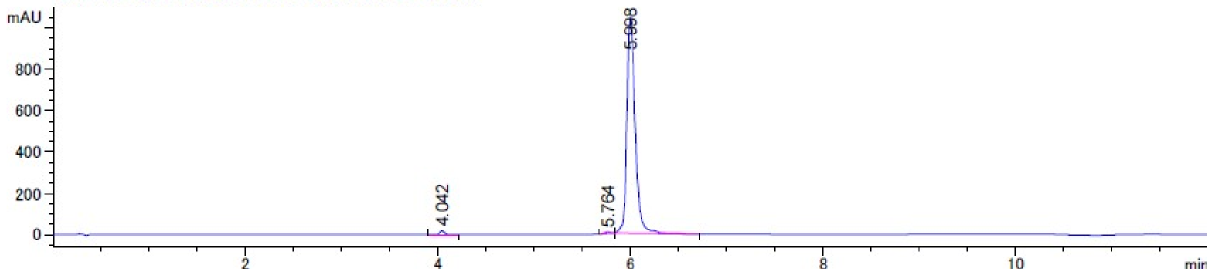

LC-MS of CMM1-siHPRT1-a passenger strand (LC conditions 1)

calcurated mass: 8022.58, observed mass: 8022.42

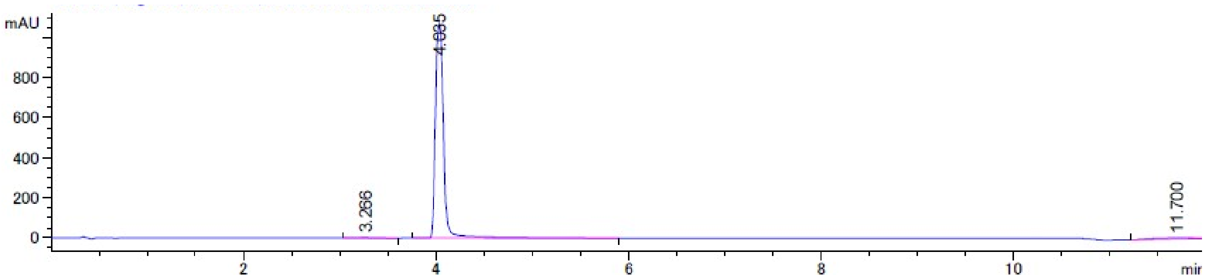

LC-MS of CMM1-siB2M(h) passenger strand (LC conditions 1)

calcurated mass: 8013.57, observed mass: 8013.45

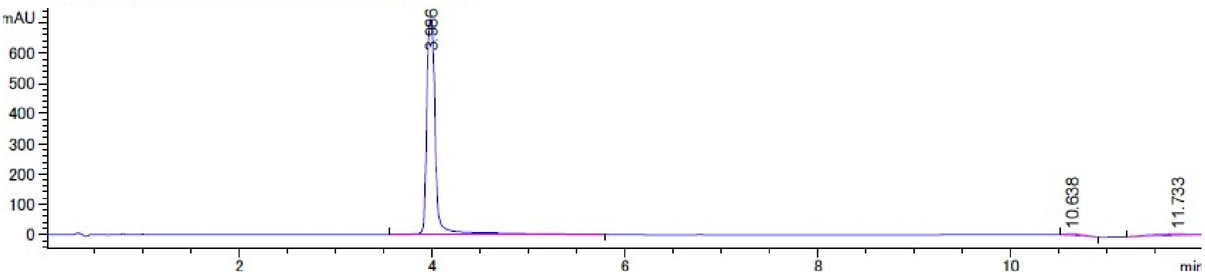

LC-MS of GalNAc4-siHPRT1 passenger strand (LC conditions 3)

calcurated mass: 11352.50, observed mass: 11353.31

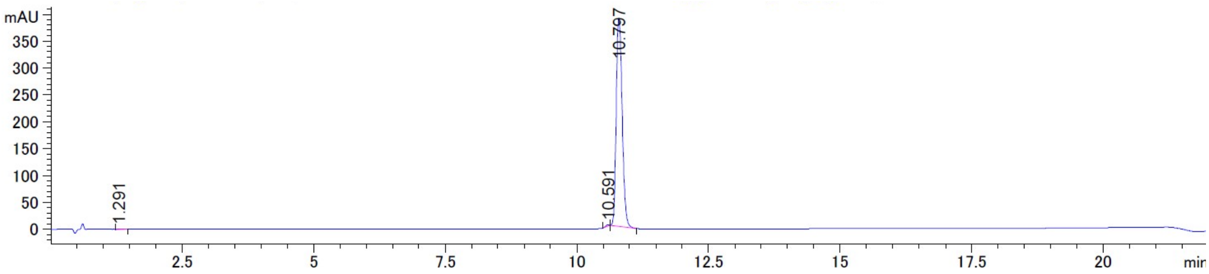

Supplementary References

1. Varga, N. Sutkeviciute, I. Guzzi, C. McGeagh, J. Petit-Haertlein, I. Gugliotta, S. Weiser, J. Angulo, J. Fieschi, F. Bernardi, A. (2013) Selective targeting of dendritic cell-specific intercellular adhesion molecule-3-grabbing nonintegrin (DC-SIGN) with mannose-based glycomimetics: Synthesis and interaction studies of bis(benzylamide) derivatives of a pseudomannobioside. *Chem. Eur. J.,* **19**, 4786 – 4797.
2. Zhang, J. J. Vargeese, C. Iwamoto, N. Shivalila, S. C. Kothari, N. Fdurbin, A. F. Ramasamy, S. Kandasamy, P. Kumarasamy, J. Bommineni, G. R. et al. (2019) Oligonucleotide compositions and methods of use thereof. WO2019200185.
